# Synthesis and Glycosidase Inhibition Properties of Calix[8]arene-Based Iminosugar Click Clusters

**DOI:** 10.3390/ph13110366

**Published:** 2020-11-05

**Authors:** Jérémy P. Schneider, Stefano Tommasone, Paolo Della Sala, Carmine Gaeta, Carmen Talotta, Céline Tarnus, Placido Neri, Anne Bodlenner, Philippe Compain

**Affiliations:** 1 Laboratoire d’Innovation Moléculaire et Applications (LIMA), University of Strasbourg | University of Haute-Alsace | CNRS (UMR 7042), Equipe de Synthèse Organique et Molécules Bioactives (SYBIO), ECPM, 25 Rue Becquerel, 67000 Strasbourg, France; jeremy.schneider@live.fr; 2 Laboratory of Supramolecular Chemistry (SupraLab@UniSa), Dipartimento di Chimica e Biologia “A. Zambelli”, Università di Salerno, Via Giovanni Paolo II 132, I-84084 Fisciano, Italy; s.tommasone@bham.ac.uk (S.T.); pdellasala@unisa.it (P.D.S.); ctalotta@unisa.it (C.T.); 3 Laboratoire Vigne Biotechnologies et Environnement EA-3991, University of Haute-Alsace, 33 rue de Herrlisheim, 68008 Colmar CEDEX, France; celine.tarnus@uha.fr

**Keywords:** multivalency, inhibitory multivalent effect, CuAAC, calix[8]arene, α-mannosidase

## Abstract

A set of 6- to 24-valent clusters was constructed with terminal deoxynojirimycin (DNJ) inhibitory heads through C6 or C9 linkers by way of Cu(I)-catalyzed azide-alkyne cycloaddition (CuAAC) reactions between mono- or trivalent azido-armed iminosugars and calix[8]arene scaffolds differing in their valency and their rigidity but not in their size. The power of multivalency to upgrade the inhibition potency of the weak DNJ inhibitor (monovalent DNJ *K_i_* being at 322 and 188 µM for C6 or C9 linkers, respectively) was evaluated on the model glycosidase Jack Bean α-mannosidase (JBα-man). Although for the clusters with the shorter C6 linker the rigidity of the scaffold was essential, these parameters had no influence for clusters with C9 chains: all of them showed rather good relative affinity enhancements per inhibitory epitopes between 70 and 160 highlighting the sound combination of the calix[8]arene core and the long alkyl arms. Preliminary docking studies were performed to get insights into the preferred binding modes.

## 1. Introduction

Over the last 30 years a growing interest has been directed towards the molecular recognition of druggable biomolecular targets with calixarene derivatives [1,2,3,4,5,6,7]. This is due to the synthetic and conformational versatility of calixarenes [8,9,10] coupled with their ability to establish multiple interactions with biomolecules [1,2,3,4,5,6,7], which make them a class of compounds suitable for potential applications in the field of biopharmaceutical sciences. Several systems in biology interact through multiple simultaneous molecular contacts, and this concept has been adopted as a new strategy for the design and development of drug candidates. Following this approach, Hamilton and co-workers introduced at the upper rim of a conformationally-blocked, cone-shaped calix[4]arene scaffold four peptide functions which were able to establish multiple interactions with a PDGF growth factor, thus giving rise to antiangiogenic activity in vivo [11,12,13]. In another work, docking studies suggested that multiple H-bond and hydrophobic interactions play a crucial role in the interaction between a *p*-acetamidocalix[4]arene derivative and the protein disulfide isomerase (PDI), which is highly expressed in cancer cell types, including lung, brain, ovarian, melanoma, and prostate [14]. In a further work, in vitro studies [15] showed that conformationally-blocked calix[4]arene derivatives bearing aromatic naphthyl, pyrenyl, and aryl groups at the upper rim were able to inhibit HDAC enzymes, while docking calculations suggested that multiple hydrophobic interactions between the aromatic arms and the hydrophobic pockets close to the active site of the enzymes were essential for the stabilization of the HDAC-calixarene complex. Sansone and Fieschi also used calix[4]arene and calix[6]arene grafted with mannose residues to prevent binding of high mannose structures to the DC-SIGN receptor and thus potentially prevent HIV entry into immature dendritic cells [16]. In another example, Geraci and Spadaro showed that a calix[8]arene carrying eight antigens induced an improved antibody production in mice beyond the additive affect compared to its calix[4]arene counterpart [17]. Recently, glycosidase inhibitors have gained considerable clinical relevance [18,19,20,21] and several glycomimetics such as zanamivir^®^, miglustat^®^, migalastat^®^, voglibose^®^, and acarbose^®^ reached the market. The selective hydrolysis of glycosidic bonds in carbohydrates and glycoconjugates is indeed involved in a plethora of key cellular processes in biological systems [22,23,24,25], and thus targeting glycosidases holds great potential for the treatment of many diseases such as type 2 diabetes [25,26,27], viral infection [21,27,28,29,30,31], tumor metastasis [21,27,32,33], or medical diagnosis [34].

Ten years ago, a breakthrough in the field of glycosidase inhibition was provided by the discovery of the large inhibitory multivalent effect for iminosugar-based multivalent clusters [35,36,37,38,39,40,41,42,43]. The simultaneous non-covalent interactions between several inhibitory epitopes (inhitopes) and one or several glycosidases provides higher specificity, thermodynamic, and kinetic stability compared to one single interaction and can be responsible for considerable affinity enhancements of up to 4700 per inhitope for the best multivalent effect obtained to date with compound **1e** [44].

**Figure 1:**
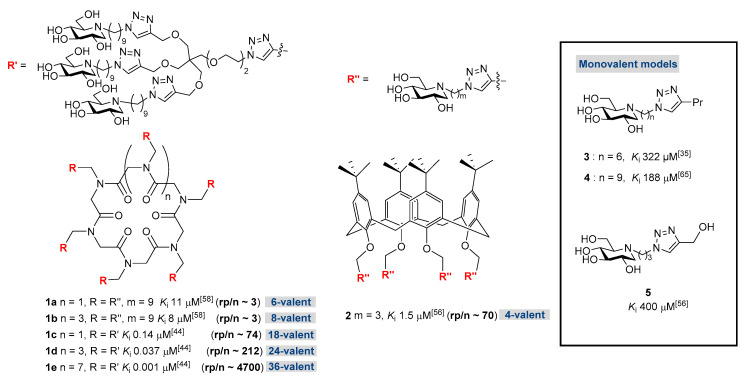
Structure and inhibition constant against Jack Bean α-mannosidase of multivalent clusters and their monovalent references.

The rapid development of this field after the discovery of the first strong multivalent effect [35], was mainly based on structure–activity relationships established from the screening of libraries of multivalent glycomimetics [37,38,39,40,41,42]. Different physical techniques allowed for some rationalization of the observed effects [44,45,46,47,48,49,50,51]. More recently, the acquisition and solving of X-ray structures of two complexes between enzymes and a multimeric inhibitor allowed to draw explanations for the strongest inhibitory multivalent effect obtained to date with cluster **1e** [50], or for an exceptional selectivity [49], and opened the way for a new area where rational design of multivalent inhibitors will be possible. Moreover, multivalent inhibitors have demonstrated their therapeutic potential and have proven to be able to cross membranes by in vitro [52,53] and in vivo [54] experiments. In this project, we wanted to build new multivalent constructs by associating the advantages of calixarene scaffolds with those of 1-deoxynojirimycin (DNJ), the inhitope that has led to the best inhibitory multivalent effect reported so far [44]. DNJ is indeed a moderate and non-selective glycosidase inhibitor that has a high potential for affinity improvement through multivalency [55]. The nature of the scaffold as well as the spatial distribution of the ligands is crucial for the modulation of the inhibitory activity and selectivity. At this regard, calix[n]arenes are particularly suitable as scaffolds for the synthesis of multivalent ligands, thanks to their variable number of reactive positions for inhitope attaching and thanks to the possibility of modulating their spatial orientation. Relatively few examples of calixarene-based iminosugar clusters have been synthesized so far. Tetravalent calix[4]arenes, either fixed in the 1,3-alternate [56] or in the cone structure, bearing four DNJ units at the upper [57] or lower [56,57] rim have been reported independently by Gouin and Marra, but showed modest multivalent effects for the inhibition of JBα-man except for rigid cluster **2** having an affinity enhancement per iminosugar of 70 and inducing aggregation of the enzyme. In all those clusters, the linkers between the inhitope and the scaffold were short— three to five methylene groups in total on both sides of the triazole—and the valency was only four. Previous studies indeed showed that longer linkers had a positive impact on the inhibitory multivalent effect [37,42]. In parallel, clusters **1a,b** [58] of relatively low valency (6 to 8) and based on flexible cyclopeptoid scaffolds also lead to weak multivalent effects. Tripling the initial valency of these scaffolds by the grafting of trivalent azido-armed dendrons dramatically increased the multivalent effect by one to two orders of magnitude (see compounds **1a–e** in Figure 1) [44].

**Figure 2:**
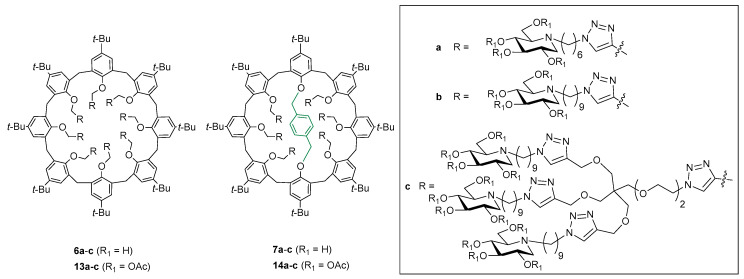
Calix[8]arene based multivalent clusters **6a**–**c** and **7a**–**c**. For a full picture of those compounds see SI Figures S1–S6.

Prompted by these considerations, we decided to synthesize a small library of DNJ-clusters based on the larger calix[8]arene macrocycle as scaffold, which can ensure a higher valency, with up to eight alkynes on the lower rim. A second more rigid 1,5-xylylene bridged [59] calix[8]arene scaffold was also used to probe valency and flexibility modulation. In order to evaluate further the influence of the chain length, but also valency and cluster global size, we grafted C6- and C9- alkylated DNJ heads and also a trivalent DNJ-dendron (Figure 2). The inhibition power of the different clusters has been determined on Jack Bean α-mannosidase (JBα-man) as a model enzyme which is the most sensitive to multivalent presentation known so far. JBα-man is indeed a commercially available source of GH 38 mannosidase and shares 29% of identity with a significant E-value of 2.10^−95^ with human Golgi α-mannosidase II as found by a Blast search with full sequence of JBα-man and lumenal domain of homo sapiens Golgi α-mannosidase II. The latter enzyme is a key member of the early steps of *N*-glycans trimming and has not been crystallized so far. Aberrant *N*-glycans at the tumor surface are associated with cancer growth and metastasis [60]. The inhibitor of lysosomal and Golgi α-mannosidase II (-)-swainsonine reached phase II in a clinical trial, highlighting the potential of α-mannosidase inhibitors [32].

## 2. Results and Discussion

### 2.1. Chemistry

For the sake of efficiency, the synthesis of multivalent clusters usually relies on click reactions as defined by Sharpless [61]. The coupling of calix[4]arenes to get different neoglycoconjugates by amide coupling [62], nitrone cycloaddition [63], or photoinduced radical thiol-ene coupling [64] has already been used besides Cu(I)-catalyzed azide-alkyne cycloaddition (CuAAC) [56,57]. In our case, the general strategy towards targets **6** and **7** (Figure 2) differing by the nature of their central calixarene core and their DNJ coating, was to graft azide-armed DNJ ligands [65] or an azido-trivalent dendron [66] on calix[8]arene scaffolds by Cu(I)-catalyzed azide-alkyne cycloaddition (CuAAC).

**Scheme 1:**
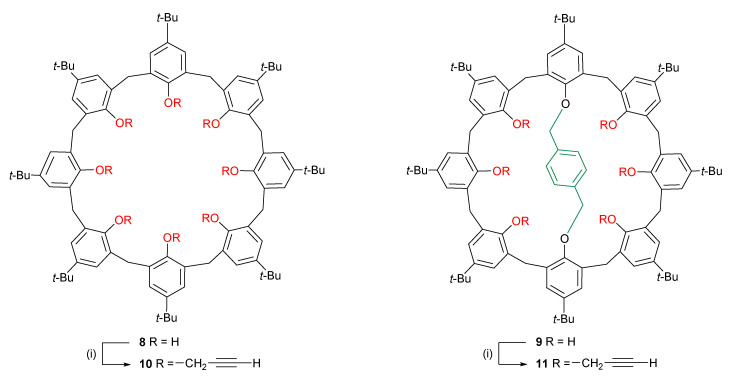
Preparation of scaffolds **10** and **11**. (i) Cs_2_CO_3_, acetone, reflux (**10**: 76%; **11**: 57%).

**Scheme 2:**
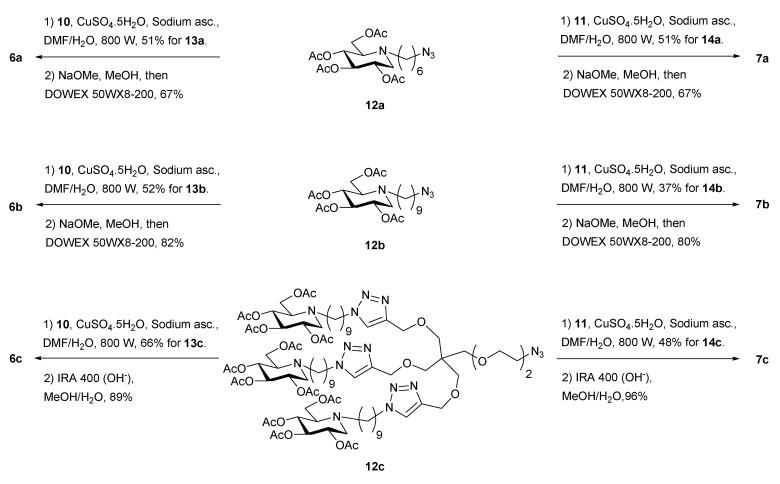
Synthesis of multivalent clusters **6** and **7**.

For this purpose, full propargylation of *p-tert*-butylcalix[8]arene **8** [67] and the 1,5-*p*-xylylene-bridged *tert*-butylcalix[8]arene **9** [59] with propargyl bromide were performed under classical conditions [68] in the presence of Cs_2_CO_3_ as a base in acetone and conducted to propargylated calix[8]arenes **10** and **11** (Scheme 1). To evaluate the influence of the spacer length on the inhibitory activity, two different peracetylated azide-armed DNJ iminosugars **12a** [45] and **12b** [65] bearing, respectively, 6- or 9-carbon alkyl spacers have been grafted by CuAAC onto scaffolds **10** and **11** (Figure 2, Scheme 2). The click reactions were performed under classical conditions using copper sulfate and sodium ascorbate under microwave irradiation to give the corresponding peracetylated clusters **13a**,**b** and **14a**,**b** (Figure 2). Copper salts were removed by filtration over a small pad of silica using a solution of CH_3_CN/AcOEt/(30%) NH_4_OH before column chromatography, a procedure that previously showed its efficiency as proven by ICP-AES analysis of copper traces in final clusters [44]. Derivatives **13a**,**b** and **14a**,**b** were characterized by 1D and 2D NMR spectroscopy and ESI(+) MS (see SI).

The *C*_8_ symmetry of compound **13a** is nicely shown in the ^1^H NMR spectrum (Figure S12) with only one singlet at *δ* = 0.89 ppm for all *t*-Bu groups, one singlet for the aromatic hydrogen atoms of the calix[8]arene skeleton at *δ* = 6.80 ppm and a singlet attributable to the triazole ring hydrogen atom at *δ* = 7.78 ppm. Similar spectra are found for the related **13b** compound differing only from the linker length. The **13a**,**b** spectra are averaged spectra of fast interconverting conformations in the NMR time scale. The interconversion is possible even with *t*-butyl groups because of the larger size of the calix[8]arene macrocycle compared to the calix[4]arene one [69].

Due to the presence of the 1,5-(*p*-xylylene) intramolecular bridge, derivative **14b** has an overall *C*_2_ symmetry but shows characteristics of a *C*_2v_ symmetry locally at the calix[8]arene core, with two perpendicular vertical symmetry planes and a C_2_ axis in their intersection, as reported previously with a derivative of 1,5-*m*-xylene-diyl bridged *tert*-butylcalix[8]arene (see SI Figure S38) [70]. Remarkably, its ^1^H NMR spectrum shows a thin singlet at *δ* = 3.34 ppm for the diethereal methylene of the intramolecular bridge showing nicely their equivalence due to the local *C*_2v_ symmetry. The other methylene protons of the ring appear as two AX systems at 3.43/4.78 ppm (*J* = 14.2 Hz); and 3.51/4.39 ppm (*J* = 16.6 Hz), respectively, showing the rigidity and cone shape of the bridged calix[8]arene. Similarly, the *t*-butyl groups appear as three singlets at 0.41, 1.18, and 1.39 ppm. Two singlets attributed to triazole protons, one arising from the overlapped aromatics at 7.26 and the other one at 7.78 ppm are integrated in a 2:1 ratio. In addition, the presence of only five types of aromatic integrating for 4H as singlets at 5.84, 6.6, 6.96, 7.24, and 7.26 ppm further proves the symmetry. The DNJ heads which are further away from the core appear totally equivalent for all different arms with single signals for all the DNJ carbons. The 2D NMR spectra indeed allowed the full attribution of the ^1^H and ^13^C NMR spectra. Remarkably, the closely related compound **14a** showed a loss of symmetry as seen by the non-equivalent aromatics of half the core and a more complex pattern of methylene protons of the rim. This may be due to the shorter linkers which bring closer the chiral DNJ heads, abolishing the apparent local *C*_2v_ symmetry observed for **14b**. The ^1^H NMR of cluster **14a** thus exhibited exclusively a *C_2_* symmetry showing two singlets attributable to triazole H-atoms at 7.22 and 7.77 ppm and 4 singlets (18 H each) attributable to the *t*-butyl groups at 0.40, 1.18, 1.19, and 1.42 ppm. In addition, two sets of signals for the hydrogen on the C1-position of the iminosugar units at 3.10 and 3.18 ppm integrate for four and two protons, respectively. These two different types of protons showed correlation with the corresponding geminal partners at *δ* = 2.26–2.30 (overlapped). The singlet at *δ* = 3.32 ppm was attributed to the aliphatic protons of the intramolecular bridge, while the corresponding aromatic protons were found at *δ* = 5.82 ppm. The signals of the aromatic protons of the calix[8]arene core were found at *δ* = 6.58, 6.61, 6.95, 6.98, and 7.22–7.28 ppm (overlapped), while the hydrogen atoms on the triazole rings were found at *δ* = 7.22–7.28 (overlapped with the aromatic signals) and 7.77 ppm.

Derivatives **13a**,**b** and **14a**,**b** have a valency of 8 and 6, respectively. In order to increase the valency of clusters by a factor of three, we adopted the strategy relying on the grafting of clickable trivalent dendron **12c** [66], allowing the fast and efficient synthesis of iminosugar clusters with higher valencies. In this way, propargylated calix[8]arene **10** and **11** were reacted by CuAAc with dendron **12c** to afford the 24-valent cluster **13c** and the 18-valent cluster **14c** in 66% and 48% yields, respectively. ^1^H NMR analysis of compound **13c** shows again the *C*_8_ symmetry with well resolved signals of the iminosugar part, and single singlet signals for *t*-butyl groups, aromatic protons, and methylene groups of the core. Those singlets are, however, broader than for compounds **13a** and **13b**, suggesting a slower interconversion on the NMR time scale. ^13^C NMR also shows well resolved signals for the dendron part, but the calix[8]arene core is less resolved even at a higher number of scans due to the dilution of signals among the more intense ones of the dendritic part in addition to the slower interconversion. The *t*-butyl signal appears at 31.1 ppm as a broad signal and at 35.2 ppm for its quaternary center. The core methylene at 29.8 ppm, the aromatic C–H at 124.6 ppm, and the quaternary carbon at 144.2 ppm are also clearly visible as single signals. The ESI-MS of several ions of a multiply charged cluster further confirm the grafting of all positions of the scaffold.

Careful analysis of NMR spectra of compound **14c** indicates the presence of mixture of atropoisomers. As for **14a**,**b**, all iminosugars are equivalent in NMR and very well resolved as they are far from the core center. However, unlike the singlet observed for the bridge’s aromatic for compounds **14a** and **14b** at 5.8 pm, a broad peak at 5.78 ppm and a smaller one at 5.85 ppm integrate together for four protons. In a similar way, the *t*-butyl of the aromatic linked to the bridge appears as a major broad singlet at 0.37 and several other ones. Triazole protons of the dendrons appear as two singlets integrating for nine protons and the triazoles appear close to the calixarene core as several singlets. The characteristic methylenic protons of the core are unfortunately hidden by protons of the oligoethylene part of the dendron. The formation of atropoisomers for this compound can be explained by the fact that its precursor **11** undergoes conformational interconversion because the propargyl groups are not bulky enough to prevent it and are in accordance with the previously reported isolation of atropisomeric derivatives of **9** [71]. During the course of the CuAAC reaction, different blocked conformations arose by progressive grafting of the six large dendrons randomly. The result is a mixture of atropoisomers of the same valency that would be difficult to isolate. Full deprotection of clusters **13a**,**b** and **14a**,**b** into **6a**,**b** and **7a**,**b**, respectively, was achieved by treatment with NaOMe in dry methanol followed by protonation of the alcohols using Dowex 50WX8-200 (H^+^) ion-exchange resin, filtration and solvent and by-products evaporation. Multivalent clusters of higher valency **13c** and **14c** were deprotected with Amberlite IRA 400 (OH^−^) resin allowing directly fully deprotected clean compounds **6c** and **7c** in quantitative yields (Scheme 2). Derivatives **6** and **7** were characterized by 1D and 2D NMR spectroscopy and ESI(+) MS. Clusters **6** are consistent with a *C*_8_ symmetry as their precursors, even if **6c** has broader signals for the calix[8]arene core due to slower conformational inversion. Clusters **7a,b** derived from the hexa-valent scaffold are nicely resolved with a *C_2_* symmetry, but the dendritic **7c** is obtained as an atropoisomer mixture as observed for their precursors. We decided to keep the mixture for final evaluation of inhibitory activity, being aware that only the valency effect and not the spatial arrangement would be taken into discussion.

### 2.2. Biological Assays

The inhibition potency of compounds **6** and **7** was then assayed against JBα-man. All clusters of small size **6a**,**b** and **7a**,**b**, directly derived from alkyl-DNJ were competitive inhibitors of this enzyme (Table 1, entry 3–6, Figures S83, S84 and S88, S89). As observed in a previous study with cyclodextrin-based clusters [65], the three methylene lengthening of the alkyl chain improves the affinity enhancement per inhibitory head (*rp/n*) of at least one order of magnitude (Table 1, entries 3 and 4, entries 5 and 6).

For the bridged calix[8]arene scaffold, the *rp/n* is improved by a factor of 10 (Table 1 entries 3 and 4), whereas a larger gain of 150 in *rp/n* is observed for the flexible calix[8]arene scaffold (Table 1 entries 5 and 6). Remarkably, the simple extension of the alkyl chain by three CH_2_ (from hexyl to nonyl) is thus sufficient to promote a 250-fold increase of inhibitory activity. This effect might be due to hydrophobic interactions between the chain and lipophilic glycine residues G788, G792, and G790 at the entry of the pocket as shown from the X-ray structure of compound **1e** bound to JB α-man (Figure S7). The three supplementary methylene of the C9 chain indeed allow hydrophobic interaction with G788 which is probably missing with the shorter C6 chain. It is interesting to point out that for clusters with the smaller 6-carbon linker, there is a strong influence of the calixarene scaffold as shown by the one magnitude order difference in the *K_i_* values and the absence of multivalent effect (*rp/n* < 1) for compound **6a** (Table 1, entries 3 and 5). In this case, the more rigid scaffold, with the *C_2_* symmetry, seems decisive. However, for the series with longer 9-carbon linkers, the scaffold rigidity has no impact on the inhibition constants and multivalent effects as shown by the comparison of entries 4 and 6 and 7 and 8 (Table 1). Interestingly, it seems that the higher degree of freedom obtained by the addition of three CH_2_ in the hexyl chain is sufficient to screen the mechanical strains due to some more rigid scaffold.

The jump in valency and cluster overall size by grafting trivalent dendron **12c** has more impact on the inhibition and multivalent effect. Clusters **6c** and **7c** showed different inhibitory profiles. While cluster **7c** has a mixed inhibition mode in the nanomolar range, **6c** behavior was more difficult to analyze. Dixon plots showed with a good repeatability a strong nonlinearity (Figure S85). Moreover, double-reciprocal Lineweaver–Burk plots were suggesting a mixed inhibition; however, the respective secondary curves (slope or y-axis intercept as a function of inhibitor concentration) were non-linear (see Figure S86). The shape of those secondary curves did not correspond to hyperbolic partial mixed-type inhibition [72] and its assessment by the graphical method developed by Baici [73] rejected this inhibition modality. A possible slow-binding was searched by continuously following product formation over time in a longer time-scale with a decreased amount of enzyme and the perfect linearity observed showed the absence of a slow binding event. Finally, the competitive tight binding model was tested by fitting a Morrison equation [74,75] for fast tight binding competitive inhibition to the mean values of triplicate experiments. The good matching of the equation to our data gave us the inhibition constant of compound **6c** (*K_i_* = 50 ± 12 nM). The equations and protocol are described in our previous paper where we found a tight binding inhibition pattern for tri- tetra- and tetradecavalent clusters bearing inhibitors of higher affinity [55]. In this latter study, multimerisation of an enzyme-matching strong binding inhitope (mannoimidazole), whose monovalent inhibition was already in the nanomolar range (*K*_i_ 110 nM), led to a tight-binding inhibition with only three or four heads, whereas multimerization of a less good inhibitor (in the low micromolar range with *K_i_* monovalent at 2.2 µM) could lead to tight binding only with a higher valency (14-valent). It is remarkable that a tight-binding behavior against the mannosidase is observed here for a cluster based on the low mismatching *gluco* inhibitor DNJ, which has a monovalent inhibition 100 times weaker, in the hundreds of the micromolar range. Presentation of 24 copies of DNJ allows us to reach an overall kinetic behavior that is attributed to high affinity inhibitors. The competitive tight binding behavior of cluster **6c** is in agreement with the fixation of the DNJ heads into the active sites, as it has been observed in the X-ray structure of compound **1e** with JB α-man [50]. Surprisingly, cluster **7c**, is able to bind both the enzyme and the enzyme:substrate complex, but the latter is less favored with its higher uncompetitive component (*K_i_’* at 0.213 µM). This result, however, suggests that an access for the substrate remains when **7c** is bound to the enzyme.

From a general point of view, the two larger clusters (in size and number of ligands) **6c** and **7c** show the best multivalent effects of the series, with an affinity increase per inhitope over a hundred, in the same range as those obtained with cyclopeptoid-based clusters of identical valencies, 24-valent DNJ **1d** and 18-valent DNJ **1c**, respectively (Figure 1) [44]. Due to conformational interconversion of the *E*/*Z* tertiary amide bonds, cyclopeptoids are known as a conformationally flexible class of scaffolds. The cyclopeptoid platforms are thus less rigid than the *tert*-butylcalix[8]arene scaffold, which itself is less rigid than its 1,5-xylylene-bridged analogue. The fact that close multivalent effects are found for dendron-derived clusters with three platforms of different rigidity further proves that the central core flexibility is not decisive for clusters constructed on trivalent dendron **12c**. Those clusters are anyway very flexible due to the alkyl and oligoethylene chains and may allow dynamic reversible interactions with the enzymes, sometimes through a sandwich cross-linking complex as soon as their global size is large enough to link two JBα-man enzymes [44,50].

In contrast to cyclopeptoid-based clusters whose size is growing together with valency, the two calix[8]arene scaffolds of this study have the same size. Remarkably, all evaluated clusters with C9 alkyl chains show good multivalent effects (with *rp/n* values varying from 70 to 160) even for multimeric iminosugars of lower valencies (6-valent DNJ **7b** and 8-valent DNJ **6b**, Table 1). This is fairly good considering the fact that for the smaller cyclopeptoids **1a,b** bearing also 6 and 8 DNJ units and the same C9 alkyl chains, the *rp/n* was of only 3 (Figure 1) [58]. The sole difference between pairs **1a**/**7b** and **1b**/**6b** being the scaffold, the gain in *rp/n* can be attributed to either the different ring size, different inhitopes local density or by a possible role of the aromatic core.

### 2.3. Docking Studies

**Figure 3:**
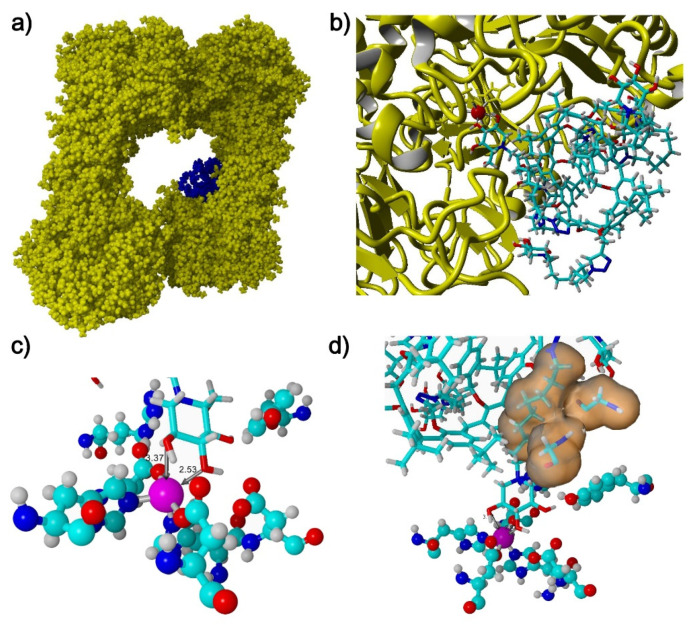
Docking experiments. (**a**,**b**) The 3D model of the binding mode of **7b** with JBα-man. In (**a**) the ligand **7b** is given in blue while the protein is colored in yellow. (**b**) Magnification of the interaction with ligand **7b.** (**c**) Detailed view of the octahedral coordination of Zn ion (in magenta) in enzyme pocket: the coordination of Zn ion with the oxygen atoms of the iminosugar is highlighted. (**d**) Detailed view of the hydrophobic interactions between the lipophilic alkyl chain of the Zn-coordinated DNJ of cluster **7b** with G788 and G790.

**Figure 4:**
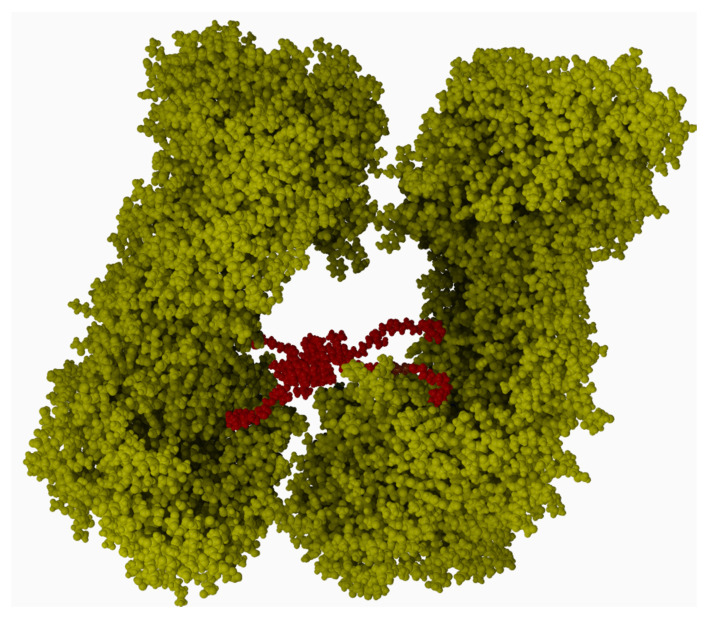
Docking experiments. The 3D model of the octopus-like binding mode of dendritic cluster **6c** to JBα-man dimer. The calculation was performed on a simplified cluster model bearing four DNJ heads in order to overcome computing limitations found during the docking calculation with the complete 24-valent cluster **6c.**

**Figure 5:**
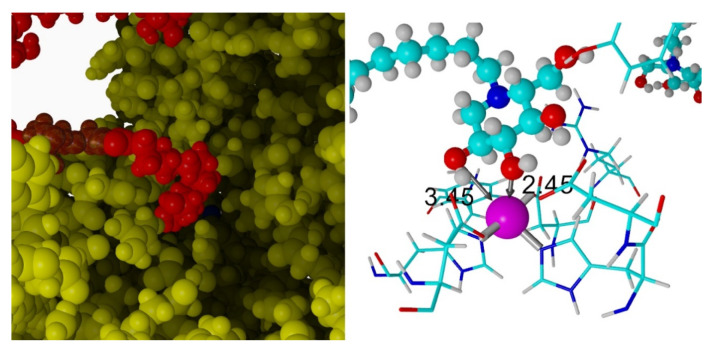
Docking experiments. (**Left**) Detail of the threading of a DNJ head (in red) of **6c** with JBα-man (in yellow). (**Right**) Octahedral coordination of Zn ion (in magenta) in enzyme pocket: the coordination of Zn ion with the oxygen atoms of the iminosugar is highlighted.

In order to rationalize the biological data reported in Table 1 and to gain deeper insights into the possible interaction mode of calix[8]arene-based multivalent clusters with JBα-man, preliminary molecular docking studies were performed (Figure 3, Figure 4 and Figure 5 and Figures S91, S92). It is noteworthy that docking approaches have been recently reported for small calixarenes [15,76,77,78,79]. The multivalent cluster structures were optimized by molecular mechanics calculations and molecular dynamics simulations in a box of water molecules using the YASARA program [80] and AMBER force field [81,82,83,84]. Regarding the protein JBα-man, the previously reported X-ray structure [50] (Protein Data Bank, PDB entry 6B9O) was used as the starting structure for docking studies. As known, the crystallographic asymmetric unit contains one JBα-man protein composed of two LH heterodimers, each constituted by two distinct chains, L and H, to give a symmetrical (LH)_2_ complex (C_2_ axis). The tetrameric structure of the protein shows four open active sites with their zinc ion in the H-chain, turned toward a cavity located at the center of the 2x(LH)_2_ complex composed by the four LH heterodimers. Several complementary techniques showed that this large cavity accommodates large multivalent inhibitors (e.g., **1e**) [44,50]. In solution, the apoprotein JBα-man is mainly found as (LH)_2_, but the 2x(LH)_2_ complex is also observed as a minor species as shown by analytical ultracentrifugation sedimentation velocity (AUC-SV) experiments [44]. While starting the docking studies with the 2x(LH)_2_ complex, the main objectives were to explore all possible binding sites and find the best one and see if some clusters are large enough to be able to bind several sites simultaneously.

The docking studies showed that 6-valent cluster **7b** binds only one active site and one DNJ head occupies the active site pocket (Figure 3a,b), chelating the zinc atom as in the X-ray structure of compound **1e**. Moreover, the hydrophobic chain of the Zn-coordinated DNJ of cluster **7b** establishes hydrophobic interactions with amino acids G788 and G790 (Figure 3d), in accordance with electron density observed for the flexible chain near those two glycine residues in X-ray structure of **1e** in complex with JBα-man [50].

Furthermore, the zinc atom adopts an octahedral coordination (Figure 3c) with oxygen atoms (O2 and O3) of the iminosugar head of **7b** at 3.37 and 2.53 Å, but also two oxygen atoms of the side-chains of Asp145 and Asp25, and two histidine nitrogen atoms (His23 and His386).

Docking studies with derivative **6a** (SI Figure S91), bearing a shorter spacer than **6b**, indicated that hydrophobic interaction with G790 is missing compared to the longer C9 chain confirming the relevance of the spacer length. 

For the huge dendritic cluster **6c,** restrictions on the total atom number prevented the completion of the docking calculation. This problem was overcome by using a simplified cluster model bearing four DNJ heads. Interestingly, starting from an elongated geometry, we found that the global size of this simplified model of cluster **6c** allows it to coordinate two zinc ions in distal binding sites of two enzymes (Figure 4). The cluster, located at the center of the enzyme dimer, adopts an octopus-like structure, cross-linking two distal binding sites. Analogous to what was observed for derivatives **6a** and **7b**, the zinc atom in the active site presents similar octahedral coordination with O2 and O3 of the iminosugar head of **6c** at 2.45 and 3.45 Å (Figure 5), two oxygen atoms from Asp145 and Asp25, and two nitrogens from His23 and His386. Lipophilic interactions were also highlighted in this case, between the two residues of G788, G790, and the aliphatic chain of the Zn-coordinated DNJ of cluster **6c**.

The overall results of the docking studies indicate that clusters **6a**, **6b**, and **7b** bind only one active site at the same time. Their global size is indeed too short to cross-link two enzymes and the affinity enhancement observed may thus be only due to the bind-and-recapture effect. One supplementary lipophilic interaction between longer chains and glycine residues might be involved in the superiority of the C9 arms compared to the C6 ones. The cross linking of two enzymes (Zn-Zn distance ~60–70 A) observed with the longer dendron-elongated calix[8]arene cluster **6c** shows that stabilization of the 2x(LH)_2_ complex is theoretically possible. This result could be extrapolated to cluster **7c** which displays a similar overall size (same ring size and same flexible dendron length). Chelation of two more proximal sites (Zn-Zn distance ~40 A) of the same enzyme was not observed for **7c** here but is not excluded with this cluster size. Even if those mechanisms are possible, they might not occur so frequently for **7c** since the *rp/n* of compounds **6b**, **7b** and **6c**, **7c** are overall in the same range and one magnitude order below the one of the best multivalent inhibitor reported so far, 36-valent cluster **1e**.

## 3. Materials and Methods

### 3.1. Chemistry

**General Information:** All chemicals were reagent grade and were used without further purification. Tetrahydrofuran was dried by heating under reflux over sodium wire in the presence of benzophenone as an indicator while dimethylformamide was dried by activated 3 Å molecular sieves. When necessary, the compounds were dried in vacuum over CaCl_2_. Reactions were monitored by Merck KGaA TLC silica gel plates (0.25 mm)( Kenilworth, NJ, USA) and visualized by 254 nm UV light, or by spraying with H_2_SO_4_-Ce(SO_4_)_2_. The derivatives **8** [67], **12a** [45], **12b** [65], and **12c** [66] have been synthesized according to literature procedures. Proton ^1^H and carbon ^13^C nuclear magnetic resonance (NMR) experiments were recorded at 298K except when specified on Bruker Avance III HD 400 MHz, 500 MHz, or 600 MHz spectrometer (Billerica, MA, USA). Chemical shifts are reported relative to the residual solvent peak. A COSY spectrum was taken using a relaxation delay of 2 s with 30 scans and 170 increments of 2048 points each. HSQC spectra were performed with gradient selection, sensitivity enhancement, and phase sensitive mode using Echo/Antiecho-TPPI procedure. Optical rotations were measured at 589 nm (sodium lamp) and 20 °C on either a Perkin-Elmer 341 polarimeter (Waltham, MA, USA) or an Anton Paar MCP 200 polarimeter(Graz, Austria) with a path length of 1 dm. Infrared (IR) spectra were recorded neat on a Perkin–Elmer Spectrum Two FT-IR spectrometer. High-resolution (HRMS) electrospray ionization-time-of-flight (ESI-TOF) mass spectra were recorded on a Bruker micrOTOF^®^ mass spectrometer.

**Synthesis of derivatives 10** and **11:** Cs_2_CO_3_ (1.25 equiv for each OH group) as added to a suspension of the appropriate calix[8]arene derivative **8** or **9** (1.0 g) in acetone (100 mL). The reaction mixture was stirred at reflux for 2 h. Then the system was cooled at room temperature and propargyl bromide (2.50 equiv for each OH group) was added. The reaction mixture was then stirred at reflux for 20 h, then it was cooled at room temperature and the solvent was removed under reduced pressure. An aqueous 1N solution of HCl (150 mL) was added to the reaction mixture and the suspension was stirred for 15 min. The aqueous phase was extracted and washed with CH_2_Cl_2_ (3 × 70 mL) and the organic phases were collected, dried over Na_2_SO_4_, filtered, and the solvent was removed under reduced pressure. The crude product was dissolved in a small amount of CH_2_Cl_2_ and then MeOH was added. The precipitated was filtered, affording the pure product as a white solid.

**Compound 10:** (76% yield); ESI(+) MS: *m*/*z* = 1603.1 [M + H]^+^, 1624.9 [M + Na]^+^; ^1^H NMR (400 MHz, CDCl_3_, 298 K): *δ* 1.11 (s, *t*Bu, 72H), 2.30 (br t, 8H), 4.10 (s, 16H), 4.21 (d, *J =* 2.1 Hz, 16H), 6.95 (s, 16H) ppm; ^13^C NMR (100 MHz, CDCl_3_, 298K): *δ* 31.2, 31.5, 34.4, 60.9, 75.4, 79.7, 126.3, 133.4, 146.9, 153.1 ppm; Anal. calcd for C_112_H_128_O_8_: C, 83.96; H, 8.05; found C, 83.01; H, 8.16. **Compound 11:** (57% yield); ESI(+) MS: *m/z* = 1628.7 [M+H]^+^, 1647.9 [M+Na]^+^, 1667.9 [M+K]^+^; ^1^H NMR (600 MHz, CDCl_3_, 298K): *δ* 0.55 (s, 18H), 1.17 (s, 36H), 1.38 (s, 18H), 2.46 (br t, 4H), 2.56 (br t, 2H), 3.56 (s, 4H), 4.10 (s, 8H), 4.21 (s, 8H), 4.57 (s, 8H), 4.74 (s, 4H), 5.95 (s, 4H), 6.70 (s, 4H), 6.91 (s, 4H), 7.24 (br d, *J* = 2.2 Hz, 4H), 7.27 (s, 4H) ppm; ^13^C NMR (150 MHz, CDCl_3_, 298K): δ 31.0, 31.1, 31.6, 31.8, 34.0, 34.5, 60.6, 61.4, 75.2, 75.6, 80.0, 80.4, 124.6, 124.7, 126.3, 127.2, 128.3, 129.0, 132.4, 133.1, 133.6, 134.9, 136.43, 146.2, 146.4, 146.5, 151.5, 152.6, 154.4 ppm. Anal. calcd for C_114_H_130_O_8_: C, 84.09; H, 8.05; found C, 83.10; H, 8.14.

**General procedure for the CuAAC with 12a and 12b:** The calix[8]arene derivative **10** or **11** (10–15 mg) and the appropriate azide-armed DNJ iminosugar **12a** or **12b** (1.2 equiv for each alkyne unit) were dissolved in DMF (1.0 mL) in an ACE pressure tube. Then, a solution of Cu_2_SO_4_.5H_2_O (0.1 equiv. for each alkyne unit) and sodium ascorbate (0.2 equiv. for each alkyne unit) in water (0.25 mL) was added. The reaction mixture was heated under microwave irradiation for 20–30 min, using a CEM Corporation Discover LabMate system with the temperature controller. The mixture was concentrated, and traces of copper salts were removed by filtration through a short pad of silica gel eluting with CH_3_CN/AcOEt/NH_4_OH (9:1:1). The filtrate was concentrated and then purified by flash chromatography on silica gel.

**Compound 13a:** (AcOEt-AcOEt:MeOH 98:2, 51% yield; MS (ESI) *m/z* calcd for C_272_H_384_N_32_Na_2_O_72_ [M + 2Na]^2+^ 2648.3577; found 2648.3351; [α]_D_^28^ = +3.5 (*c* = 1, CHCl_3_); ^1^H NMR (300 MHz, CDCl_3_, 298K): δ 0.89 (s, 72H), 1.25 (br, 32H), 1.39 (br, 16H), 1.77 (br, 16H), 2.00–2.04 (m, 96H), 2.30 (t, *J* = 10.6 Hz, 8H), 2.53 (m, 8H), 2.61 (m, 8H), 2.70 (m, 8H), 3.16 (dd, *J*_1_ = 11.6 Hz, *J*_2_ = 4.9 Hz, 8H), 4.06 (s, 16H), 4.14 (br, 16H), 4.18 (overlapped, 16H), 4.79 (s, 16H), 4.92 (m, 8H), 5.03 (overlapped, 16H), 6.80 (s, 16H), 7.79 (s, 8H) ppm; ^13^C NMR (75 MHz, CDCl_3_, 298K): δ 20.9, 20.9, 21.1, 24.8, 26.6, 26.9, 30.2, 30.5, 31.4, 34.2, 50.2, 51.9, 53.1, 59.6, 61.6, 66.6, 69.6, 69.7, 74.9, 123.9, 126.1, 133.3, 144.1, 146.6, 152.7, 169.9, 170.2, 170.5, 171.0 ppm. Anal. calcd for C_272_H_384_N_32_O_72_: C, 62.18; H, 7.37; found C, 62.07; H, 7.48.

**Compound 13b:** (AcOEt:Petroleum Ether, 8:2, 52% yield); MS (ESI) *m/z* calcd for C_296_H_432_N_32_Na_2_O_72_ [M + 2Na]^2+^ 2816.5455; found 2816.5469; [α]_D_^27^ = +5.0 (*c* = 1, CHCl_3_); ^1^H NMR (600 MHz, CDCl_3_, 298K): *δ* 0.90 (s, 72H), 1.24 (overlapped, 80H), 1.39 (br, 16H), 1.76 (br, 16H), 2.00–2.06 (overlapped, 96H), 2.32 (t, *J* = 10.9 Hz, 8H), 2.55 (m, 8H), 2.62 (br d, *J* = 9.2 Hz, 8H), 2.70 (m, 8H), 3.17 (dd, *J*_1_ = 11.4 Hz, *J*_2_ = 5.1 Hz, 8H), 4.06 (s, 16H), 4.14 (br, 16H), 4.18 (br, 16H), 4.79 (s, 16H), 4.95 (m, 8H), 5.0–5.07 (overlapped, H3+H4, 16H), 6.80 (s, 16H), 7.78 (s, 8H) ppm; ^13^C NMR (150 MHz, CDCl_3_, 298 K): 20.9, 20.9, 21.1, 21.1, 24.8, 26.8, 27.5, 29.3, 29.7, 29.9, 30.6, 31.4, 34.2, 50.3, 52.1, 53.2, 59.7, 61.6, 66.6, 69.7, 69.7, 74.9, 123.9, 126.1, 133.3, 144.2, 146.6, 152.7, 170.0, 170.2, 170.6, 171.1 ppm. Anal. calcd for C_296_H_432_N_32_O_72_: C, 63.59; H, 7.79; found C, 63.50; H, 7.86.

**Compound 14a:** (AcOEt/Petroleum Ether 9:1-AcOEt, 51% yield); MS (ESI) *m/z* calcd for C_234_H_322_N_24_Na_2_O_56_ [M + 2Na]^2+^ 2205.1435; found 2205.1329; [α]_D_^30^ = +4.0 (*c* = 1, CHCl_3_); ^1^H NMR (600 MHz, CDCl_3_, 298K): δ 0.40 (s, 18H), 0.95–1.54 (overlapped, 44H), 1.17 (s, 36H), 1.41 (s, 18H), 1.86 (t, *J* = 7.2 Hz, 12H), 1.98–2.06 (overlapped, 72H), 2.25–2.30 (overlapped, 6H), 2.42–2.62 (overlapped, 14H), 2.69–2.74 (m, 4H), 3.10 (dd, *J*_1_ = 11.5 Hz, *J*_2_ = 5.2 Hz, 4H), 3.18 (dd, *J*_1_ = 11.5 Hz, *J*_2_ = 5.2 Hz, 2H), 3.32 (s, 4H), 3.44–3.48 (overlapped, 4H), 3.53 (m, 4H), 3.86–3.97 (overlapped, 8H), 4.07–4.17 (overlapped, 12H), 4.33 (t, *J* = 7.8 Hz, 4H), 4.36–4.40 (overlapped, 4H), 4.79 (d, *J* = 13.2 Hz, 4H), 4.89–4.96 (overlapped, 6H), 4.97–5.08 (overlapped, 12H), 5.82 (s, 4H), 6.58 (s, 2H), 6.61 (s, 2H), 6.95 (s, 2H), 6.98 (s, 2H), 7.22–7.28 (overlapped, 6H), 7.77 (s, 4H) ppm; ^13^C NMR (150 MHz, CDCl_3_, 298 K): δ 20.9, 21.0, 21.1, 24.4, 25.0, 26.5, 26.7, 26.8, 29.9, 30.3, 30.4, 30.6, 31.1, 31.6, 31.9, 33.9, 34.5, 34.5, 50.1, 50.4, 51.7, 51.9, 51.9, 53.1, 53.2, 59.4, 59.7, 61.5, 61.6, 61.9, 66.2, 67.0, 69.6, 69.7, 74.8, 75.0, 123.1, 123.3, 123.4, 124.0, 124.3, 127.3, 128.7, 129.2, 132.7, 133.4, 134.6, 134.7, 136.6, 144.0, 144.34, 146.5, 146.7, 152.4, 153.8, 169.9, 170.1, 170.3, 170.6, 171.1 ppm. Anal. calcd for C_234_H_322_N_24_O_56_: C, 64.36; H, 7.43; found C, 64.26; H, 7.51.

**Compound 14b:** (AcOEt/Petroleum Ether 72:28–AcOEt, 37% yield); MS (ESI) *m/z* calcd for C_252_H_358_N_24_Na_2_O_56_ [M + 2Na]^2+^ 2331.2844; found 2331.7650; [α]_D_^31^ = +7.5 (*c* = 1, CHCl_3_); ^1^H NMR (600 MHz, CDCl_3_, 298K): δ 0.41 (s, 18H), 1.05–1.30 (overlapped, 60H), 1.18 (s, 36H), 1.39 (s, 18H), 1.54 (br, 8H), 1.86 (br, 4H), 2.00–2.02 (overlapped, 72H), 2.29–2.34 (overlapped, 6H), 2.52–2.57 (overlapped, 6H), 2.61–2.64 (overlapped, 6H), 2.66–2.74 (overlapped, 6H), 3.14–3.20 (overlapped, 6H), 3.34 (s, H18, 4H), 3.43 (d, *J* = 14.4 Hz, 4H), 3.51 (d, *J* = 16.8 Hz, 4H), 3.89–3.99 (m, 8H), 4.10–4.17 (overlapped, 12H), 4.33 (t, *J* = 7.3 Hz, 4H), 4.39 (d, *J* = 16.6 Hz, 4H), 4.78 (d, *J* = 14.2 Hz, 4H), 4.92–4.97 (overlapped, 6H), 5.00–5.08 (overlapped, 24H), 5.84 (s, ArH, 4H), 6.60 (s, 4H), 6.96 (s, 4H), 7.24 (s, 4H), 7,26 (overlapped, 8H), 7.78 (s, 2H) ppm; ^13^C NMR (150 MHz, CDCl_3_, 298 K): δ 20.9, 21.0, 21.1, 21.2, 21.3, 22.9, 24.6, 24.9, 26.7, 26.7, 27.4, 27.5, 29.1, 29.2, 29.6, 29.7, 29.9, 30.1, 30.4, 30.6, 30.8, 31.1, 31.6, 31.8, 32.1, 33.9, 34.5, 50.3, 50.6, 52.0, 52.2, 53.2, 53.2, 59.7, 59.7, 60.6, 61.5, 61.7, 66.2, 67.1, 69.7, 69.7, 74.9, 75.0, 123.1,123.5, 124.0, 124.3, 127.3, 128.6, 129.2, 131.9, 132.8, 133.4, 134.7, 136.7, 144.2, 144.4, 145.9, 146.4, 146.5, 151.3, 152.4, 153.9, 169.9, 170.2, 170.2, 170.6, 171.1, 171.4 ppm. Anal. calcd for C_252_H_358_N_24_O_56_: C, 65.52; H, 7.81; found C, 65.43; H, 7.90.

**General procedure for deacetylation of clusters 13a,b and 14a,b:** The acetylated compound (10–15 mg) was dissolved in dry methanol (0.3–0.5 mL), and then NaOMe (0.45 equiv for each acetyl group) was added. The reaction mixture was stirred at room temperature for 6 h under nitrogen atmosphere. Methanol and water were added to dissolve the white precipitate. Dowex 50WX8-200 resin was added until pH=6–7 was reached. Then the solution was filtered and concentrated to give the pure compound.

**Compound 6a:** 67% yield; MS (ESI) *m*/*z* calcd for C_208_H_320_Na_2_O_40_ [M + 2Na]^2+^ 1676.1887; found 1976.1792; [α]_D_^33^= +13.0 (*c* = 0.067, MeOH); ^1^H NMR (600 MHz, MeOD, 298 K): *δ* 0.96 (s, 72H), 1.26–1.29 (overlapped, 32H), 1.50 (br, 16H), 1.73 (br, 16H), 2.31 (overlapped, 8H), 2.34 (overlapped, 8H), 2.68 (m, 8H), 2.88 (m, 8H), 3.08 (dd, *J*_1_ *=* 11.7 Hz, *J*_2_ = 5.04 Hz, 8H), 3.19 (t, *J* = 9 Hz, 8H), 3.40 (t, *J* = 9.2 Hz, 8H), 3.53 (m, 8H), 3.87 (br, 16H), 4.09 (s, 16H), 4.19 (br, 16H), 4.80 (br s, 16H), 6.89 (s, 16H), 7.96 (s, 8H), 8.51 (s, OH) ppm; ^13^C NMR (150 MHz, MeOD, 298 K): δ 25.1, 27.0, 27.5, 27.9, 28.2, 30.4, 30.5, 30.9, 31.3, 32.1, 33.2, 35.2, 36.6, 51.3, 53.9, 57.2, 58.7, 67.5, 70.2, 71.4, 80.1, 125.9, 131.0, 134.6, 145.2, 147.9, 154.1 ppm. Anal. calcd for C_208_H_320_N_32_O_40_: C, 63.91; H, 8.25; found C, 64.01; H, 8.16.

**Compound 6b:** 82% yield; MS (ESI) *m*/*z* calcd for C_232_H_368_N_32_Na_2_O_40_ [M + 2Na]^2+^ 2144.3765; found 2144.3665; [α]_D_^27^ = −4.0 (*c* = 0.24, MeOH); ^1^H NMR (600 MHz, MeOD, 313 K): *δ* 0.95 (s, 72H), 1.25 (overlapped, 80H), 1.57 (br, 16H), 1.76 (br, 16H), 2.46–2.49 (overlapped, 16H), 2.80 (m, 8H), 3.00 (m, 8H), 3.17 (dd, *J*_1_ = 11.3 Hz, *J*_2_ = 4.56 Hz, 8H), 3.23 (t, *J* = 9.1 Hz, 8H), 3.45 (t, *J* = 9.3 Hz, 8H), 3.57 (m, 8H), 3.87 (dd, *J*_1_ = 11.9 Hz, *J*_2_ = 2.5 Hz, 8H), 3.94 (dd, *J*_1_ = 12.1 Hz, *J*_2_ = 2.0 Hz, 8H), 4.07 (s, 16H), 4.21 (br, 16H), 4.81 (s, 16H), 6.88 (s, 16H), 7.96 (s, 8H), 8.43 (br s, 32H) ppm; ^13^C NMR (600 MHz, MeOD, 313 K): δ 25.0, 27.6, 28.2, 30.0, 30.3, 30.4, 30.5, 30.6, 30.7, 30.8, 31.2, 31.3, 31.9, 33.2, 35.1, 36.5, 49.8, 51.2, 53.9, 54.7, 56.6, 58.0, 67.4, 67.5, 69.6, 70.9, 79.6, 125.8, 127.2, 134.6, 145.2, 147.8, 154.0 ppm. Anal. calcd for C_232_H_368_N_32_O_40_: C, 65.63; H, 8.74; found C, 65.54; H, 8.84.

**Compound 7a:** 67% yield; MS (ESI) *m*/*z* calcd for C_186_H_274_N_24_Na_2_O_32_ [M + 2Na]^2+^ 1701.0168; found 1701.0117; [α]_D_^29^ = −2.0 (*c* = 1, MeOH); ^1^H NMR (600 MHz, MeOD, 298 K): δ 0.44 (s, 18H), 1.23 (s, 36H), 1.24–1.49 (overlapped, 44H), 1.48 (s, 18H), 1.81 (t, *J* = 7.2 Hz, 4H), 2.40–2.52 (overlapped, 12H), 2.68–2.75 (overlapped, 6H), 2.87–2.97 (overlapped, 6H), 3.11–3.16 (overlapped, 6H), 3.20–3.24 (overlapped, 6H), 3.36 (s, 4H), 3.40–3.46 (overlapped, 6H), 3.52–3.58 (overlapped, 6H), 3.60–3.65 (overlapped, 8H), 3.82–3.94 (overlapped, 20H), 4.37–4.42 (overlapped, 8H), 4.89–4.96 (overlapped, 8H), 5.15 (br s, 8H), 5.86 (s, 4H), 6.67 (s, 4H), 7.05 (d, *J* = 7.2 Hz, 4H), 7.36 (s, 4H), 7.39 (s, 4H), 7.42 (s, 4H), 8.28 (s, 2H), 8.44 (br s, OH) ppm; ^13^C NMR (150 MHz, MeOD, 298 K): δ 23.7, 24.5, 24.5, 24.7, 27.2, 27.27, 27.6, 30.3,30.5, 30.6, 30.7, 31.0, 31.1, 31.2, 31.9, 32.0, 32.2, 33.1, 34.9, 35.3, 35.6, 51.3, 51.3, 53.7, 53.7, 56.4, 56.5, 56.7, 57.5, 58.1, 66.7, 67.3, 67.4, 67.5, 69.4, 69.5, 69.7, 70.6, 71.0, 76.2, 79.5, 79.6, 79.7, 125.0, 126.0, 126.1, 128.7, 129.8, 130.3, 133.2, 133.2, 134.1, 134.9, 135.4, 137.7, 145.1, 145.1, 145.3, 147.1, 148.0, 148.1, 152.57, 153.5, 153.5, 154.8 ppm. Anal. calcd for C_186_H_274_N_24_O_32_: C, 66.52; H, 8.22; found C, 66.62; H, 8.12.

**Compound 7b:** 80% yield; MS (ESI) *m*/*z* calcd for C_204_H_310_N_24_Na_2_O_32_ [M + 2Na]^2+^ 1827.1576; found 1827.1540; [α]_D_^29^ = −4.0 (*c* = 1, MeOH); ^1^H NMR (600 MHz, MeOD, 298 K): δ 0.45 (s, 18H), 1.03–1.29 (overlapped, 60H), 1.22 (s, 36H), 1.45 (s, 18H), 1.48–1.56 (overlapped, 20H), 1.83 (m, 4H), 2.35–2.45 (overlapped, 12H), 2.71–2.78 (overlapped, 6H), 2.90–2.96 (overlapped, 6H), 3.09–3.14 (overlapped, 6H), 3.38 (s, 4H), 3.40–3.45 (overlapped, 6H), 3.50–3.56 (overlapped, 14H), 3.85–3.97 (overlapped, 20H), 4.36–4.42 (overlapped, 8H), 4.87–4.92 (overlapped, 8H), 5.11 (d, *J* = 12 Hz, 4H), 5.16 (s, 4H), 5.89 (s, 4H), 6.67 (s, 4H), 7.02 (s, 4H), 7.35 (s, 4H), 7.36 (s, 4H), 7.40 (s, 4H), 8.25 (s, 2H), 8.50 (br s, OH) ppm; ^13^C NMR (150 MHz, MeOD, 298 K): δ 23.7, 25.0, 25.2, 27.4, 27.5, 28.3, 29.8, 29.9, 30.2, 30.4, 30.7, 31.1, 31.3, 31.4, 31.7, 31.9, 32.0, 32.2, 34.8, 35.3, 35.3, 51.4, 53.9, 54.8, 56.9, 57.0, 58.3, 58.6, 66.8, 67.4, 67.5, 69.9, 70.0, 71.2, 71.3, 79.8, 79.9, 125.1, 125.3, 126.0, 128.7, 129.7, 130.3, 133.3, 134.1, 134.8, 135.6, 137.9, 145.2, 147.1, 147.8, 147.9, 152.5, 153.6, 154.9 ppm. Anal. calcd for C_204_H_310_N_24_O_32_: C, 67.86; H, 8.65; found C, 67.97; H, 8.56.

**General Procedure for the CuAAC Reaction with trivalent dendron 12c:** The calix[8]arene derivative **10** or **11** (10–15 mg) and the derivative **12c** (1.2 equiv. for each alkyne unit) were dissolved in DMF (1.0 mL) in an ACE pressure tube. Then, a solution of Cu_2_SO_4_.5H_2_O (0.1 equiv. for each alkyne unit) and sodium ascorbate (0.2 equiv. for each alkyne unit) in water (0.25 mL) was added. The resulting suspension was heated under microwave irradiation at 80 °C for 30 min, then water (10 mL) was added, and the aqueous phase was extracted with EtOAc (3 × 12 mL). The organic phases were combined, dried with Na_2_SO_4_, and concentrated in vacuo. Traces of copper salts were removed by filtration through a short pad of silica gel eluting with CH_3_CN/H_2_O/NH_4_OH (15:0.5:0.5), and the residue was then purified by flash chromatography (SiO_2_; CH_2_Cl_2_/MeOH, 99:1 to 95:5) to give the desired iminosugar.

**Compound 13c:** 66% yield; [α]_D_^20^ = +3.5 (*c* = 1, MeOH); ^1^H NMR (CDCl_3_, 400 MHz): *δ* 7.85 (br s, 8H), 7.57 (s, 24H), 6.8 (br s, 16H), 5.10–4.99 (m, 48H), 4.98–4.91 (m, 24H), 4.81 (br s, 53H), 4.51 (s, 48H), 4.41 (br s, 10H), 4.33–4.27 (t, *J =* 7.4 Hz, 48H), 4.14 (s, 48H), 4.03 (br s, 92H), 3.52–3.36 (m, 96H), 3.21–3.15 (dd, *J*_1_ *=* 11.4, *J*_2_ = 5.0 Hz, 24H), 2.76–2.66 (m, 24H), 2.65–2.60 (m, 24H), 2.59–2.50 (m, 24H), 2.35–2.28 (dd, *J*_1_ *=* 10.9, *J*_2_=10.5 Hz, 24H), 2.06 (s, 72H), 2.02–1.99 (overlapped, 216H), 1.90–1.84 (m, 48H,), 1.46–1.16 (288H), 0.97–0.72 (m, 72H) ppm; ^13^C-NMR (CDCl_3_, 100 MHz): δ 171.0, 170.4, 170.1, 169.9, 145.3, 122.6, 74.9, 70.4–69.8, 69.7, 69.6, 69.4, 65.2, 61.6, 59.7, 53.1, 51.9, 50.4, 50.1, 45.5, 31.3, 30.5, 29.6, 29.2, 27.3, 26.7, 24.9, 21.0, 21.0, 20.9, 20.8 ppm; IR (neat) 1745 cm^−1^; MS (ESI, deconvoluted)**:** *m*/*z* calcd for C_808_H_1249_N_120_O_240_ [M + 10H]^10+^ 1648.4740; found 1648.4710.

**Compound 14c:** 48% yield; [α]_D_^20^ = + 4.5 (*c* = 1, MeOH); ^1^H NMR (CDCl_3_, 400 MHz, 298K): δ 8.11–7.59 (m, 57H), 7.61–7.50 (m, 18H), 7.38–7.16 (m, 8H), 6.96–6.58 (m, 4H), 6.58 (s, 4H), 5.89–5.73 (m, 4H), 5.16–4.99 (m, 48H), 4.99–4.91 (m, 18H), 4.89–4.63 (m, 9H), 4.61–4.44 (m, 42H), 4.36–4.22 (m, 42H), 4.14 (s, 36H), 3.91–3.80 (m, 4H), 3.78–3.63 (m, 8H), 3.54–3.31 (m, 72H), 3.21–3.15 (dd, *J*_1_ = 11.5 and *J*_2_ =4.9 Hz, 18H), 2.76–2.66 (m, 18H), 265–2.60 (m, 18H), 2.59–2.49 (m, 18H), 2.35–2.28 (t, *J* = 10Hz, 18H), 2.09–2.04 (s, 54H), 2.03–2.00 (s, 108H), 2.01–1.98 (s, 54H), 1.93–1.79 (m, 36H), 1.49–1.10 (m, 270H), 0.37 (s, 18H) ppm; ^13^C NMR (CDCl_3_, 75 MHz, 298 K): δ 170.9, 170.4, 170.1, 169.8, 162.6, 145.42, 145.37, 144.2, 122.62, 122.53, 74.9, 69.8, 69.6, 69.5, 68.1, 65.2, 61.7, 59.8, 53.1, 52.0, 51.9, 50.4, 50.4, 45.6, 45.5, 36.6, 34.4, 34.4, 31.8, 31.6, 31.5, 31.1, 30.5, 29.6, 29.2, 27.4, 26.7, 25.8, 25.1, 25.0, 20.97, 20.94, 20.85, 20.80 ppm; MS (ESI, deconvoluted) *m*/*z* calcd for C_636_H_978_N_90_O_182_ [M + 14H]^14+^ 913.8864; found 913.9960.

**General procedure for the deacetylation reaction of 13c and 14c:** Amberlite resin IRA 400 (6*n* g/mmol of substrate; *n* = number of acetate groups) was added to a solution of acetylated iminosugar in a mixture of MeOH/H_2_O (1:1), and the resulting solution was stirred for 4 h by using a rotary evaporator at atmospheric pressure. The resin was then removed by filtration and washed with methanol and water. Solvents were evaporated under reduced pressure to give the desired deprotected iminosugar.

**Compound 6c:** 89% yield; [α]_D_^20^ =−2.5 (*c* = 1, MeOH); ^1^H NMR (MeOD, 400 MHz, 298K): δ 8.22–7.99 (br s, 8H), 7.97–7.82 (s, 24H), 7.19–6.59 (br s, 16H), 4.57–4.42 (m, 64H), 4.41–4.28 (m, 48H), 3.84 (s, 48H), 3.54–3.32 (overlapped, 64H), 3.20–3.07 (m, 24H,), 3.01–2.94 (dd, *J*_1_ = 11.9, *J*_2_ = 5.5 Hz, 24H), 2.83–2.71 (m, 24H), 2.62–2.50 (m, 24H), 2.20–2.12 (dd, *J*_1_ = 11.9, *J*_2_ = 11.5 Hz, 24H), 2.12–2.07 (m, 24H), 1.94–1.79 (m, 48H), 1.55–1.38 (m, 48H), 1.39–1.17 (m, 240H), 0.99–0.79 (m, 72H) ppm; ^13^C-NMR (MeOD, 100 MHz, 298K) *δ* 146.2, 124.9, 80.6, 72.1, 70.8, 67.4, 65.5, 59.6, 57.8, 53.8, 51.4, 32.1, 31.4, 30.6, 30.1, 28.6, 27.6, 25.3 ppm; IR (neat) 3356 (broad strong OH) cm^−1^; MS (ESI, deconvoluted) *m*/*z* calcd for C_616_H_1058_N_120_O_144_ [M + 10H]^10+^ 1244.9054; found 1244.9090.

**Compound 7c**: 96% yield; [α]_D_^20^ =−5.0 (*c* = 1, MeOH/H_2_O 1:1); ^1^H NMR (MeOD, 500 MHz, 298K): δ 8.33 (s, 2H), 7.99–7.85 (several s, 18H), 7.69 (several s, 4H), 7.41–7.20 (m, 8H), 6.96 (s, 4H), 6.80–6.55 (m, 4H), 5.95–5.81 (m, 4H), 5.21–4.98 (m, 12H), 4.64 (m, 4H), 4.54–4.25 (m, 88H), 3.84 (m, 40H), 3.73–3.58 (m, 8H), 3.54–3.22 (m, 120H), 3.13 (t, *J* = 9.0 Hz, 18H), 2.97 (dd, *J* = 11.2 and 5.1 Hz, 18H), 2.82–2.72 (m, 18H), 2.61–2.51 (m, 18H), 2.16 (dd, *J* = 11.1 and 10.0 Hz, 18H), 2.10 (m, 18H), 1.90–1.77 (m, 36H), 1.51–1.19 (m, 270H), 0.43 (s, 18H) ppm; ^13^C-NMR (MeOD, 125 MHz, 20480 scans) *δ* 155.0, 153.5, 152.4, 147.8, 147.5, 146.8, 146.1, 145.3, 145.1, 137.9, 135.8, 135.0, 134.2, 133.6, 133.3, 132.4, 130.4, 129.86, 129.8, 129.5, 128.8, 126.9, 126.8, 125.9, 125.2, 124.9, 80.6, 76.2, 72.0, 70.8, 71.3, 71.2, 70.3, 70.11, 69.99, 69.08, 67.3, 65.51, 65.46, 59.5, 57.8, 53.8, 51.3, 46.53, 46.50, 35.4, 35.3, 34.9, 32.3, 32.2, 32.0, 31.4, 30.6, 30.1, 28.6, 27.5, 25.2) ppm. IR (neat) 3338 (broad strong OH) cm^−1^. MS (ESI) *m*/*z* calcd for C_492_H_830_N_90_O_110_ [M + 10H]^10+^ 976.6409; found 976.6068.

### 3.2. Biological Assays

*p*-nitrophenyl-α-D-mannopyranoside and α-mannosidase (EC 3.2.1.24, from Jack Bean, *Km* = 2.0 mM pH 5.5) were purchased from Sigma Aldrich(St. Louis, MO, USA).

**General procedure for Inhibition assay with basic quench:** Inhibitory potencies were determined spectrophotometrically (Versamax 96-well plate spectrophotometer, Molecular Devices Corporation, Sunnyvale, CA, USA) measuring the residual hydrolytic activities of the mannosidase against *p*-nitrophenyl-α-D-mannopyranoside in the presence and absence of the inhibitor. All kinetics were performed at 25 °C and started by enzyme addition (0.015 U/mL) in a 100 µL assay medium (acetate buffer, 0.2 M, pH = 5) containing substrate (varying concentration from *Km*/8 to 2*Km* value) in presence or absence of various concentrations of inhibitor. After 40–50 min incubation, the reaction was quenched by addition of 1M Na_2_CO_3_ (100 µL). The absorbance of the resulting solution was determined at 405 nm. *K_i_* values were determined in duplicate or triplicate, using the Dixon and Lineweaver–Burk graphical methods [72] with Microsoft Excel 97-03, or using non-linear regression with GraphPad Prism 7 Software [55]. As the inhibitors are only partially soluble in water, they were dissolved in DMSO for concentrated mother solutions and DMSO/buffer for diluted solutions with a final DMSO concentration under 4% in all wells. Previously, the stability of the enzymes in presence of various concentrations of DMSO was controlled and the enzyme activity was unaffected.

**General procedure for inhibition assay measured continuously:** The release of *p*-nitrophenol was measured continuously at 385 nm to determine initial velocities. All kinetics were performed at 35 °C and started by substrate addition in a 1 mL assay medium (acetate buffer, 0.2 M, pH = 5) containing α-mannosidase (0.025 U/mL), substrate (varying concentration from *Km*/4 to *Km* value) in presence or absence of various concentrations of inhibitor. Under these conditions, the *p*-nitrophenolate released led to optical densities linear with both reaction time and concentration of the enzyme. *K_i_* values were determined in duplicate or quadruplate, using the Dixon graphical method [72]. As the inhibitors are only partially soluble in water, they were dissolved in DMSO for concentrated mother solutions and DMSO/buffer for diluted solutions with a final DMSO concentration under 5% in all vials. Previously, the stability of the enzymes in presence of various concentrations of DMSO was controlled and the enzyme activity was unaffected.

### 3.3. Docking Studies

Docking calculations were performed with the Autodock-VINA [85], program implemented in YASARA (software version 20.7.4) [86], using a general AMBER force field [81,82,83,84]. The X-ray structure of JBα-man (Protein Data Bank, PDB entry 6B9O] [50] was used. The protein and clusters were parameterized according to AutoSMILES, see also reference [80]. For all the docked structures, some bonds of the aliphatic chains were treated as active torsional bonds. The default VINA docking parameters in YASARA macro were used. Several different starting structures for each of the multivalent clusters were optimized by molecular dynamics simulation and molecular mechanics calculations in a box of water molecules, using YASARA software, and were then used in docking studies. Regarding dendron cluster **6c**, the docking calculations were performed on a simplified cluster model bearing 4 DNJ heads in order to overcome computing power problems found during the docking studies with the complete dendron cluster **6c**.

## 4. Conclusions

A series of multivalent clusters with deoxynojirimycin inhitopes was synthesized by CuAAC. Two alkyne-armed calix[8]arene scaffolds were used, a *C_8_* symmetrical 8-valent calix[8]arene core and a *C_2v_* symmetrical 6-valent 1,5-xylylene bridged one, with the same size and a close valency but differing in their rigidity. With the shorter C6 linker, the flexibility of the scaffold plays a significant role since the *C_8_* symmetrical cluster **6a** did not show multivalent effect whereas the 1,5-xylylene bridged **7a** had an affinity improvement per inhitope of 7. Clusters with a longer C9 linker, whatever their global size, in the rigidity and valency of the central core show good affinity enhancement per inhitope in the range around a hundred (70 to 160). The best results leading to *K_i_* < 100 nM and *rp/n* > 2000 were achieved for larger clusters **6c** and **7c**, the ones with the higher valencies and larger size. These two multivalent inhibitors are the only ones in the series whose size allow for cross-linking two enzymes as shown by docking studies.

## Figures and Tables

**Table 1:** Inhibition constants, relative inhibition potencies, and relative inhibition potencies per inhibitory unit of iminosugar clusters and monovalent references against JBα-man.

Entry	Compound	Alkyl Chain Length	DNJ Unit	*K_i_*^a^ (µM)	*rp* ^b^	*rp/n* ^c^
1	**3** [35]	C6	1	322 [35]	-	-
2	**4** [65]	C9	1	188 [65]	-	-
3	**7a**	C6	6	7.7 ± 0.7	42	7
4	**7b**	C9	6	0.38 ± 0.01	495	82
5	**6a**	C6	8	80 ± 24	4	0.5
6	**6b**	C9	8	0.32 ± 0.05	588	73
7	**7c**	C9	18	0.092 ± 0.008 ^d^ 0.213 ± 0.042	2043	113
8	**6c**	C9	24	0.050±0.012 ^e^	3760	157

^a^ Means ± SD obtained from experiments performed in triplicate; ^b^ Relative inhibition potency = *K_i_* (monovalent reference **3** or **4**)/*K_i_* (cluster); ^c^ *rp*/*n* = *rp*/number of DNJ units; ^d^ mixed inhibition mode; ^e^ fast tight binding competitive inhibition mode.

## Supplementary Materials

The following are available online at https://www.mdpi.com/1424-8247/13/11/366/s1, Full structure of compounds **6a–c**, **7a–c**, **13a–c** and **14a–c**, Zoom of X-ray structure of JB -man in interaction with compound **1e** for one active site (Figure S7), ^1^H, ^13^C NMR, HSQC and HBMC spectra of new compounds and kinetic plots for *K**_i_* determination. Details on molecular docking and coordinates of multivalent clusters and their complexes with JBα-man.
