# Supplementary material for: Synthesis and Glycosidase Inhibition Properties of Calix[8]arene-Based Iminosugar Click Clusters
Source: Pharmaceuticals (Basel). 2020 Nov 5;13(11):366. doi: 10.3390/ph13110366 (PMC7694328; doi:10.3390/ph13110366)
Supplement: Supplementary file 1 [file pharmaceuticals-13-00366-s001.zip › pharmaceuticals-982572 -suppl/pharmaceuticals-982572 -suppl.pdf]

Electronic Supplementary information

Synthesis and Glycosidase Inhibition Properties of Calix[8]arene-based Iminosugar Click Clusters

Jérémy P. Schneider, Stefano Tommasone, Paolo Della Sala, Carmine Gaeta, * Carmen Talotta, Celine Tarnus, Placido Neri,*Anne Bodlenner* and Philippe Compain*

Table of contents

Structure of compounds **13a** and **6a** S2
Structure of compounds **13b** and **6b** S3
Structure of compounds **14a** and **7a** S4
Structure of compounds **14b** and **7b** S5
Structure of compounds **13c** and **6c** S6
Structure of compounds **14c** and **7c** S6
Zoom of X-Ray structure of JB α-man in interaction with compound **1e** for one active site S7
^{1}H, ^{13}C NMR, HSQC and HBMC spectra of compounds **10**, **11**, **13 a-c**, **14 a-c**, **6 a-c**, **7 a-c** S8-S38
Kinetic plots for K_i determination S39-S42
Docking results for compounds **6a-b** S43-S44

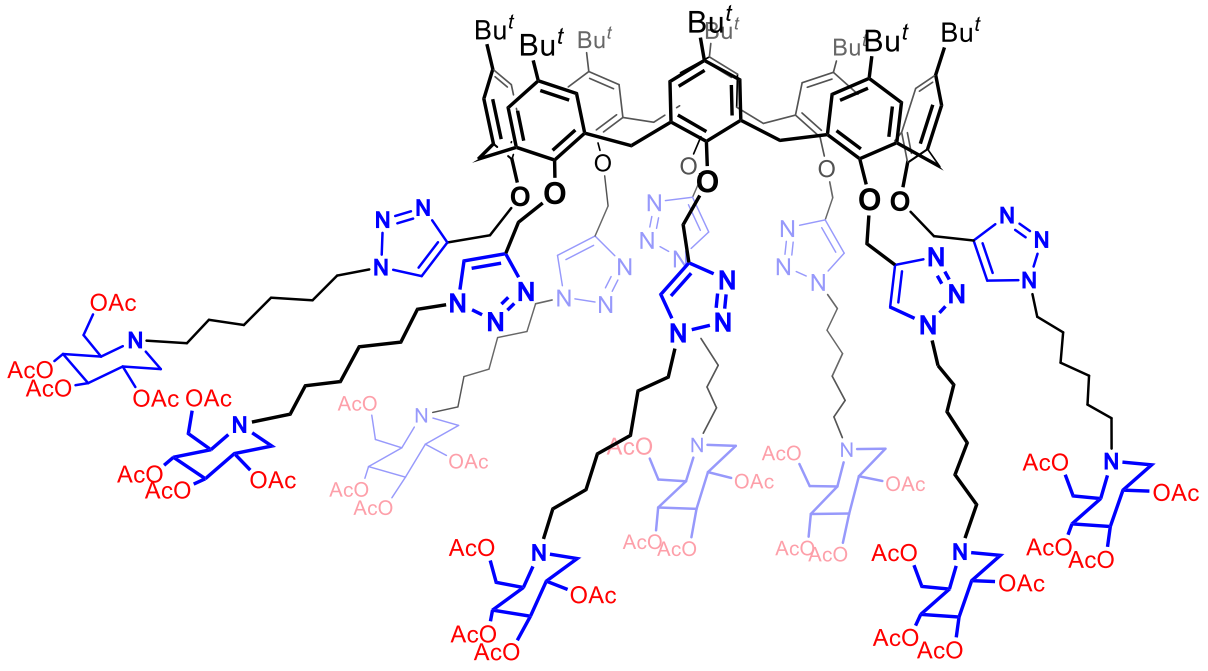

13a

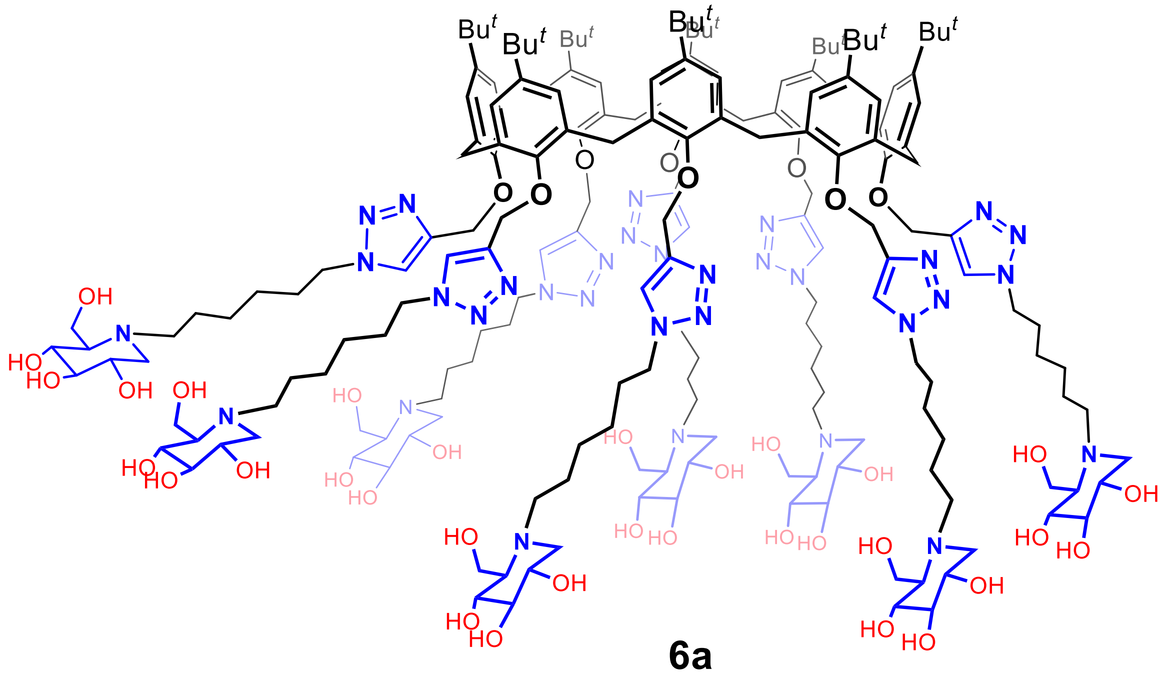

6a

Figure S1. Structure of compounds **13a** and **6a**.

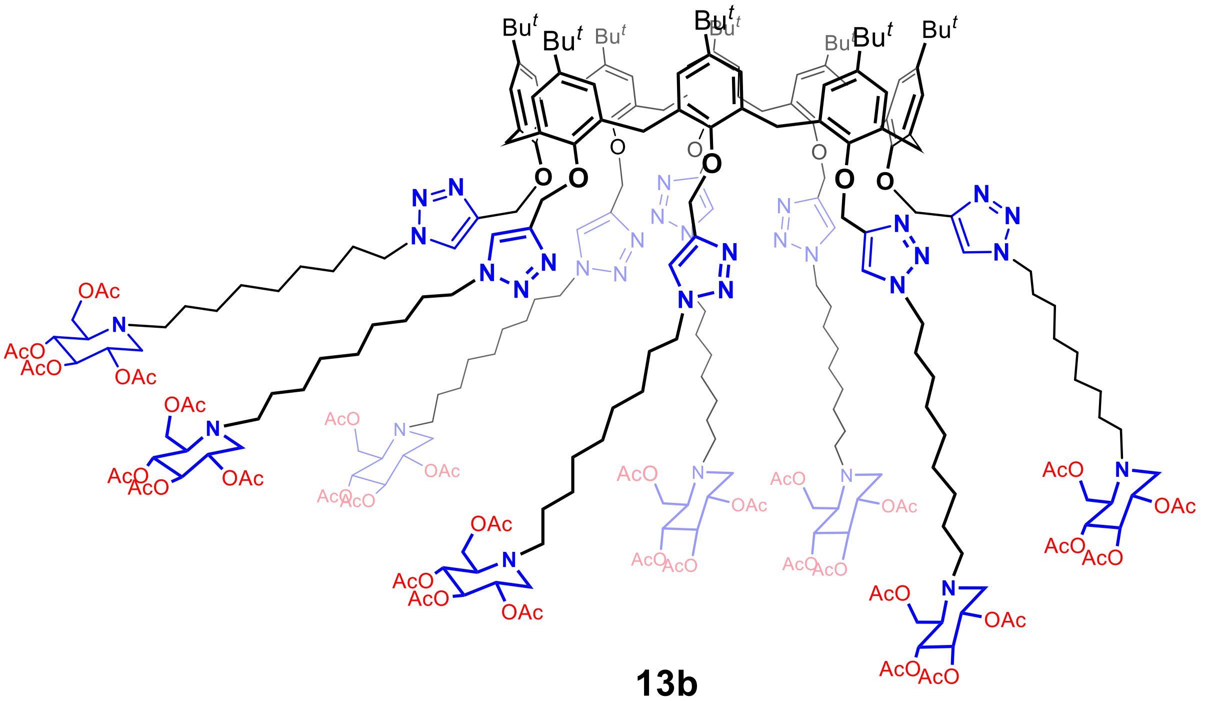

13b

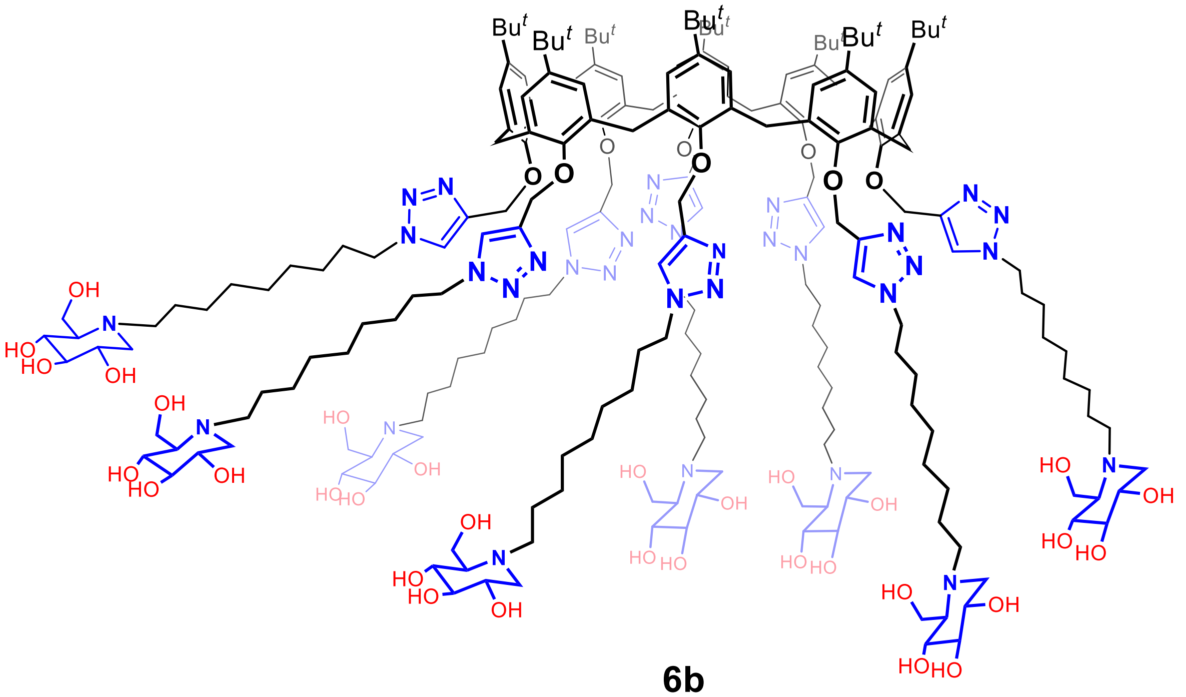

6b

Figure S2. Structure of compounds **13b** and **6b.**

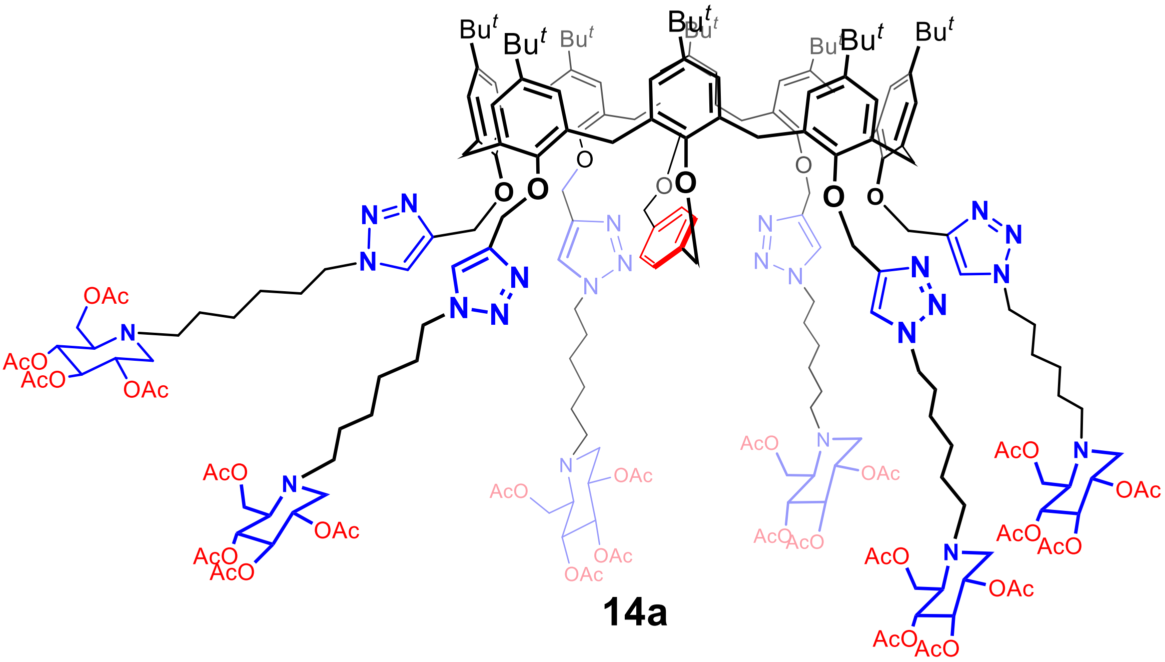

14a

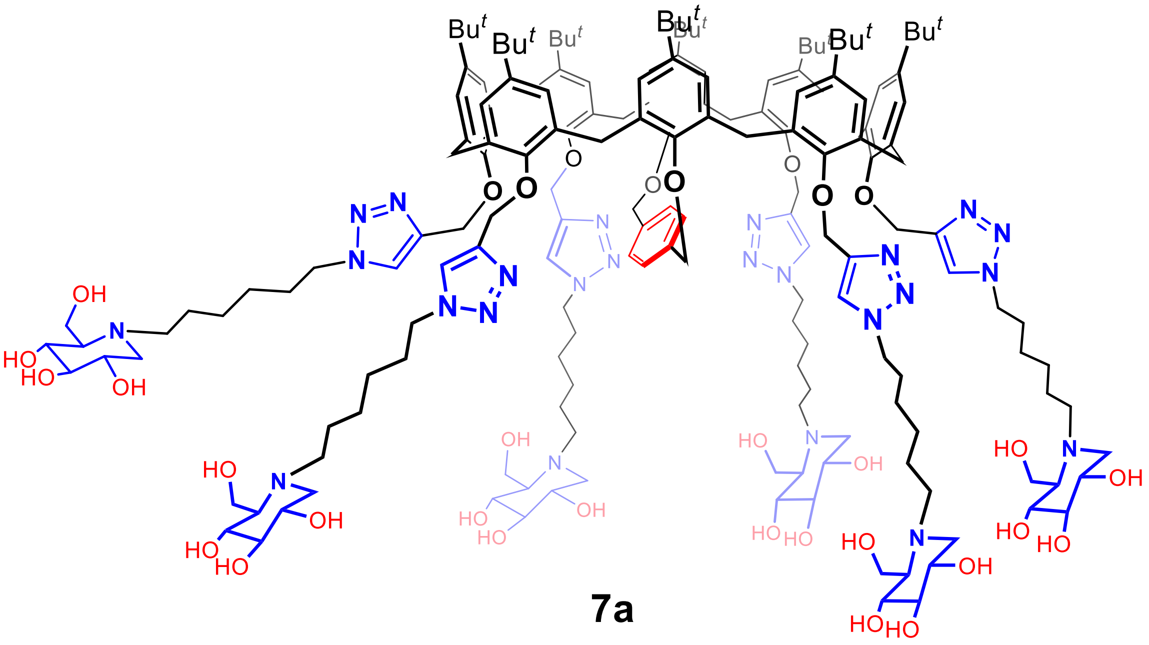

7a

Figure S3. Structure of compounds **14a** and **7a**.

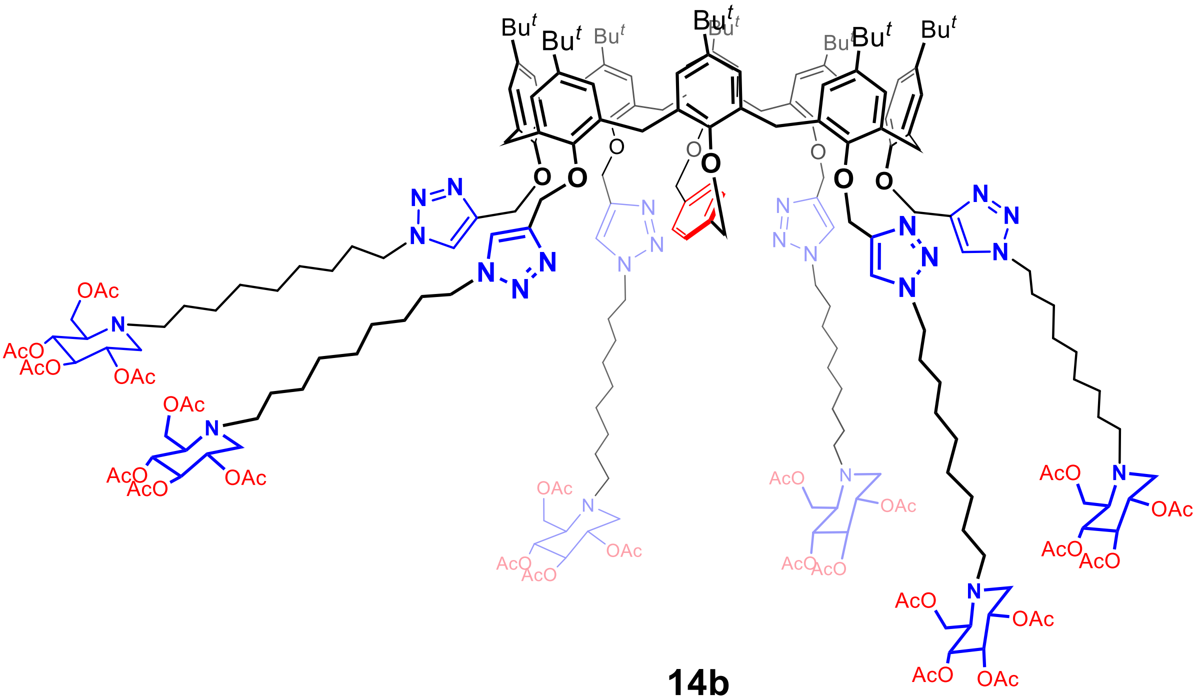

14b

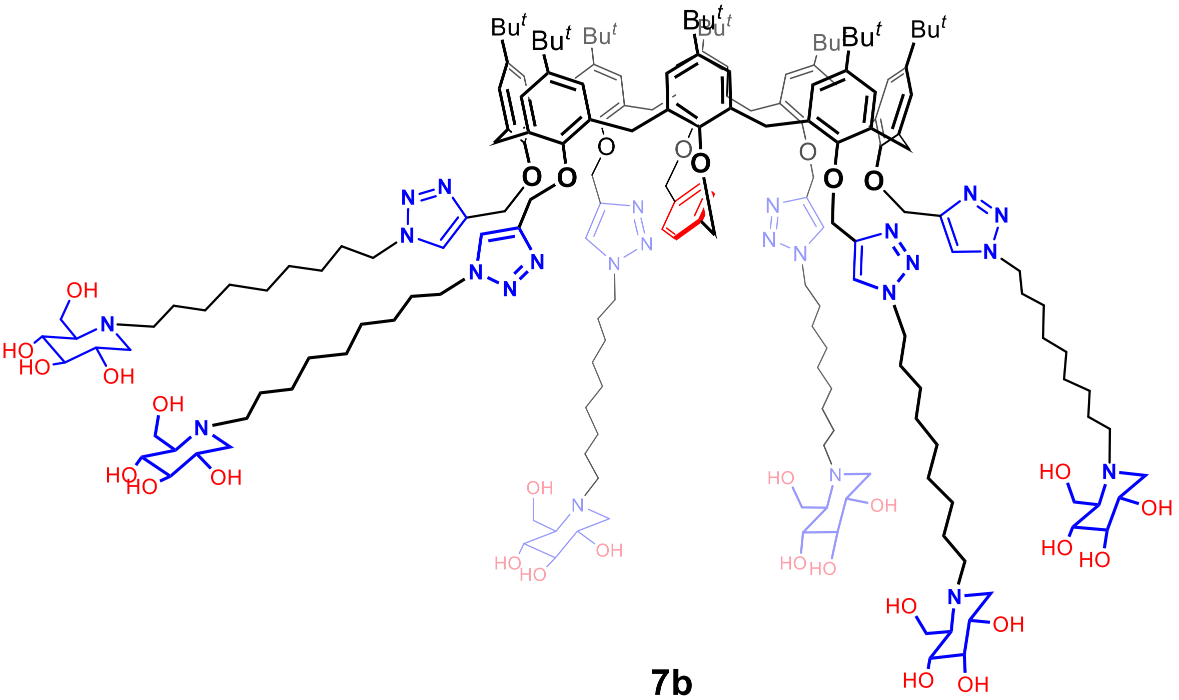

7b

Figure S4. Structure of compounds **14b** and **7b**.

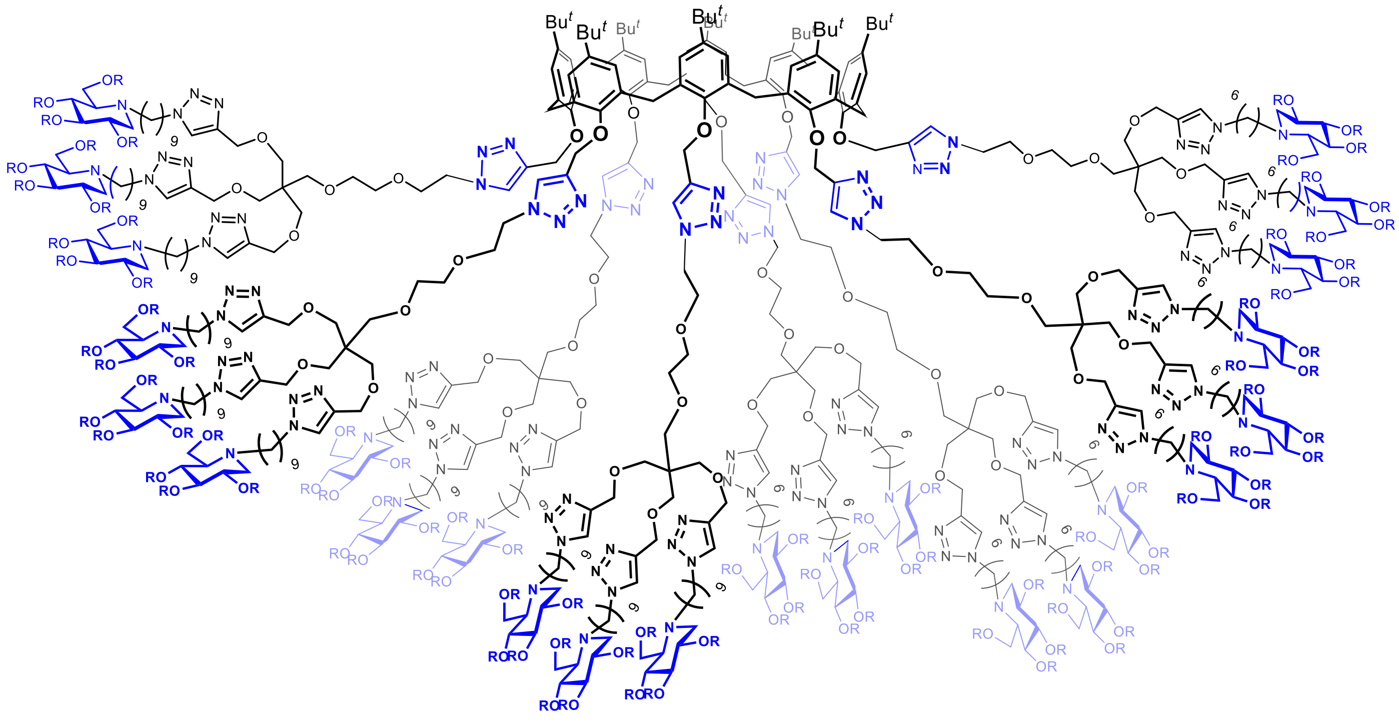

13c R = Ac

6c R = H

Figure S5. Structure of compounds **13c** and **6c**

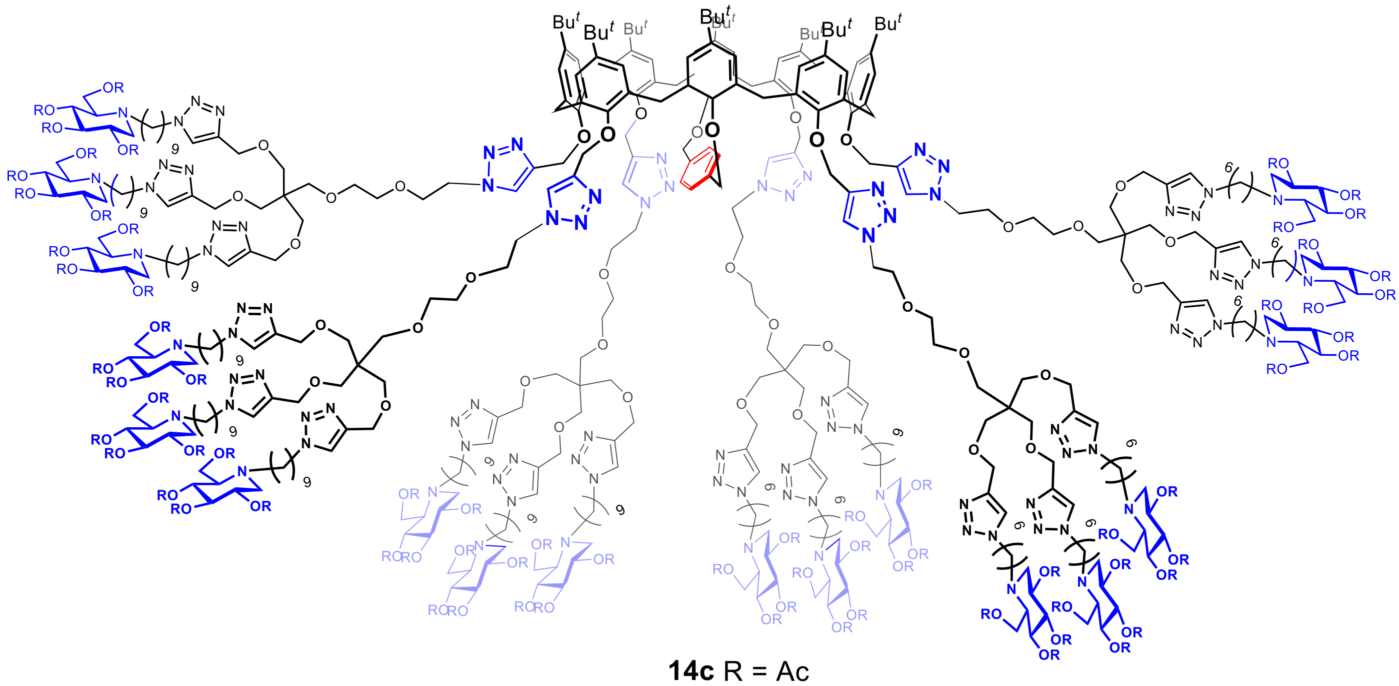

14c R = Ac

7c R = H

Figure S6. Structure of compounds **14c** and **7c**

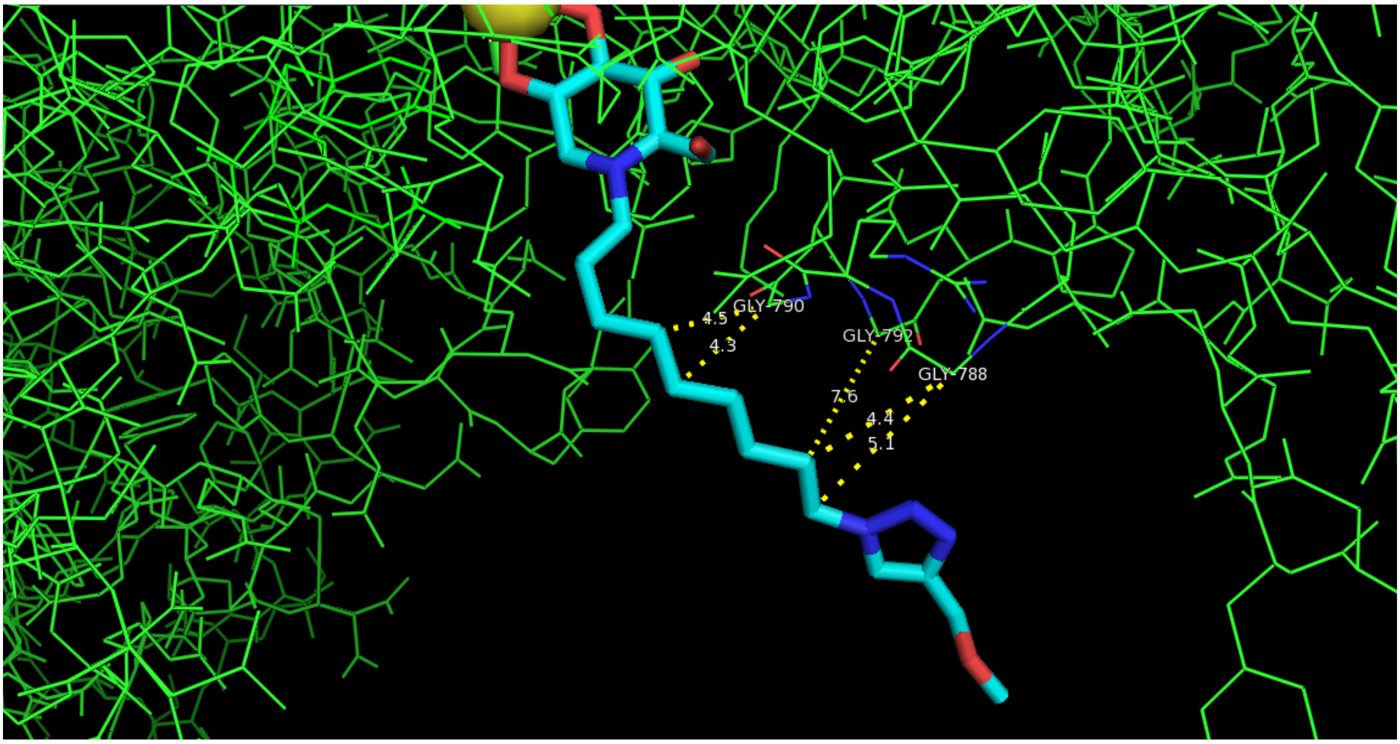

Figure S7. Glycin-rich lipophilic entry of the active site of JB α-man in interaction with the C9 alkyl chain of compound **1e** (PDB entry 6B9P).

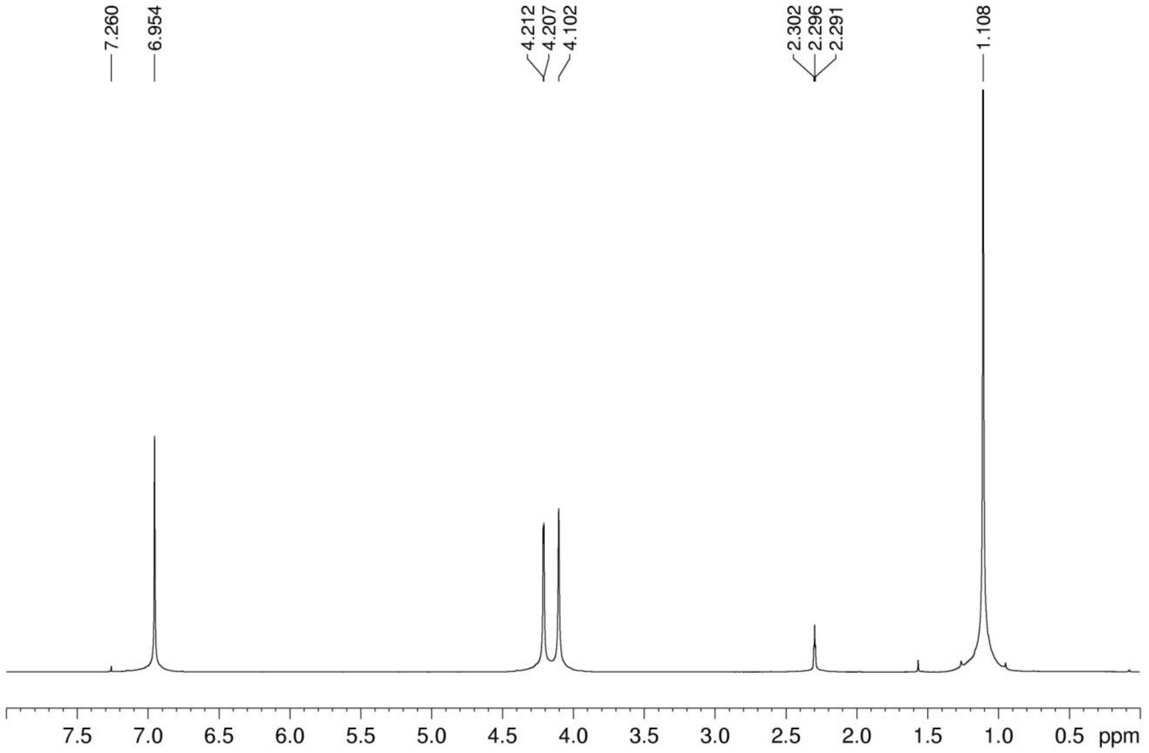

Figure S8. ^{1}H NMR spectrum of compound **10** ($CDCl_3$, 400 MHz).

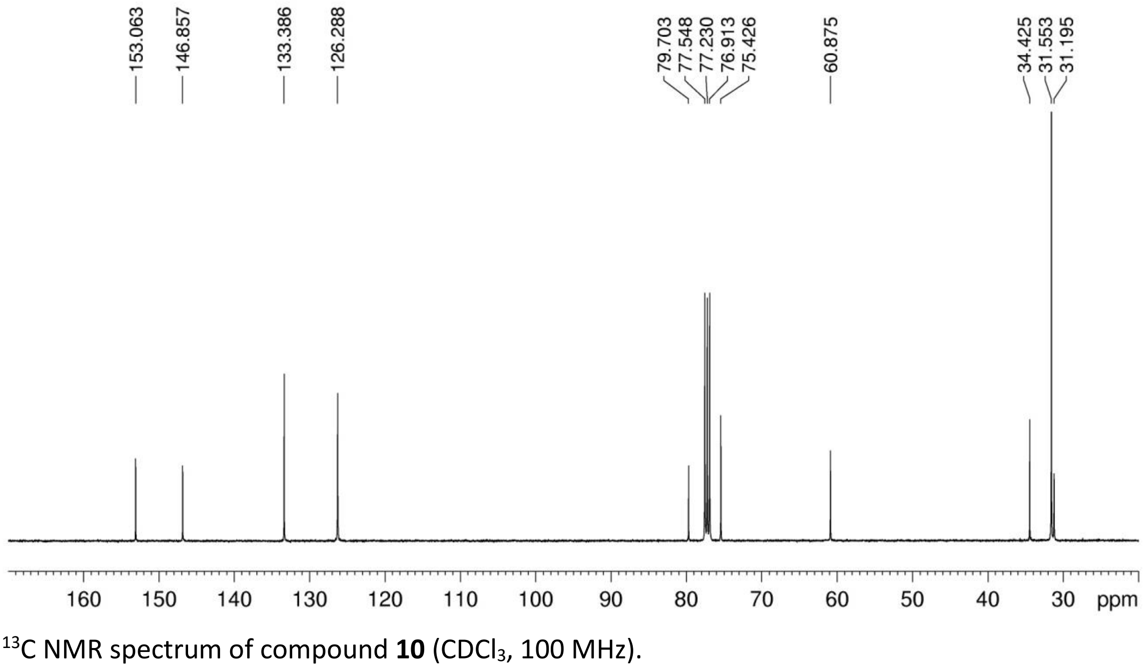

Figure S9. ^{13}C NMR spectrum of compound **10** ($CDCl_3$, 100 MHz).

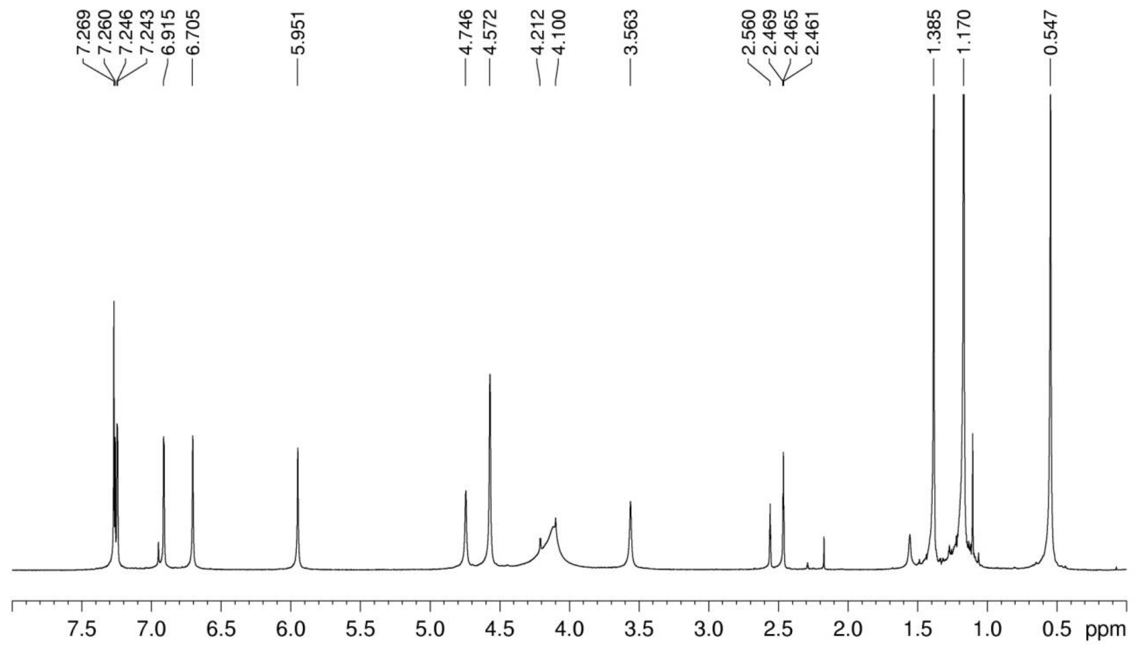

Figure S10. ^{1}H NMR spectrum of compound **11** ($CDCl_3$, 600 MHz).

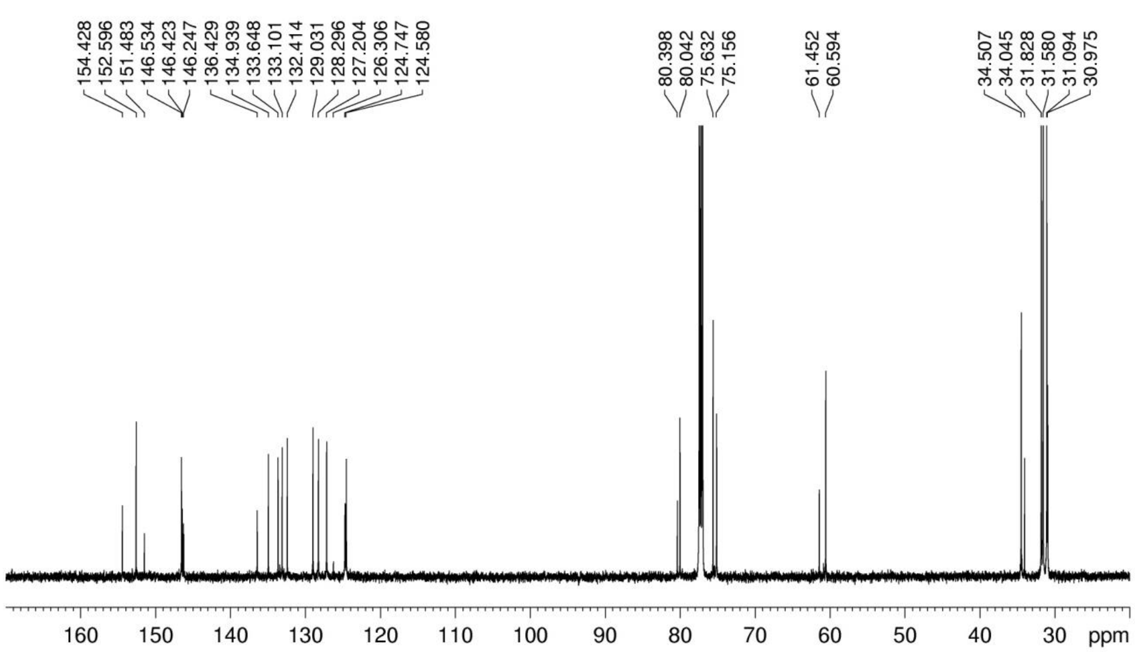

Figure S11. ^{13}C NMR spectrum of compound **11** ($CDCl_3$, 150 MHz).

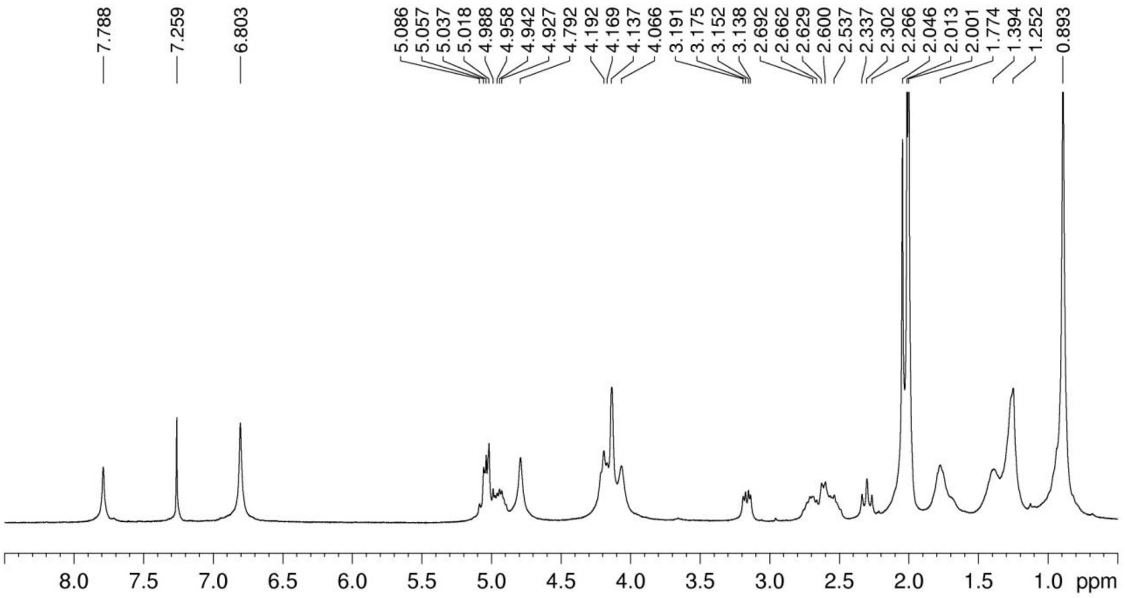

Figure S12. ^{1}H NMR spectrum of compound **13a** ($CDCl_3$, 300 MHz).

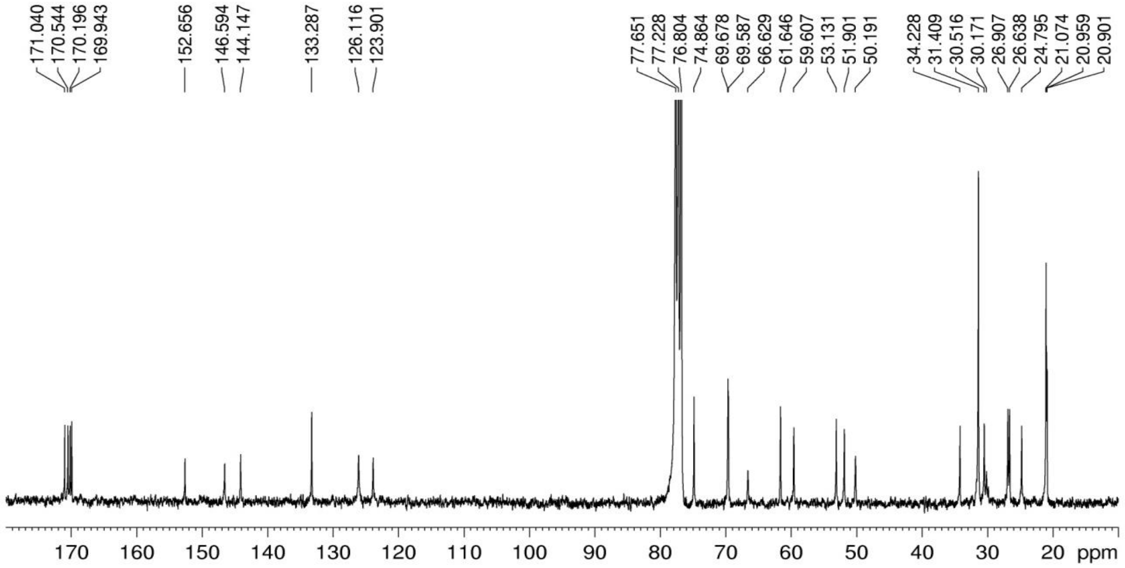

Figure S13. ^{13}C NMR spectrum of compound **13a** ($CDCl_3$, 75 MHz).

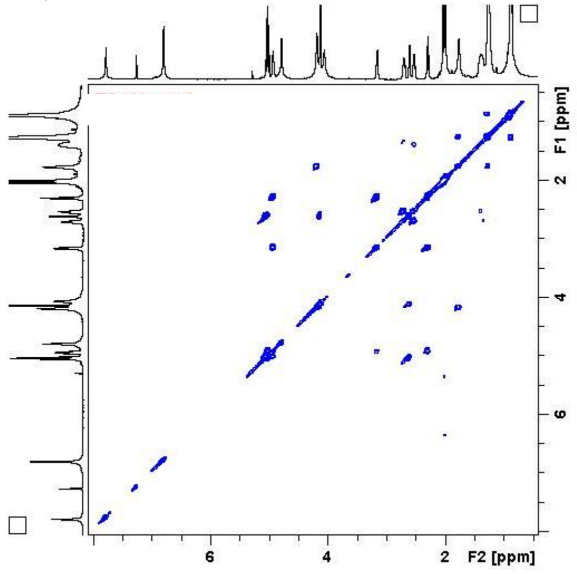

Figure S14. 2D-COSY spectrum of compound **13a** ($CDCl_3$, 600 MHz).

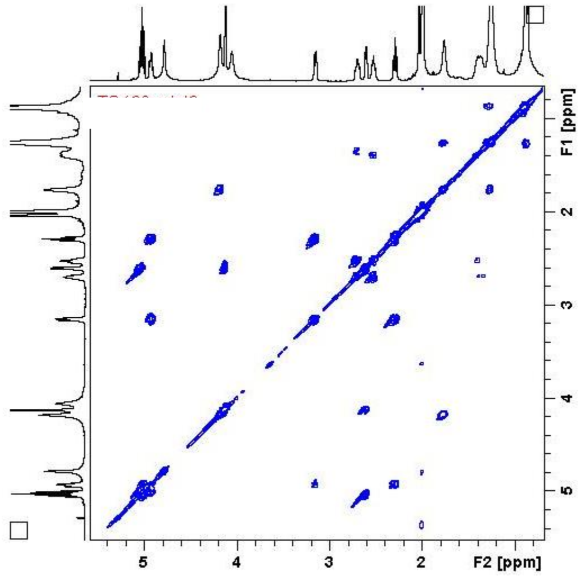

Figure S15. 2D-COSY NMR spectrum of compound **13a** -expansion ($CDCl_3$, 600 MHz).

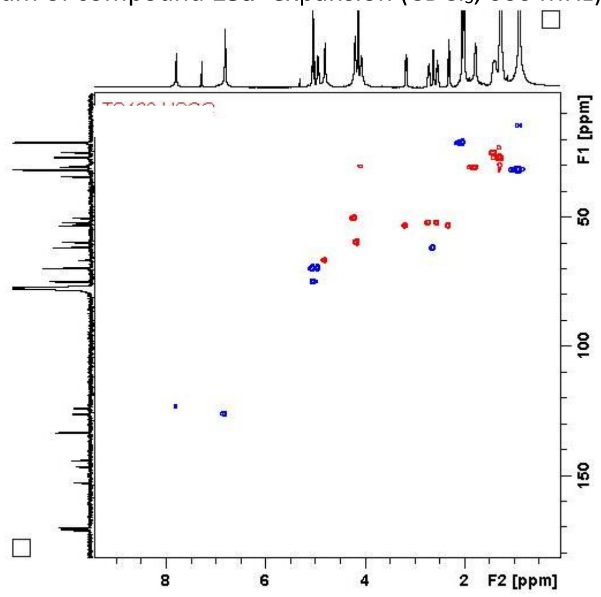

Figure S16. 2D-HSQC NMR spectrum of compound **13a** ($CDCl_3$, 600 MHz).

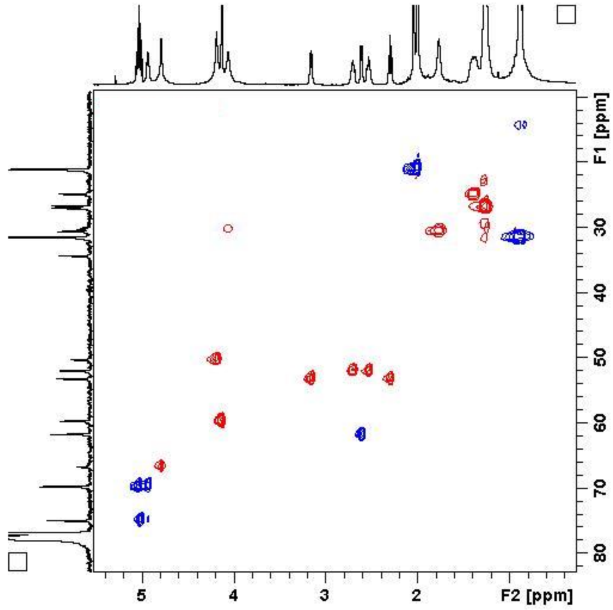

Figure S17. 2D-HSQC NMR spectrum of compound **13a** -expansion ($CDCl_3$, 600 MHz).

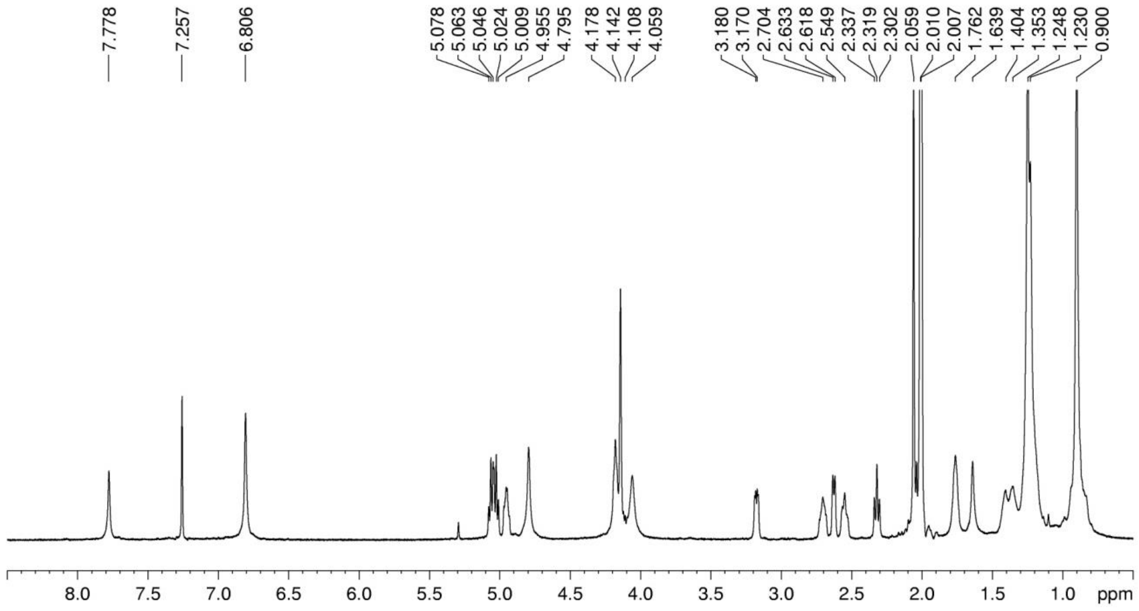

Figure S18. ^{1}H NMR spectrum of compound **13b** ($CDCl_3$, 600 MHz).

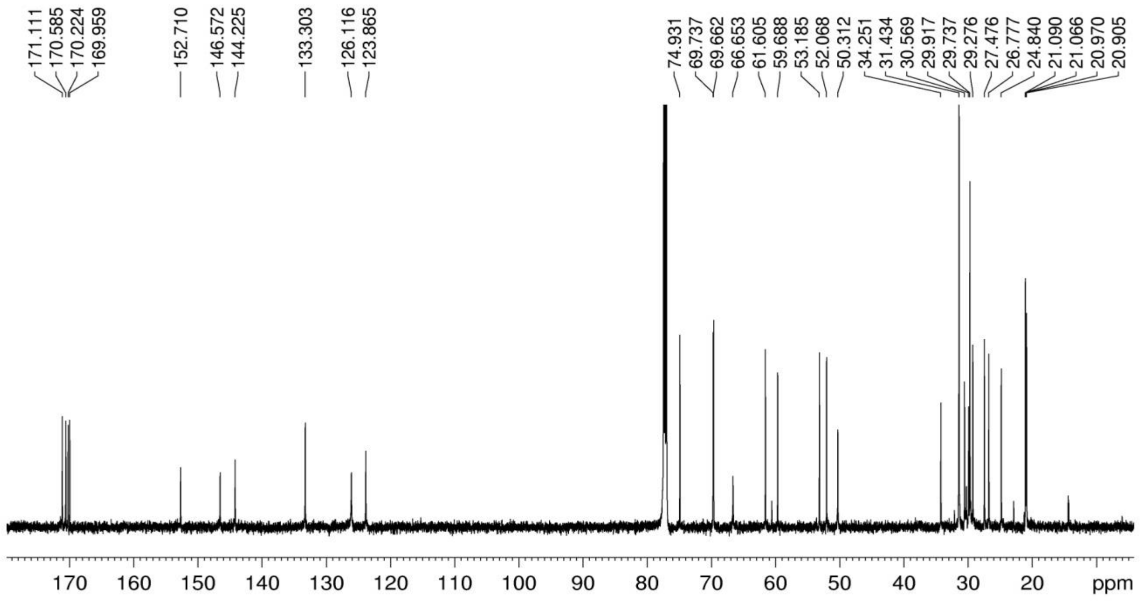

Figure S19. ^{13}C NMR spectrum of compound **13b** ($CDCl_3$, 150 MHz).

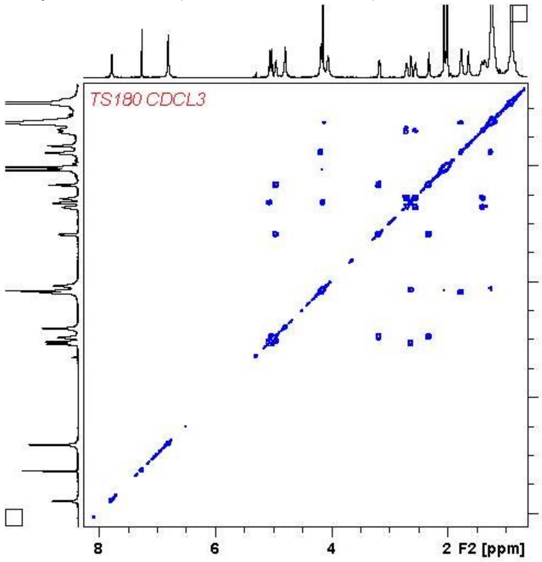

Figure S20. 2D-COSY spectrum of compound **13b** ($CDCl_3$, 600 MHz).

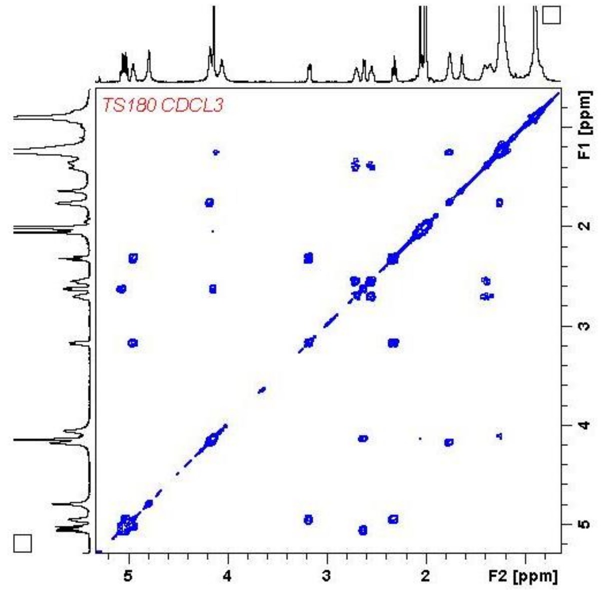

Figure S21. 2D-COSY NMR spectrum of compound **13b** -expansion ($CDCl_3$, 600 MHz).*

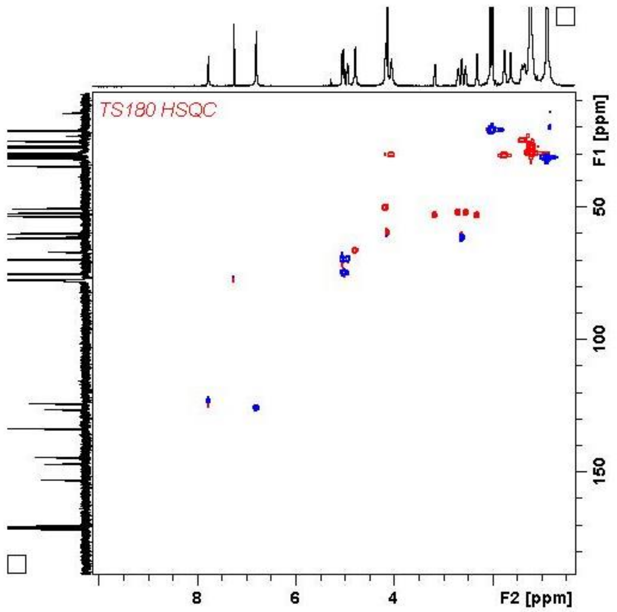

Figure S22. 2D-HSQC NMR spectrum of compound **13b** ($CDCl_3$, 600 MHz).

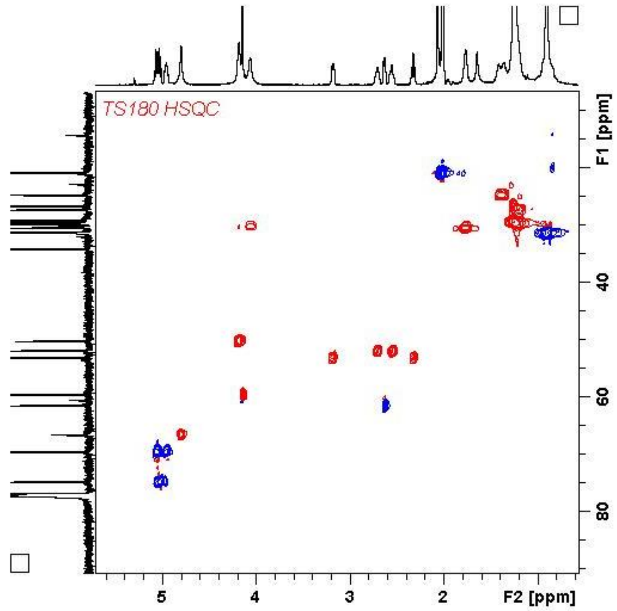

Figure S23. 2D-HSQC NMR spectrum of compound **13b** -expansion ($CDCl_3$, 600 MHz).

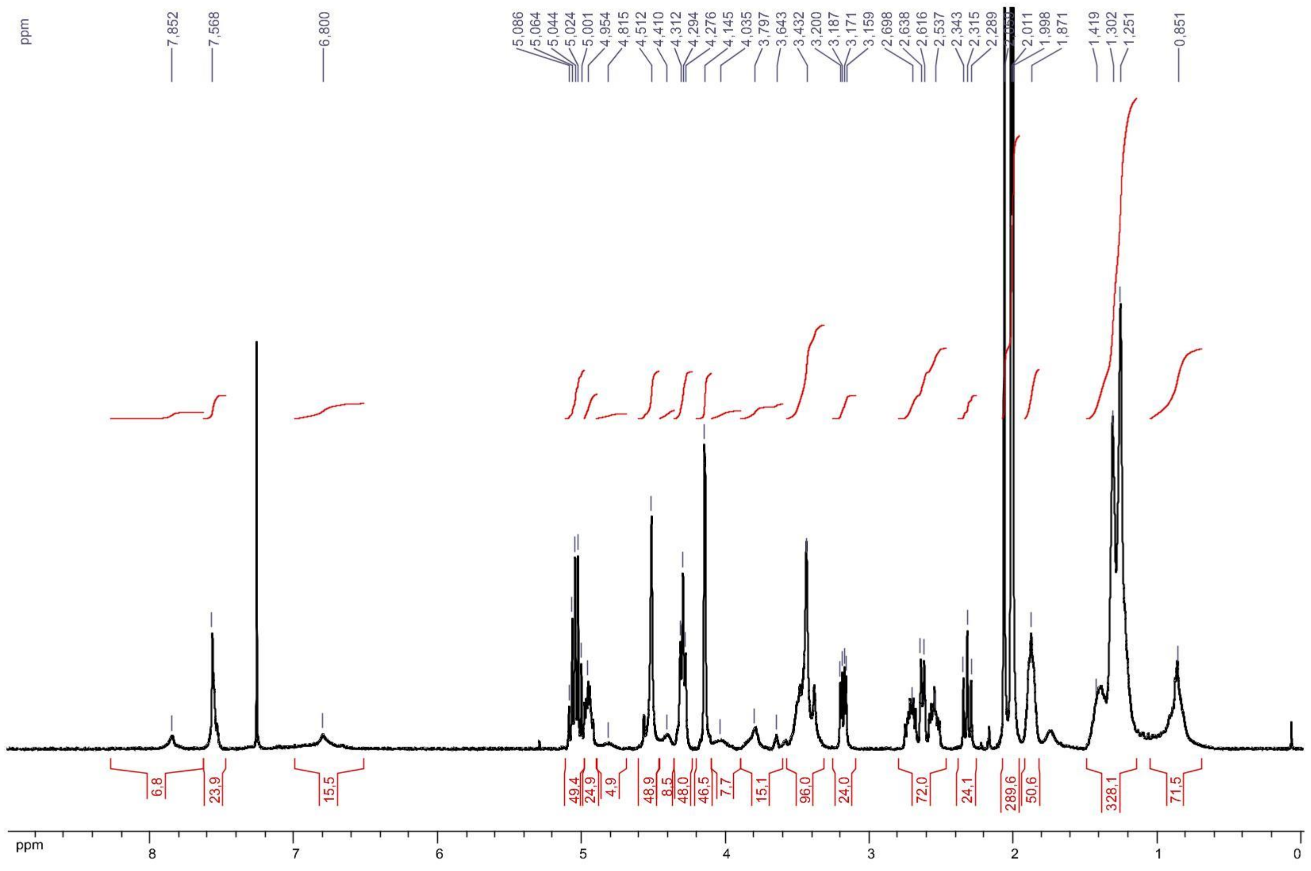

Figure S24. ^{1}H NMR spectrum of compound **13c** ($CDCl_3$, 400 MHz).

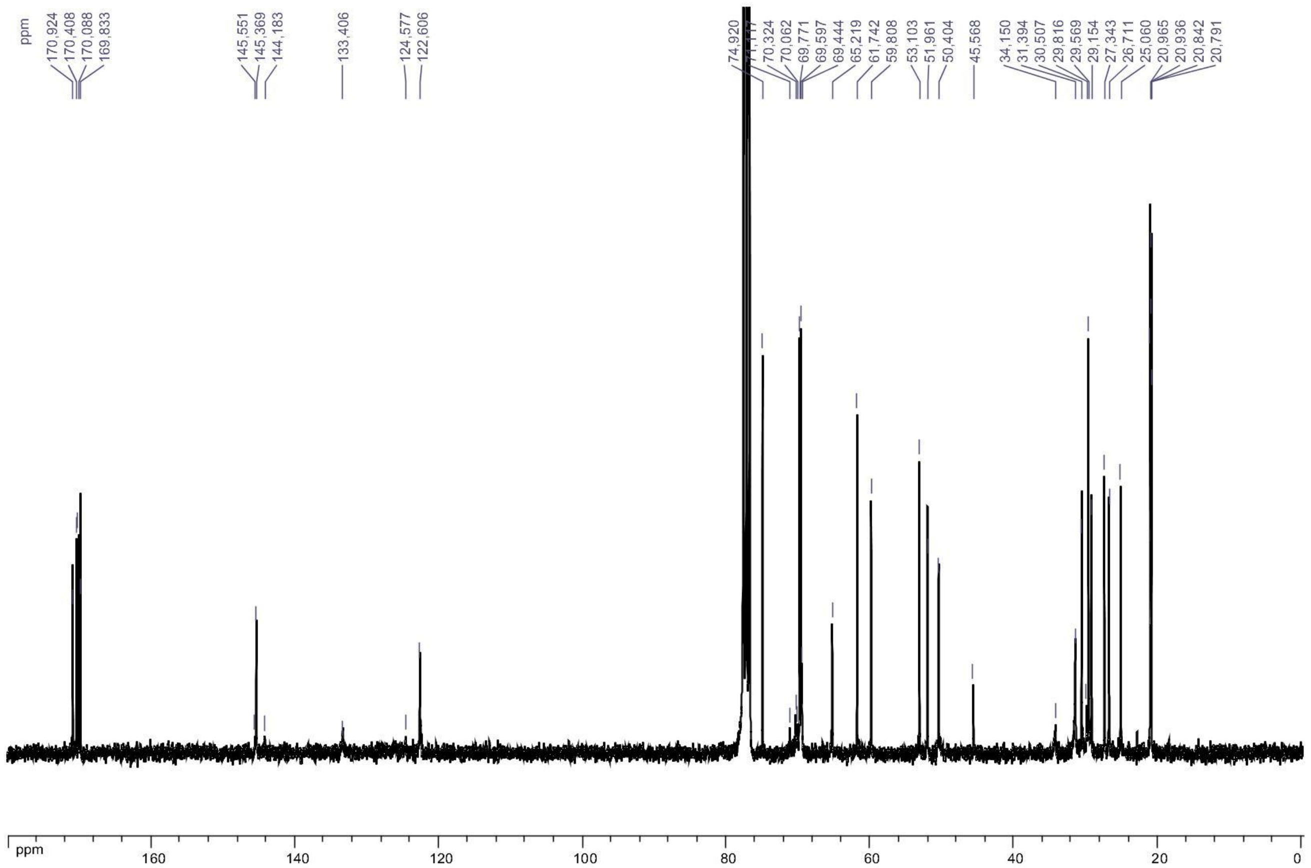

Figure S25. ^{13}C NMR spectrum of compound **13c** ($CDCl_3$, 75 MHz, 10000 scans).

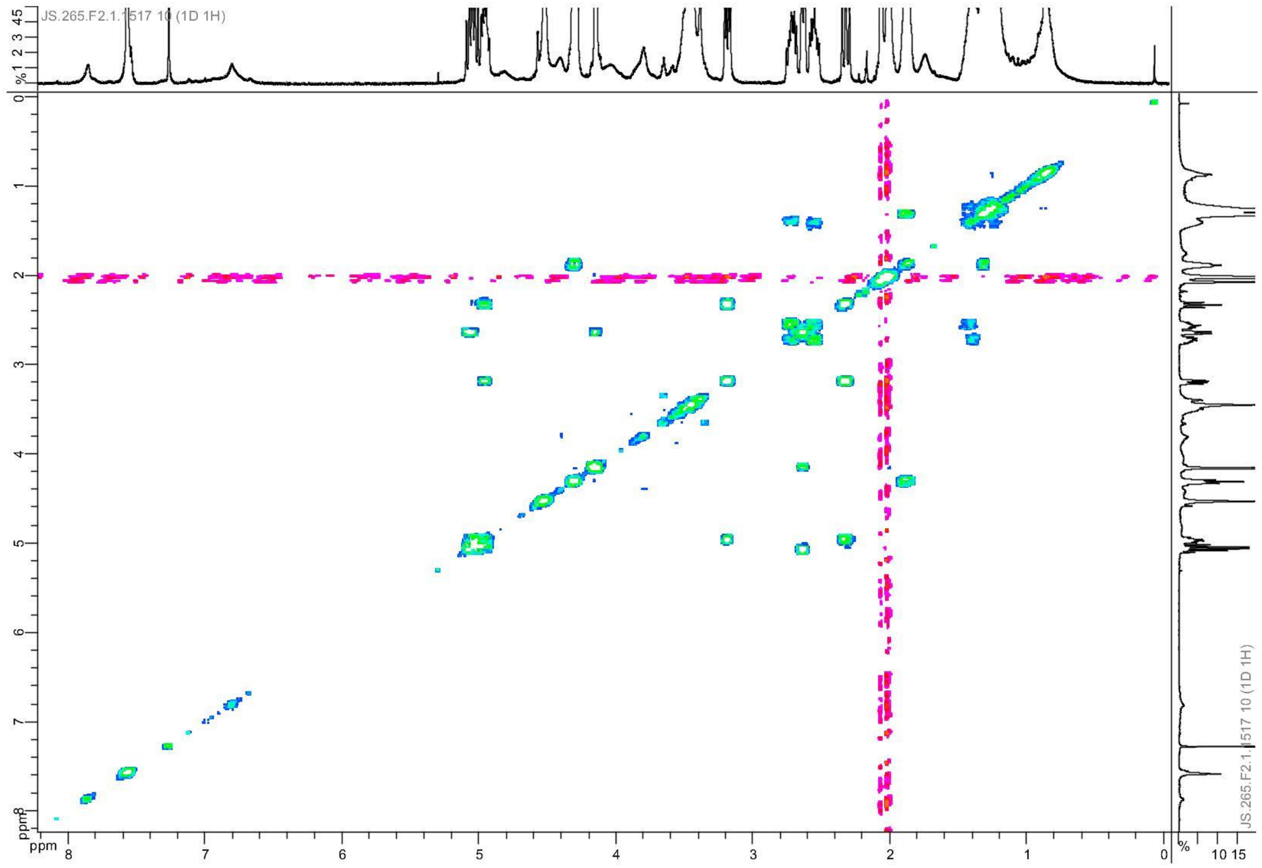

Figure S26. 2D-COSY spectrum of compound **13c** ($CDCl_3$, 400 MHz).

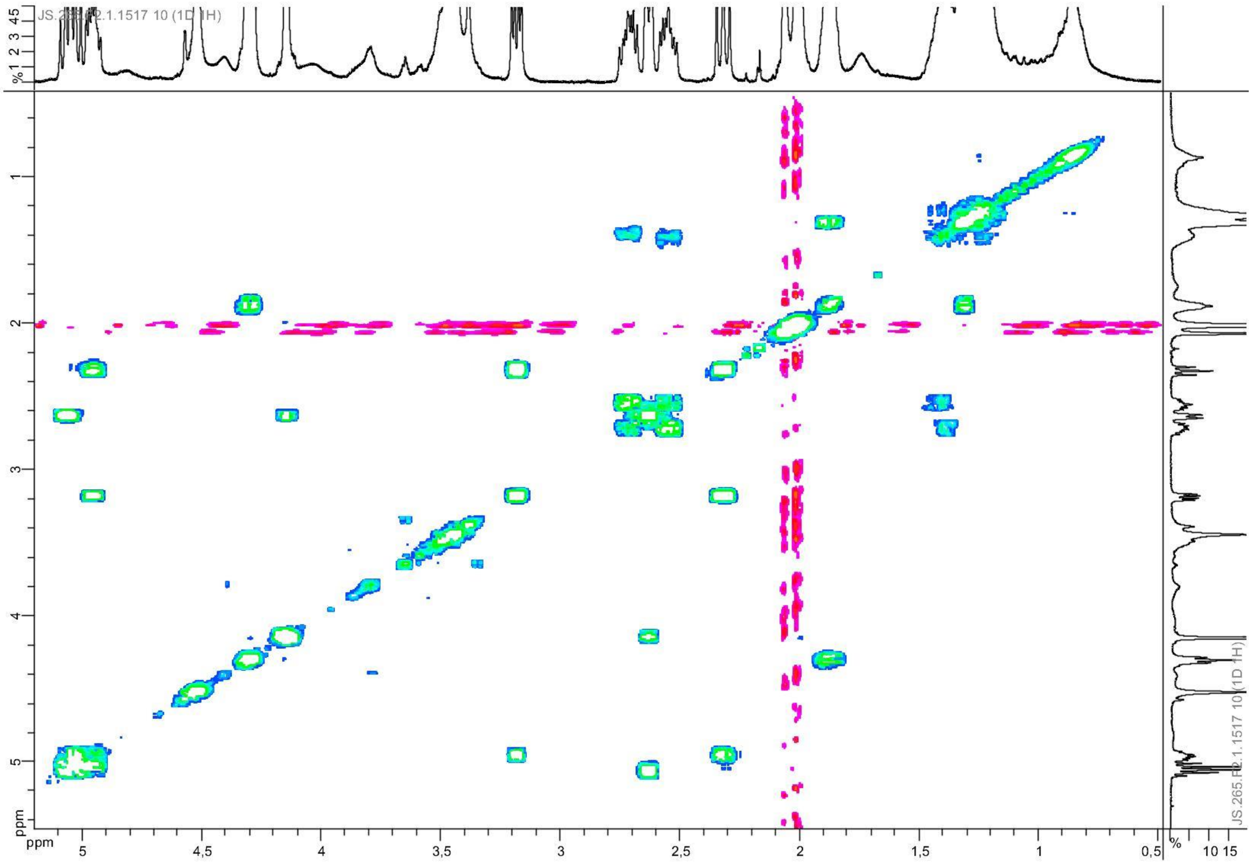

Figure S27. 2D-COSY NMR spectrum of compound **13c** -expansion ($CDCl_3$, 400 MHz).

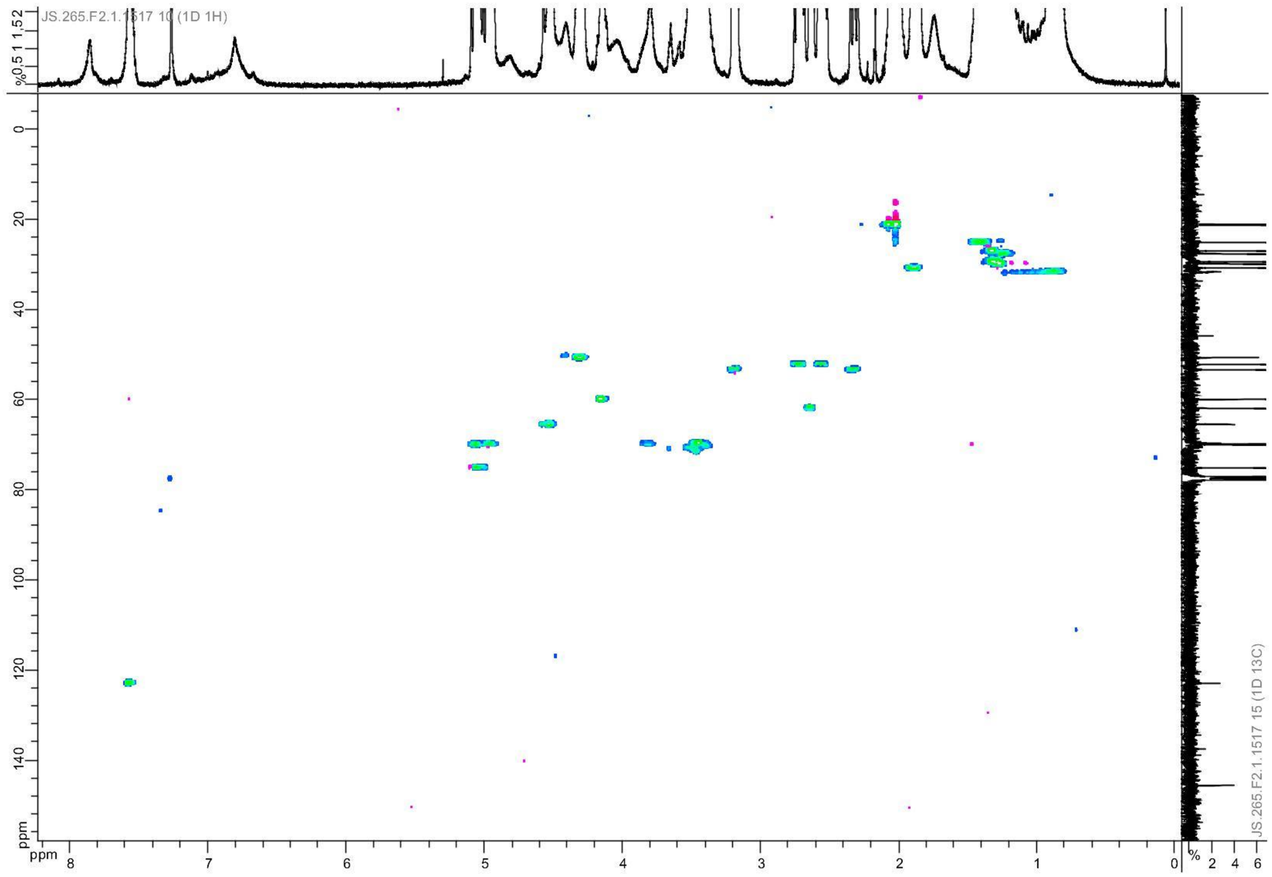

Figure S28. 2D-HSQC NMR spectrum of compound **13c** ($CDCl_3$, 400 MHz).

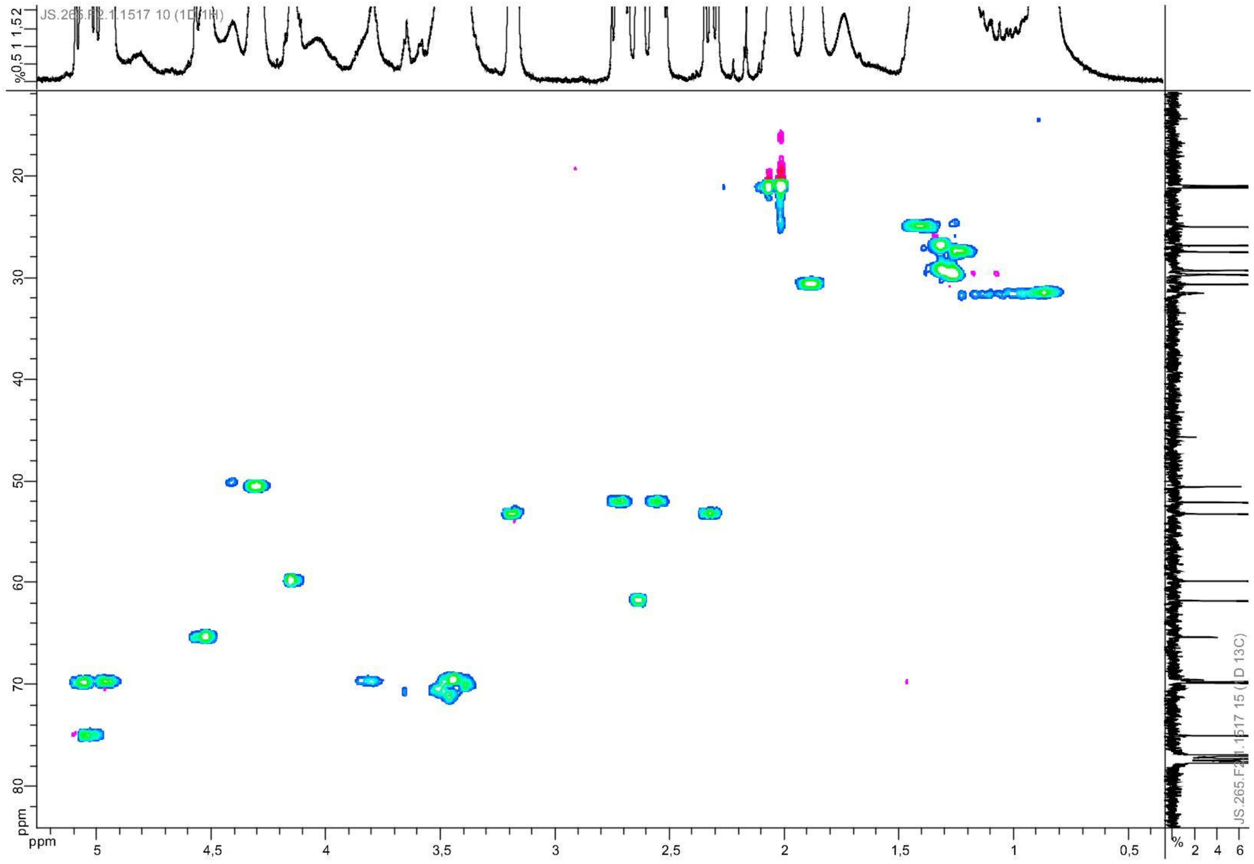

Figure S29. 2D-HSQC NMR spectrum of compound **13c** -expansion ($CDCl_3$, 400 MHz).

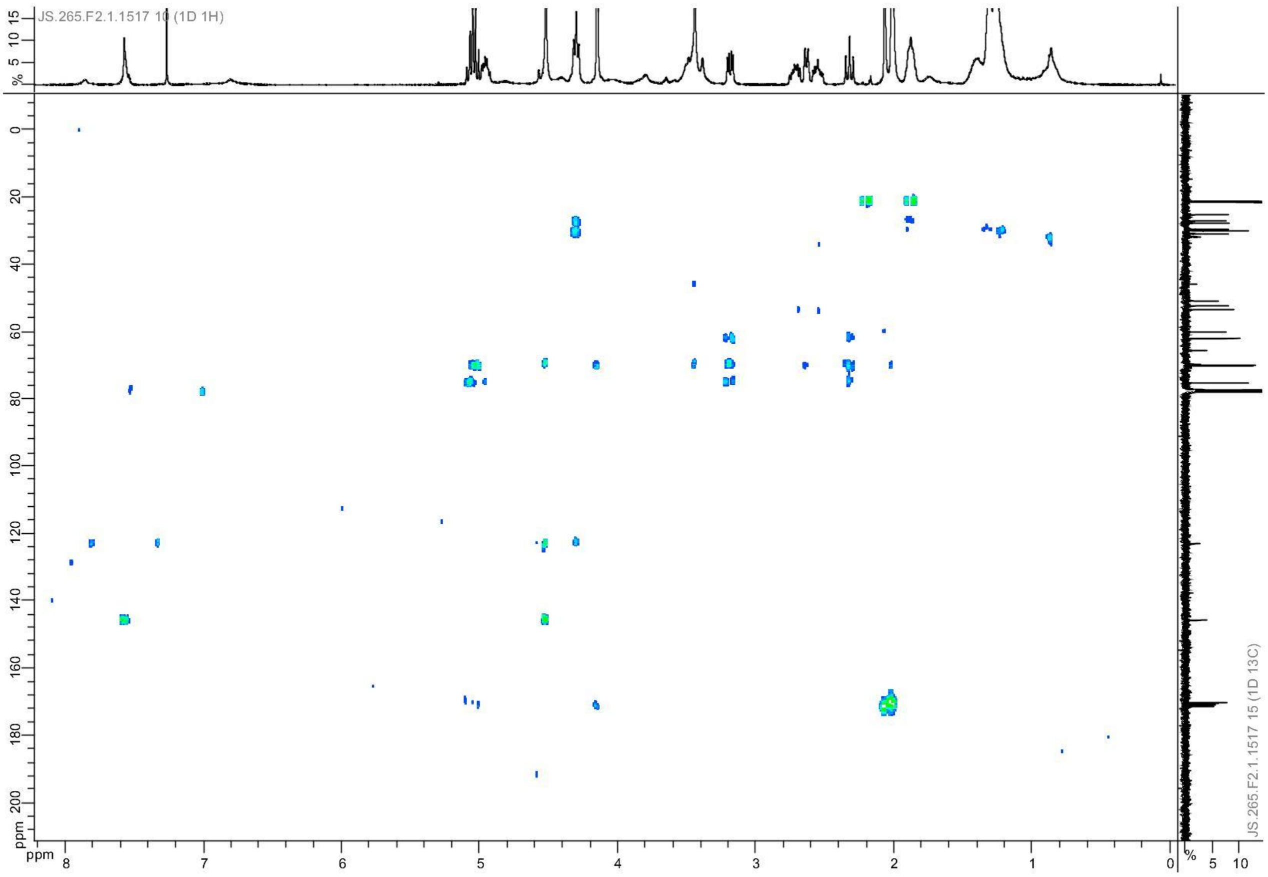

Figure S30. 2D-HMBC NMR spectrum of compound **13c** ($CDCl_3$, 400 MHz).

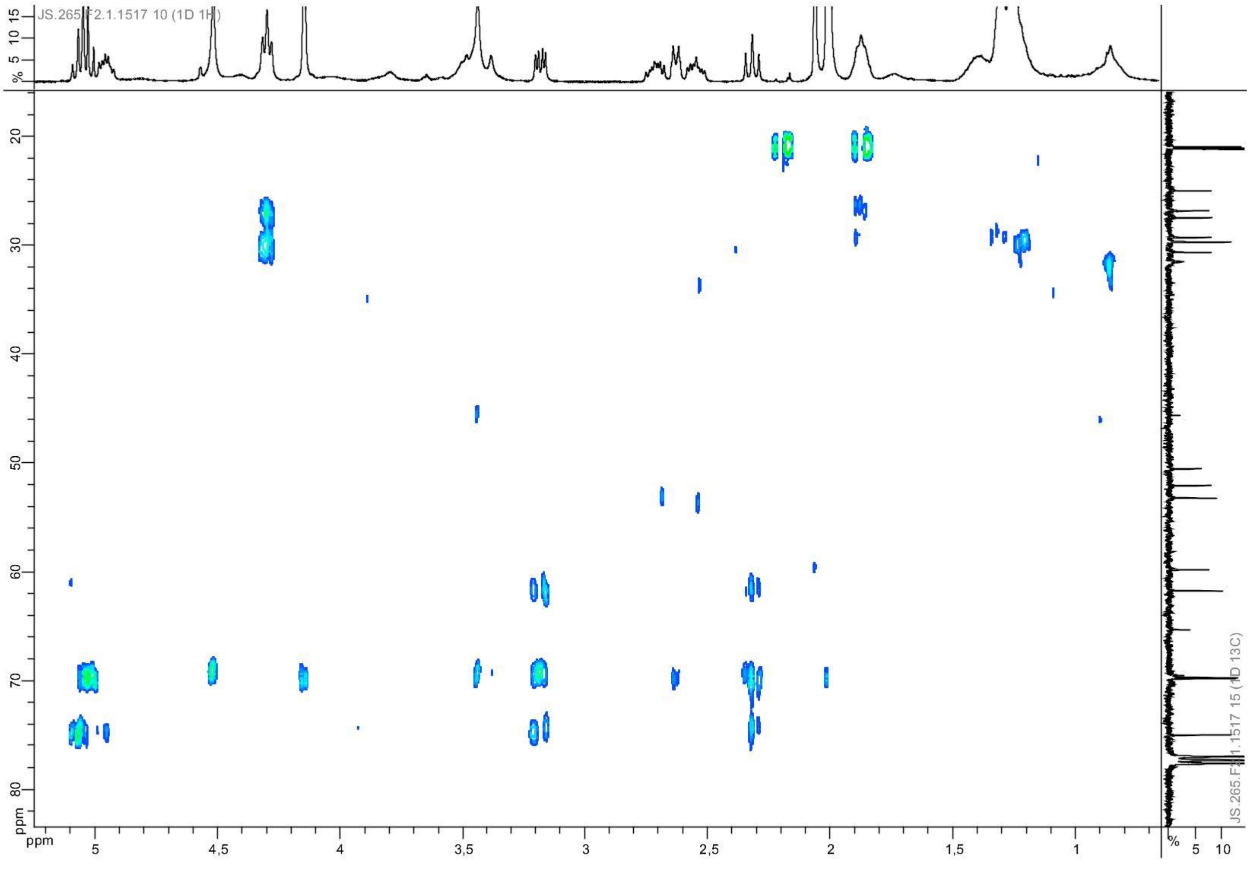

Figure S31. 2D-HMBC NMR spectrum of compound **13c** -expansion ($CDCl_3$, 400 MHz).

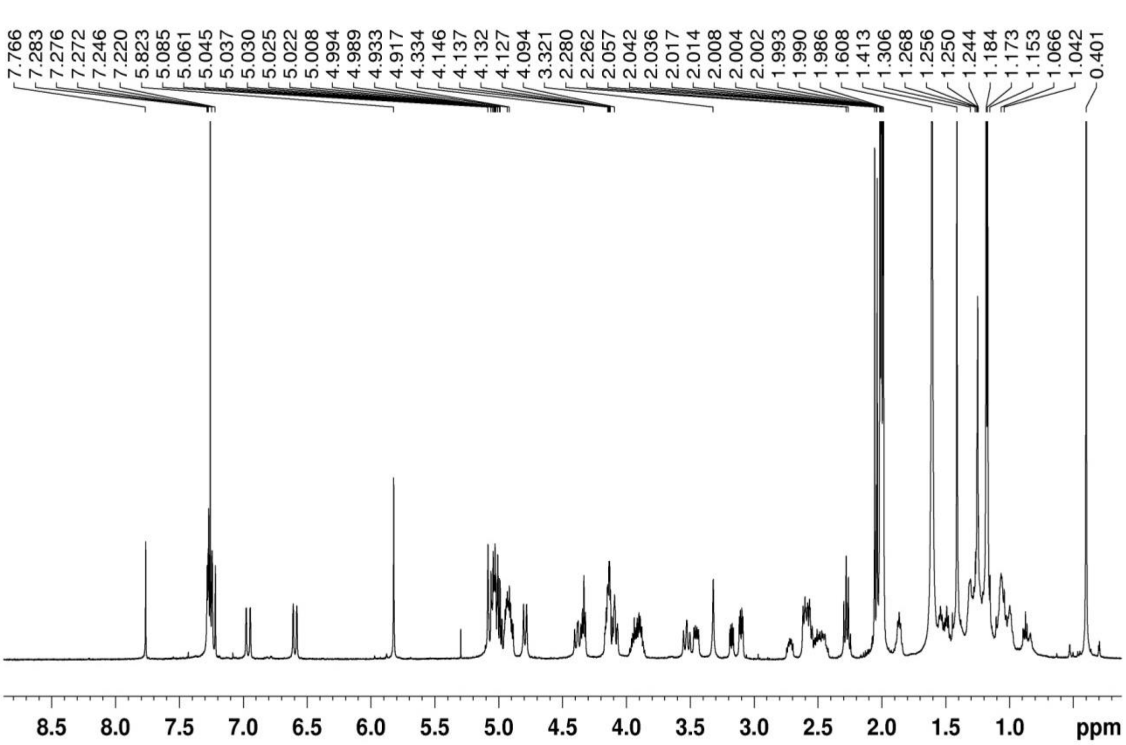

Figure S32. ^{1}H NMR spectrum of compound **14a** ($CDCl_3$, 600 MHz).

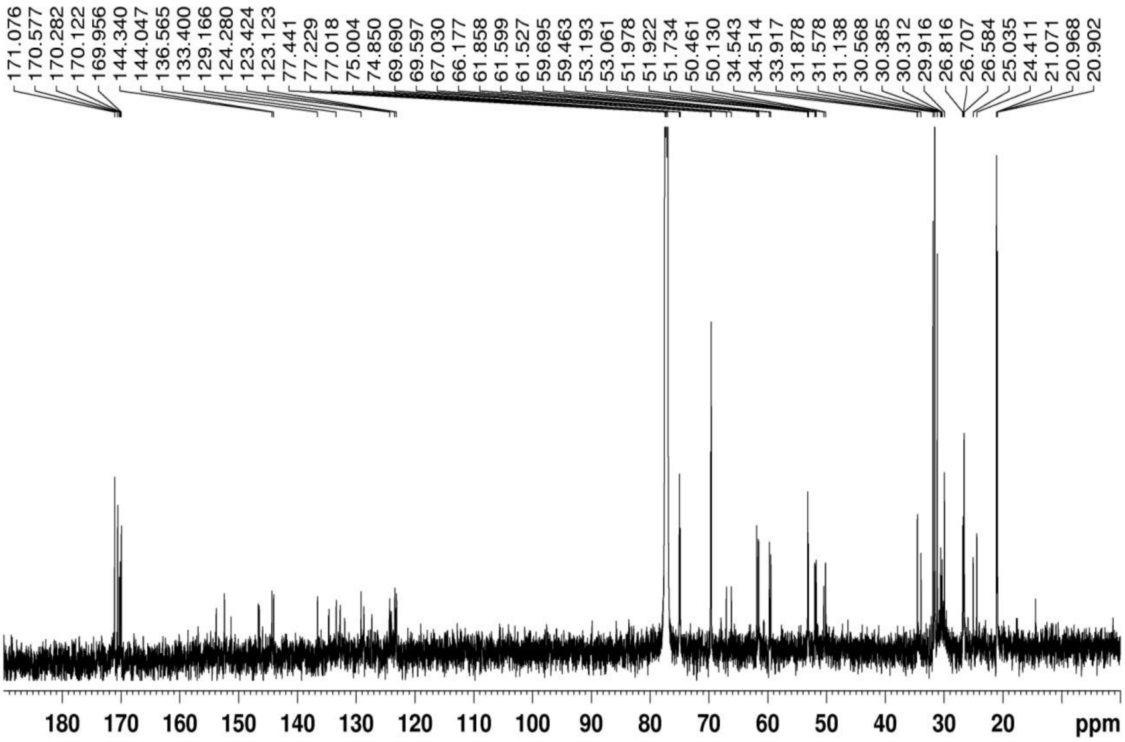

Figure S33. ^{13}C NMR spectrum of compound **14a** ($CDCl_3$, 150 MHz).

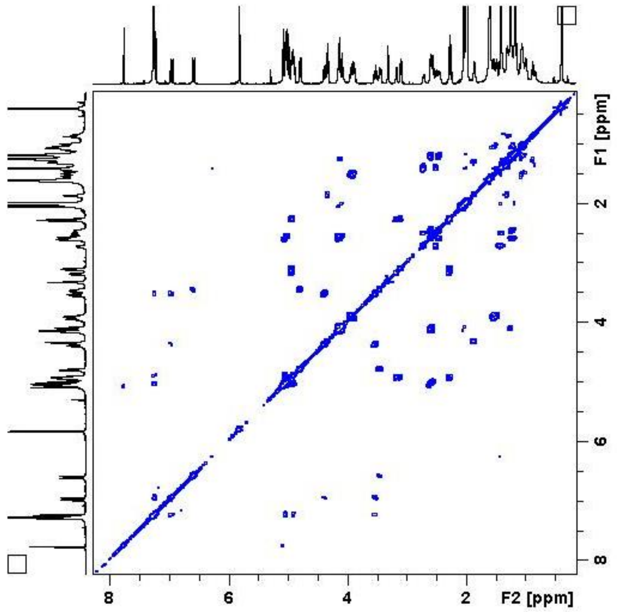

Figure S34. 2D-COSY spectrum of compound **14a** ($CDCl_3$, 600 MHz).

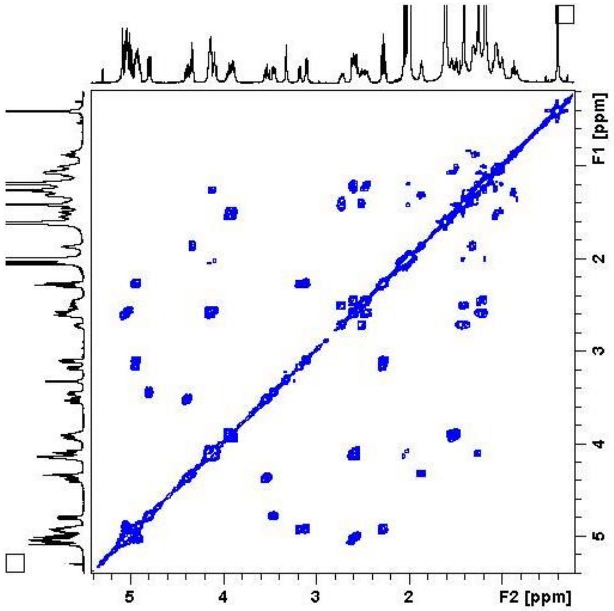

Figure S35. 2D-COSY NMR spectrum of compound **14a** -expansion ($CDCl_3$, 600 MHz).

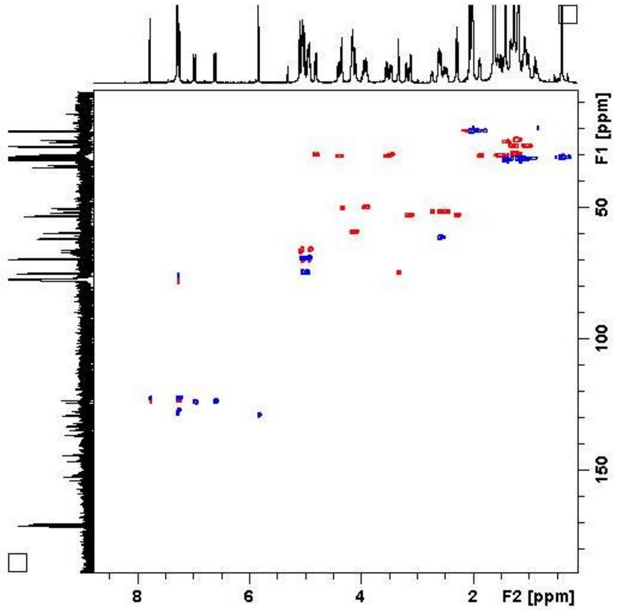

Figure S36. 2D-HSQC NMR spectrum of compound **14a** ($CDCl_3$, 600 MHz).

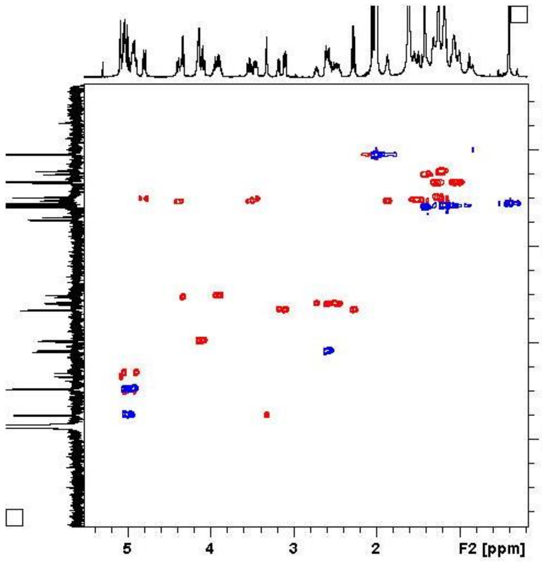

Figure S37. 2D-HSQC NMR spectrum of compound **14a** -expansion ($CDCl_3$, 600 MHz).

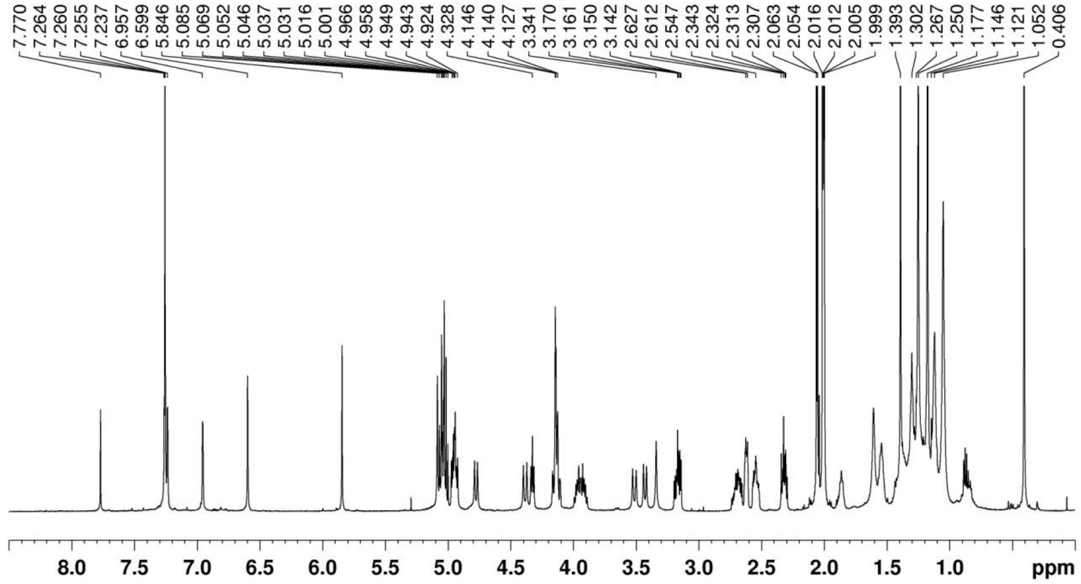

Figure S38. ^{1}H NMR spectrum of compound **14b** ($CDCl_3$, 600 MHz).

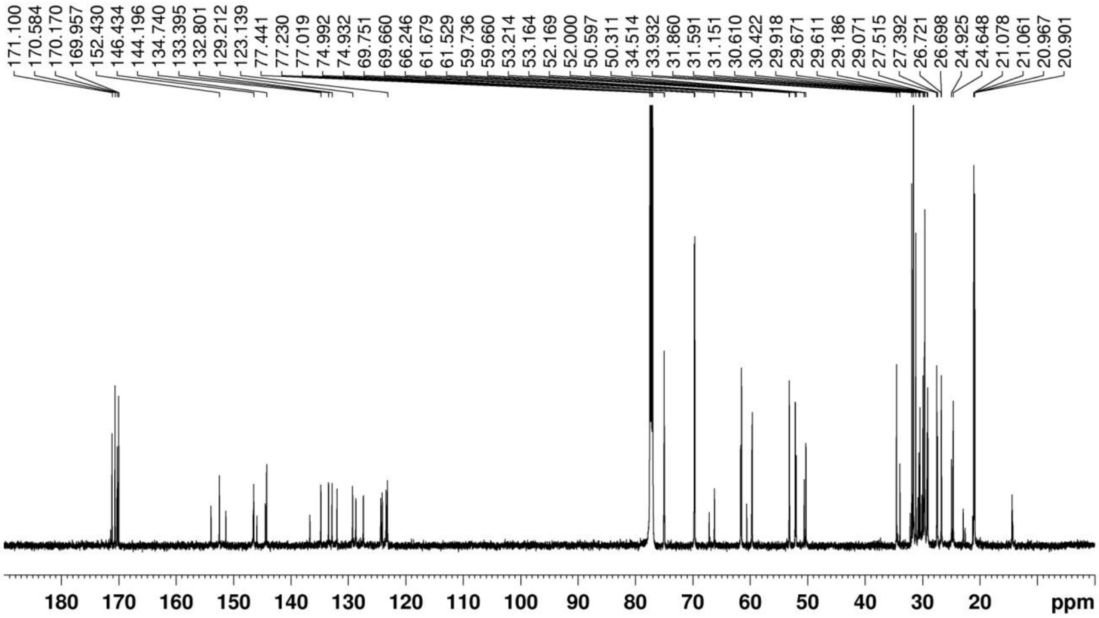

Figure S39. ^{13}C NMR spectrum of compound **14b** ($CDCl_3$, 150 MHz).

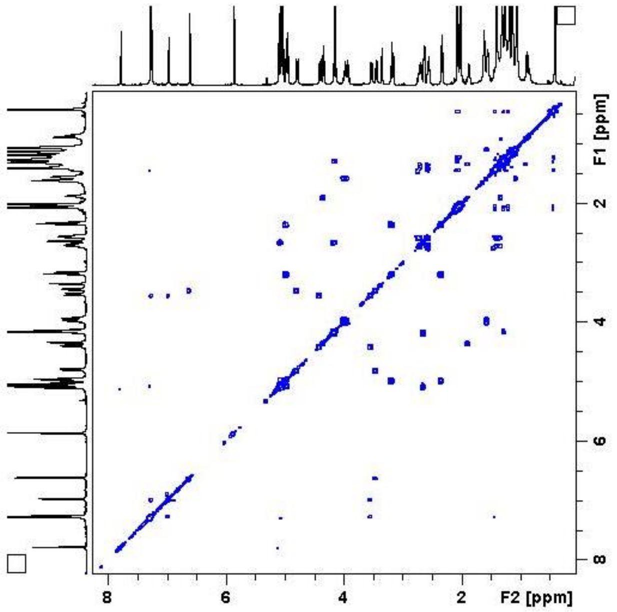

Figure S40. 2D-COSY spectrum of compound **14b** ($CDCl_3$, 600 MHz).

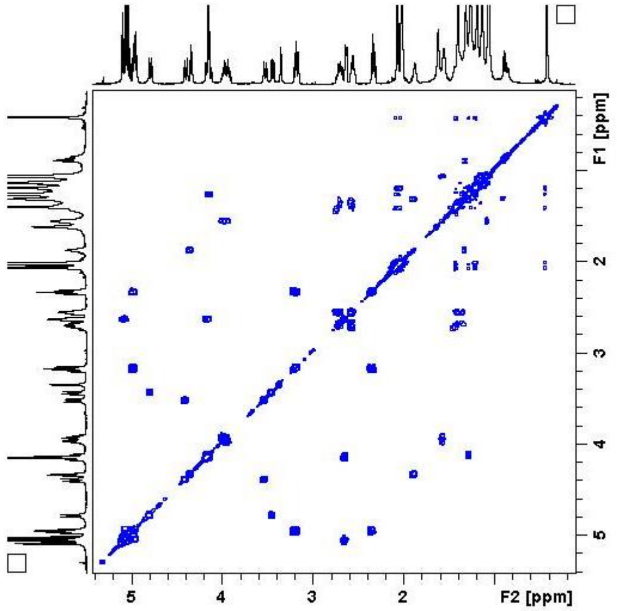

Figure S41. 2D-COSY NMR spectrum of compound **14b** -expansion ($CDCl_3$, 600 MHz).

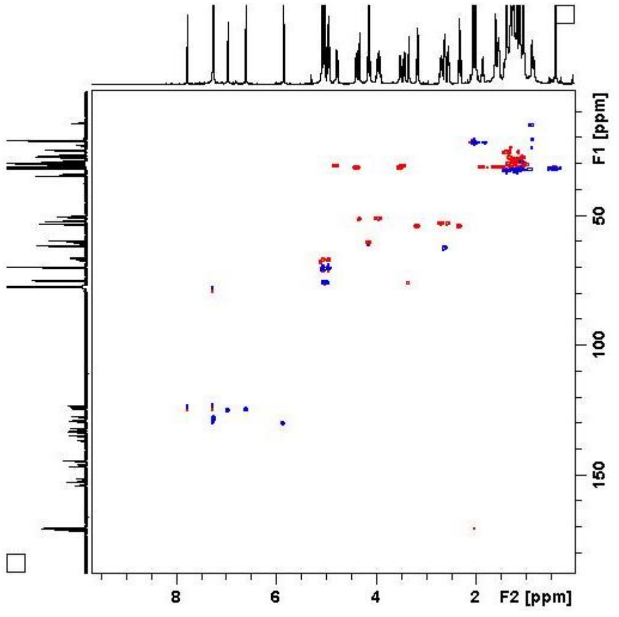

Figure S42. 2D-HSQC NMR spectrum of compound **14b** ($CDCl_3$, 600 MHz).

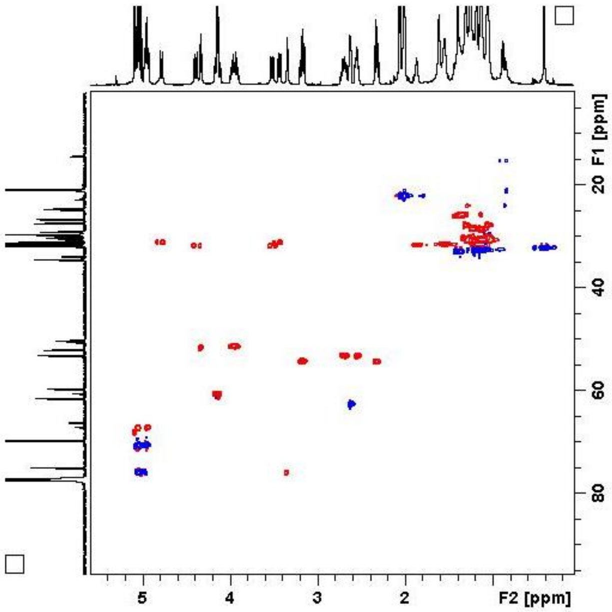

Figure S43. 2D-HSQC NMR spectrum of compound **14b** -expansion ($CDCl_3$, 600 MHz).

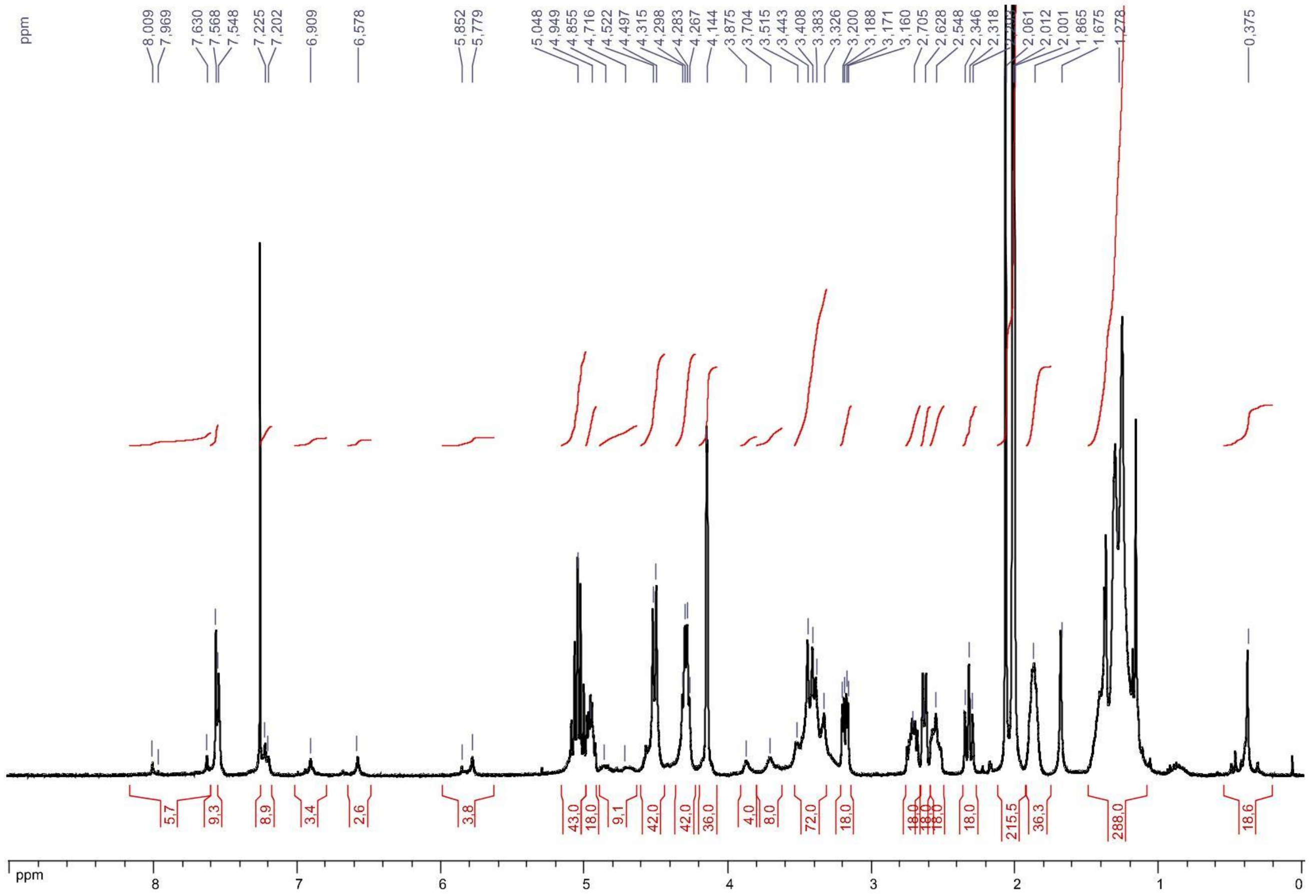

Figure S44. ^{1}H NMR spectrum of compound **14c** ($CDCl_3$, 400 MHz).

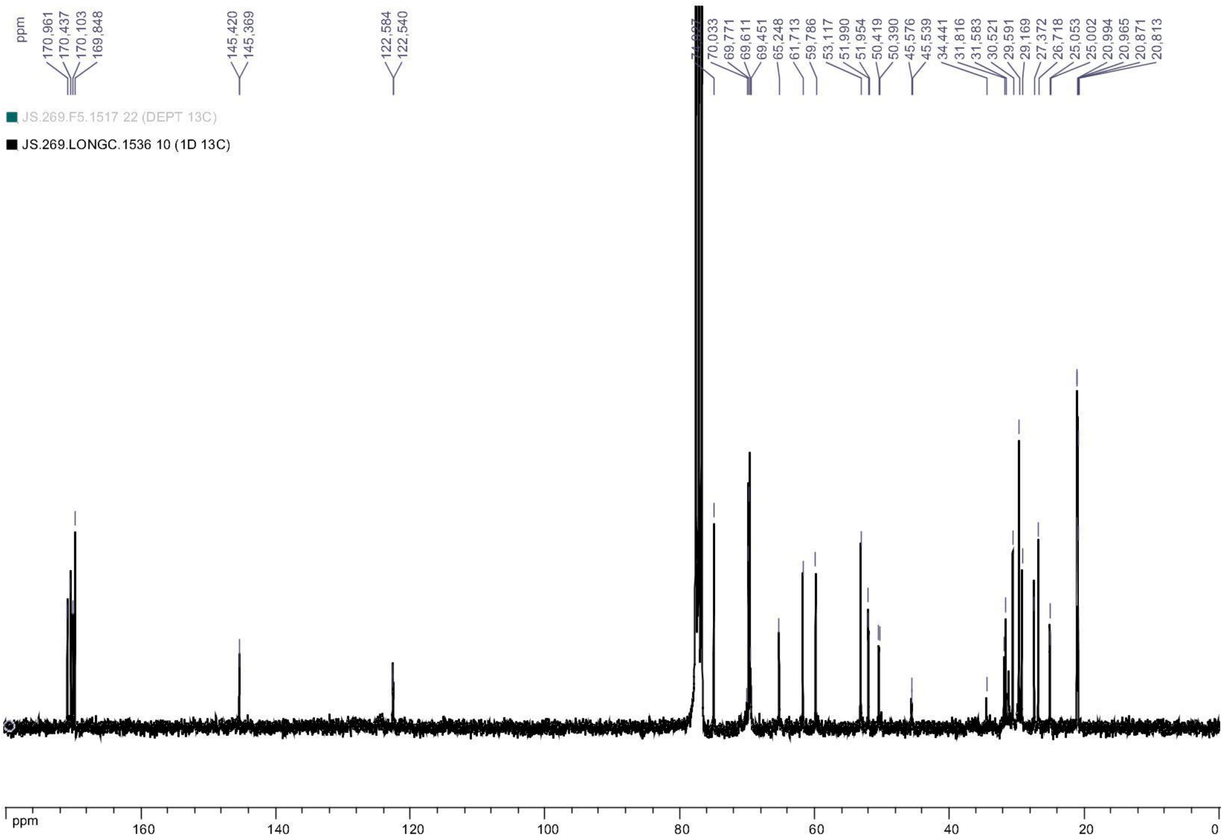

Figure S45. ^{13}C NMR spectrum of compound **14c** ($CDCl_3$, 75 MHz, 15000 scans).

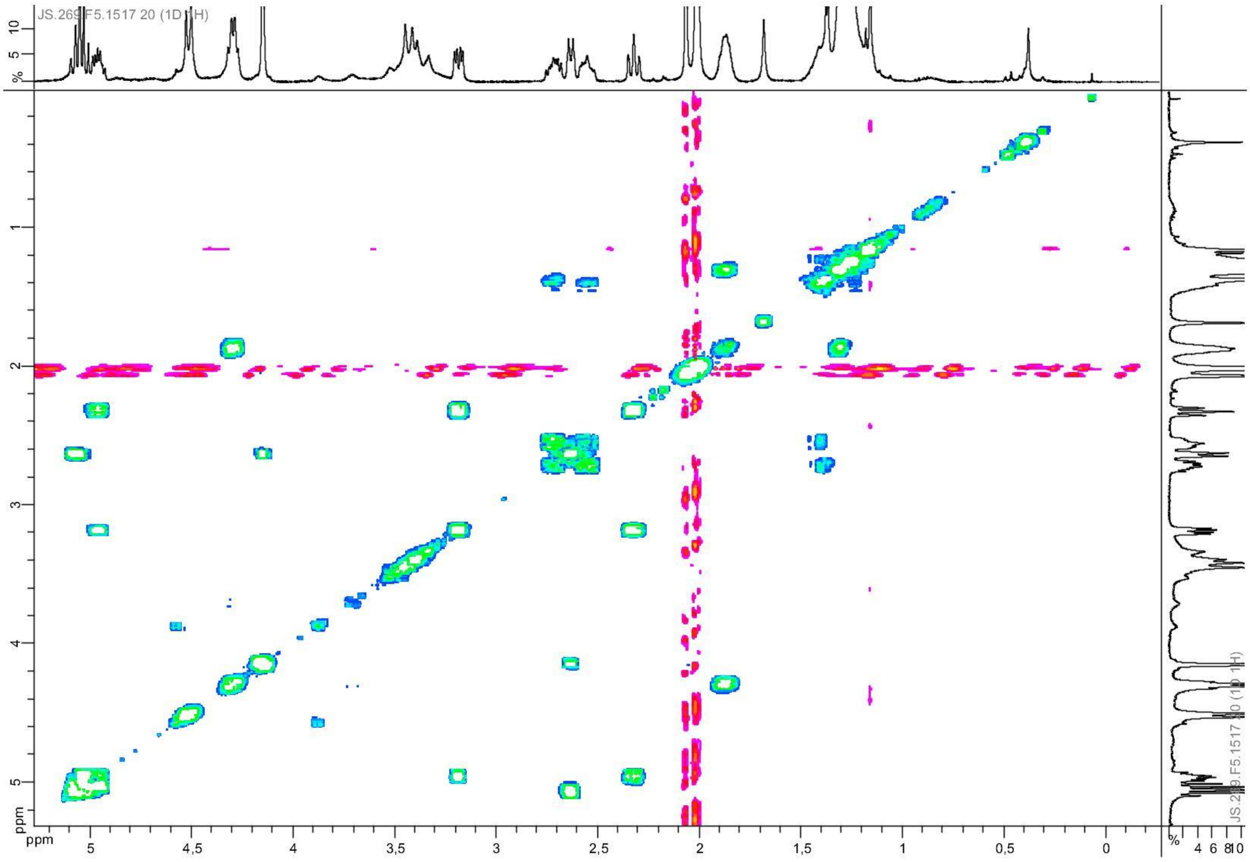

Figure S46. 2D-COSY spectrum of compound **14c** ($CDCl_3$, 400 MHz).

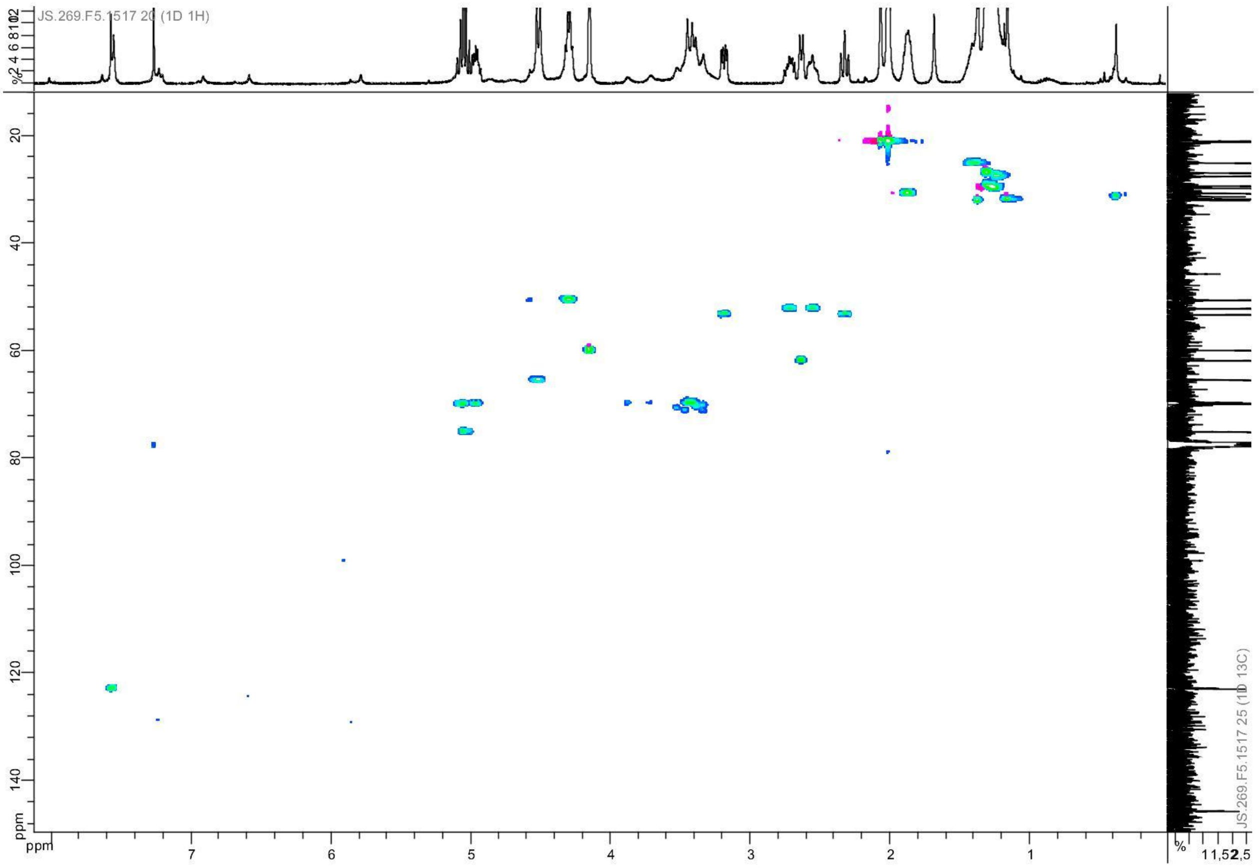

Figure S47. 2D-HSQC NMR spectrum of compound **14c** ($CDCl_3$, 400 MHz).

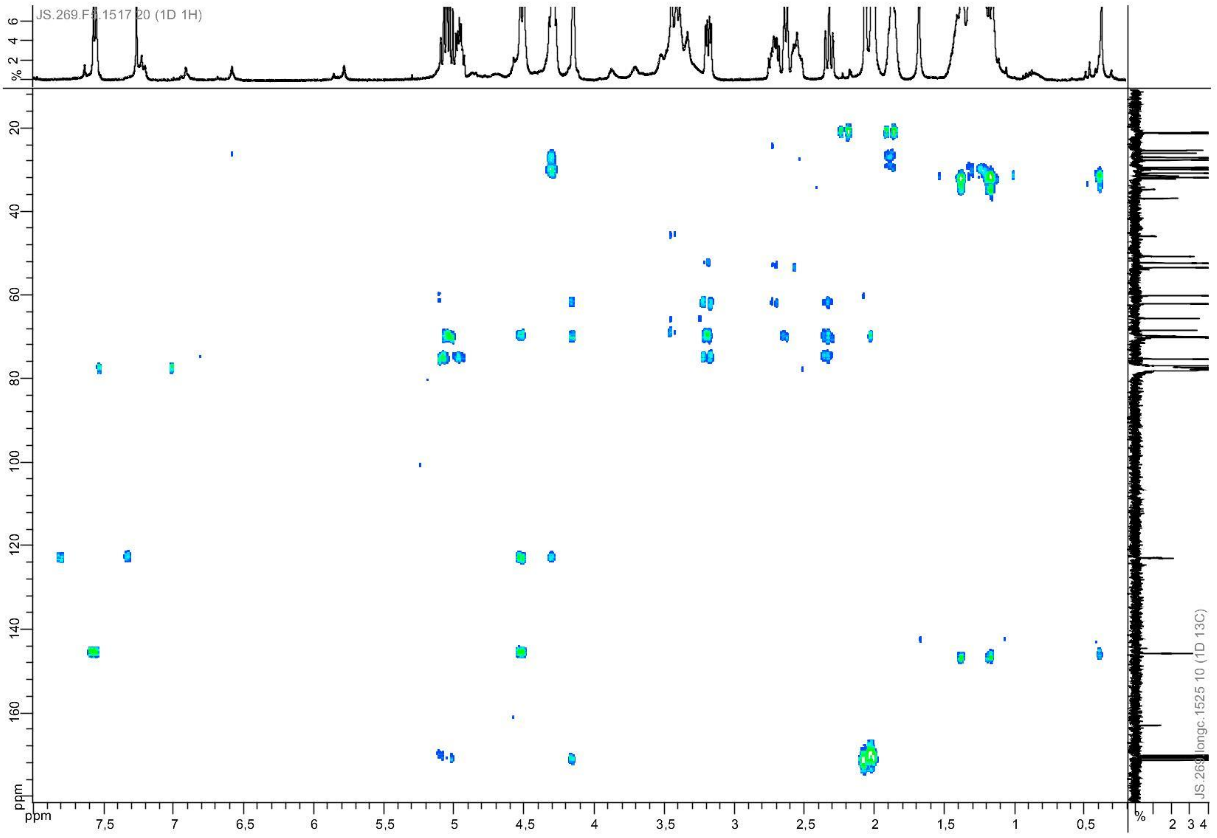

Figure S48. 2D-HMBC NMR spectrum of compound **14c** ($CDCl_3$, 400 MHz).

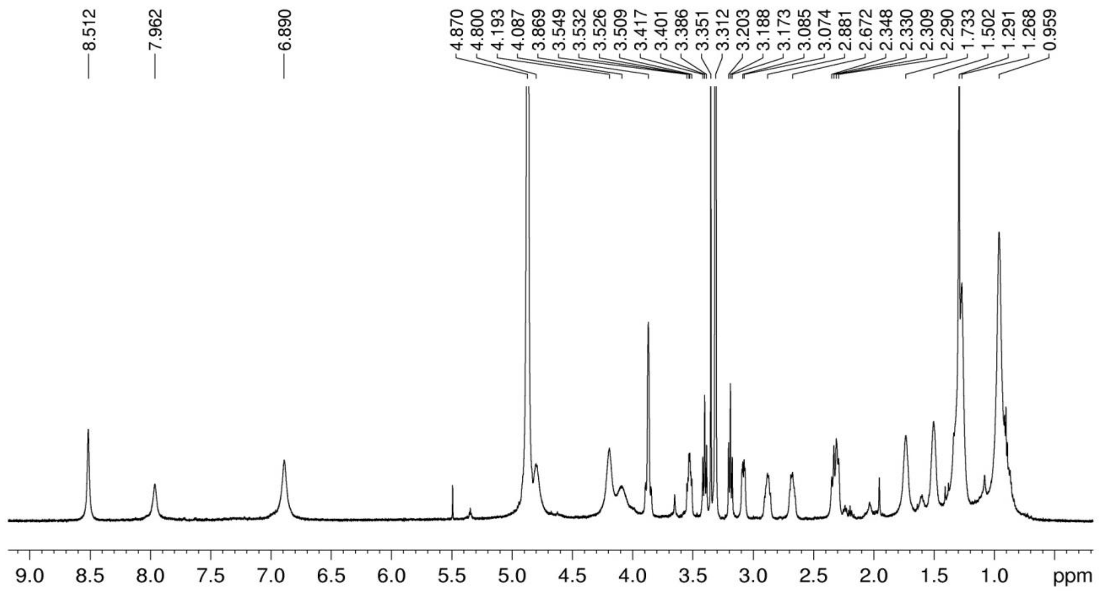

Figure S49. ^{1}H NMR spectrum of compound **6a** (CD_3OD, 600 MHz).

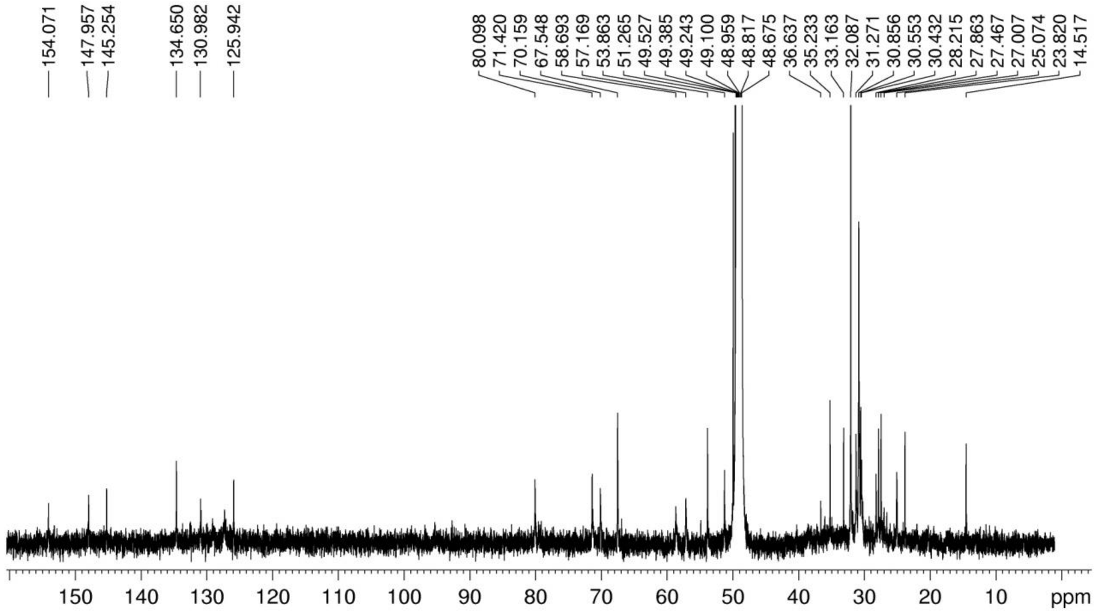

Figure S50. ^{13}C NMR spectrum of compound **6a** (MeOD, 150 MHz).

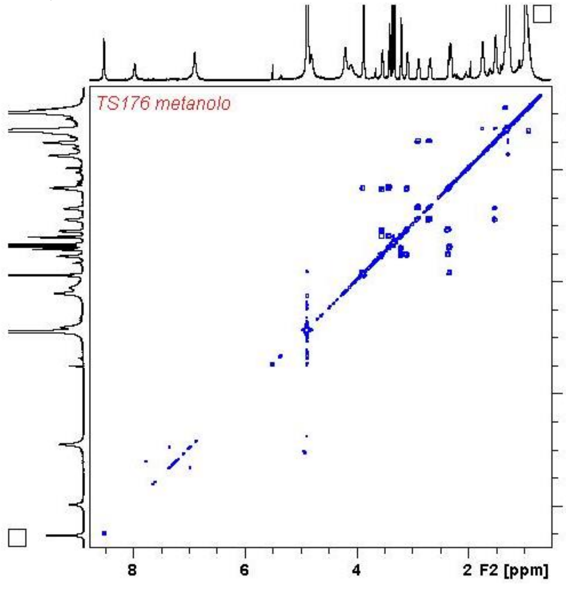

Figure S51. 2D-COSY spectrum of compound **6a** (CD_3OD, 600 MHz).

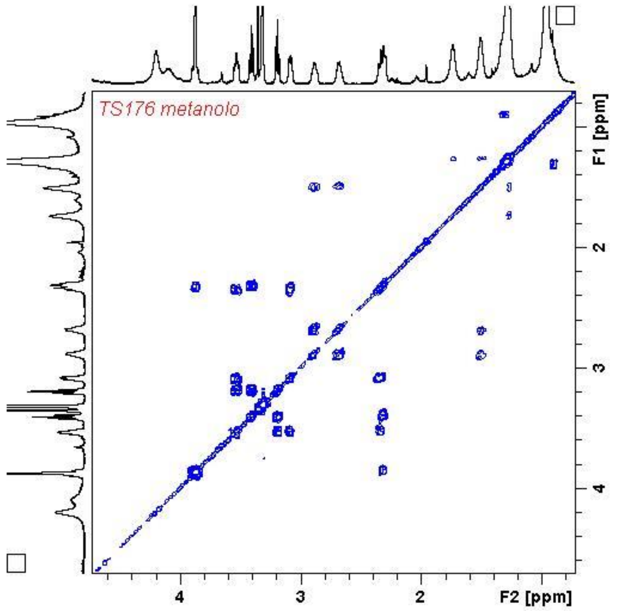

Figure S52. 2D-COSY NMR spectrum of compound **6a** -expansion (CD_3OD, 600 MHz).

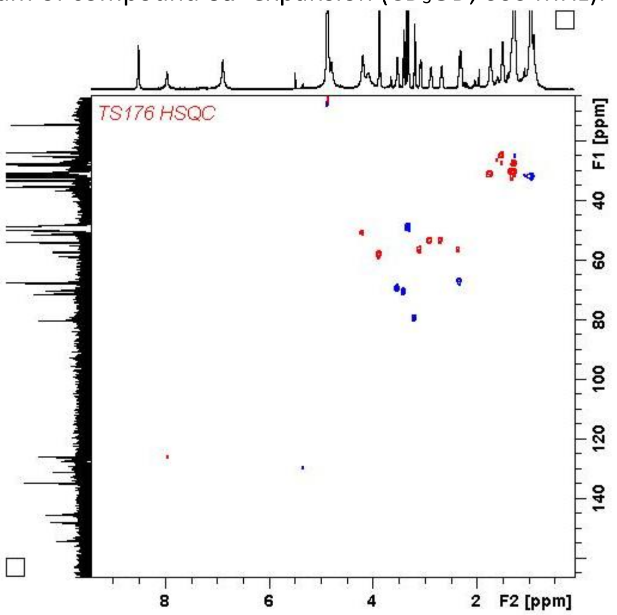

Figure S53. 2D-HSQC NMR spectrum of compound **6a** (CD_3OD, 600 MHz).

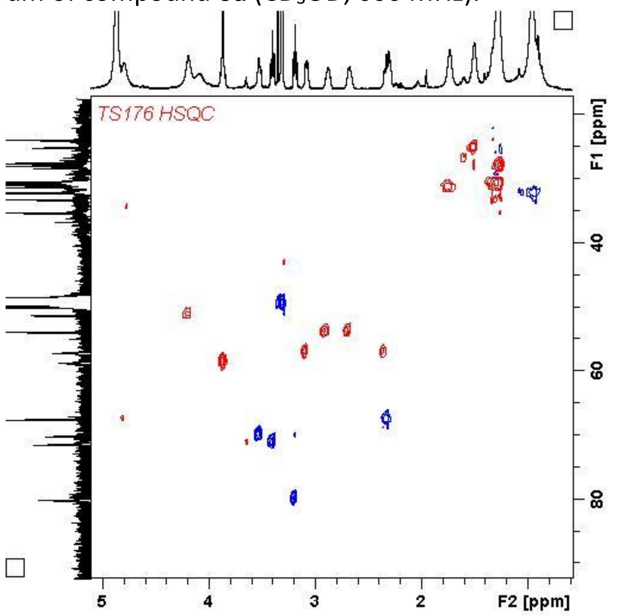

Figure S54. 2D-HSQC NMR spectrum of compound **6a** -expansion (CD_3OD, 600 MHz).

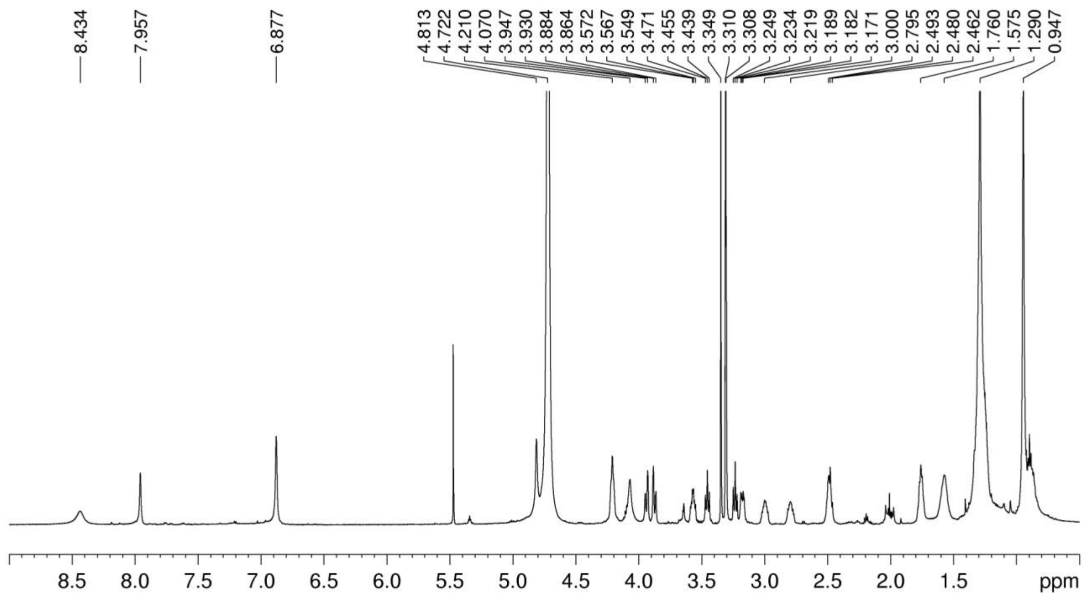

Figure S55. ^{1}H NMR spectrum of compound **6b** (CD_3OD, 600 MHz, 313 K).

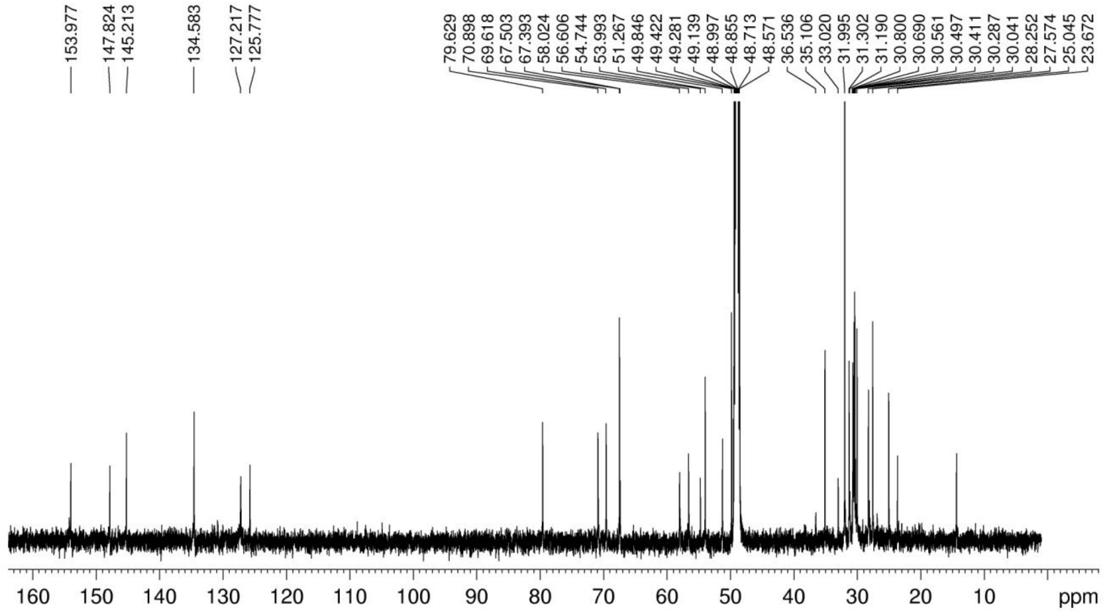

Figure S56. ^{13}C NMR spectrum of compound **6b** (CD_3OD, 150 MHz, 313 K).

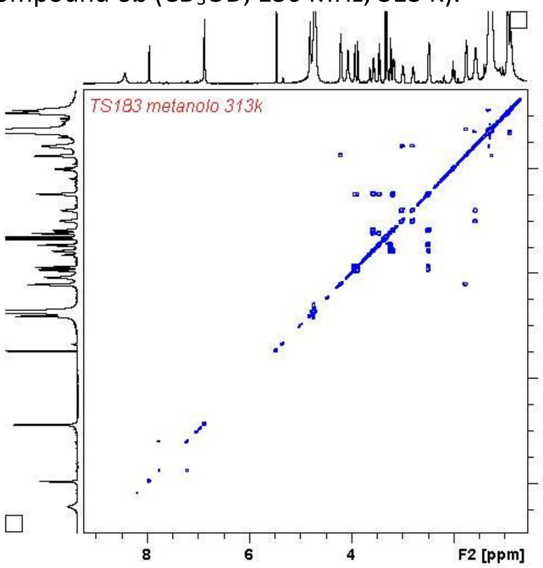

Figure S57. 2D-COSY spectrum of compound **6b** (CD_3OD, 600 MHz, 313 K).

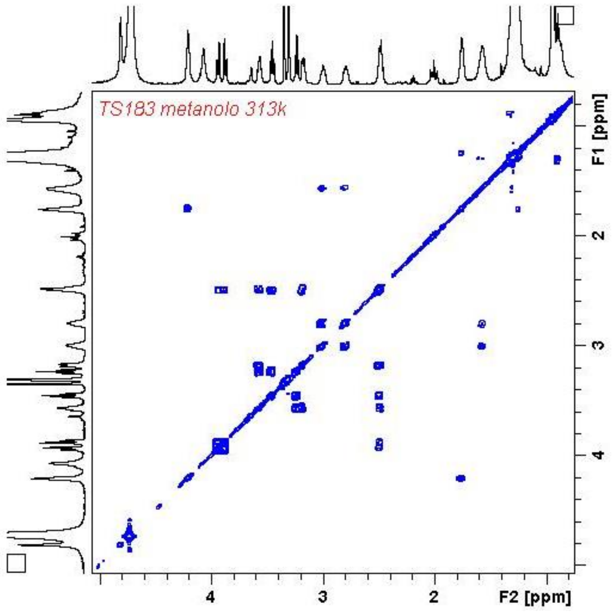

Figure S58. 2D-COSY NMR spectrum of compound **6b** -expansion (CD_3OD, 600 MHz, 313 K).

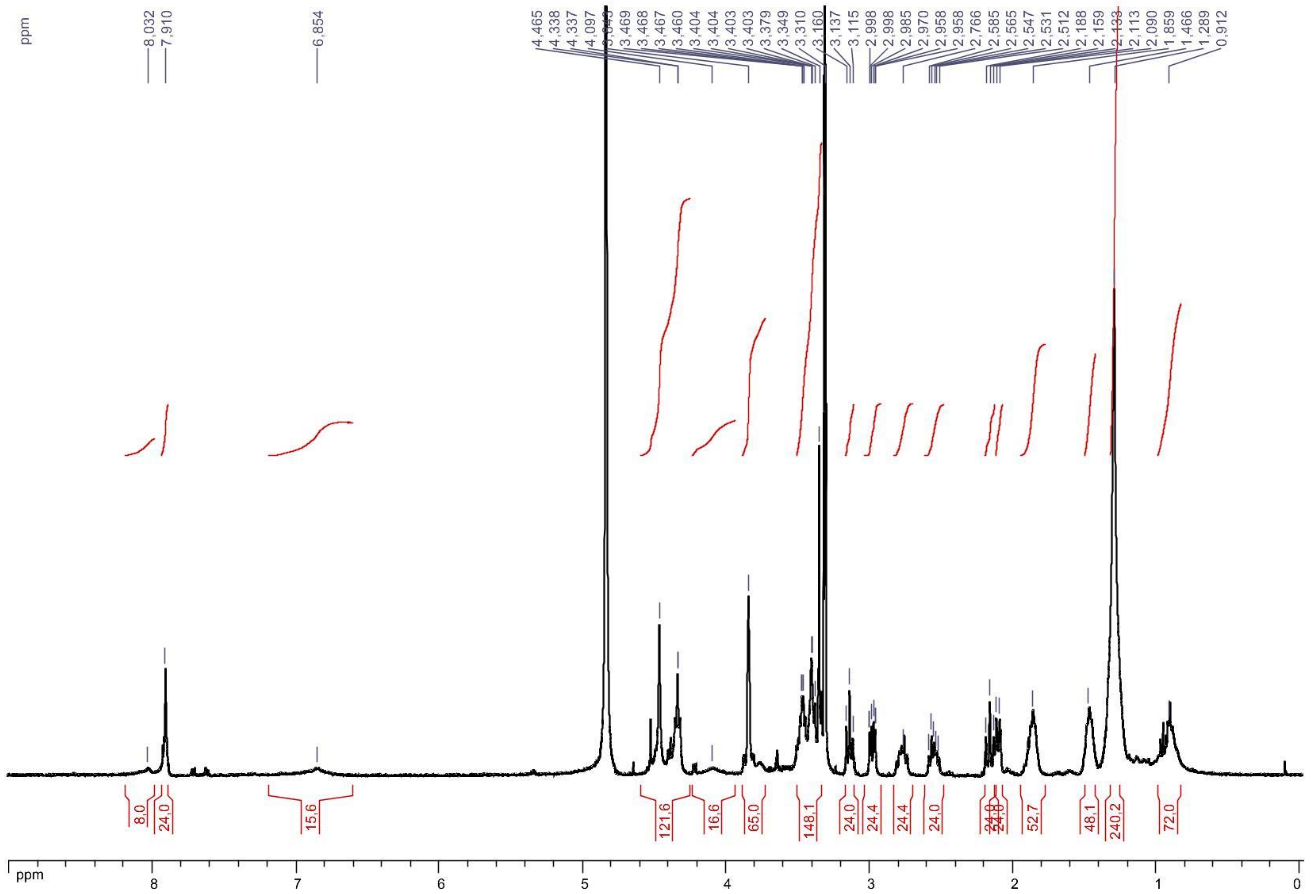

Figure S59. ^1H NMR spectrum of compound **6c** (CD_3OD, 400 MHz).

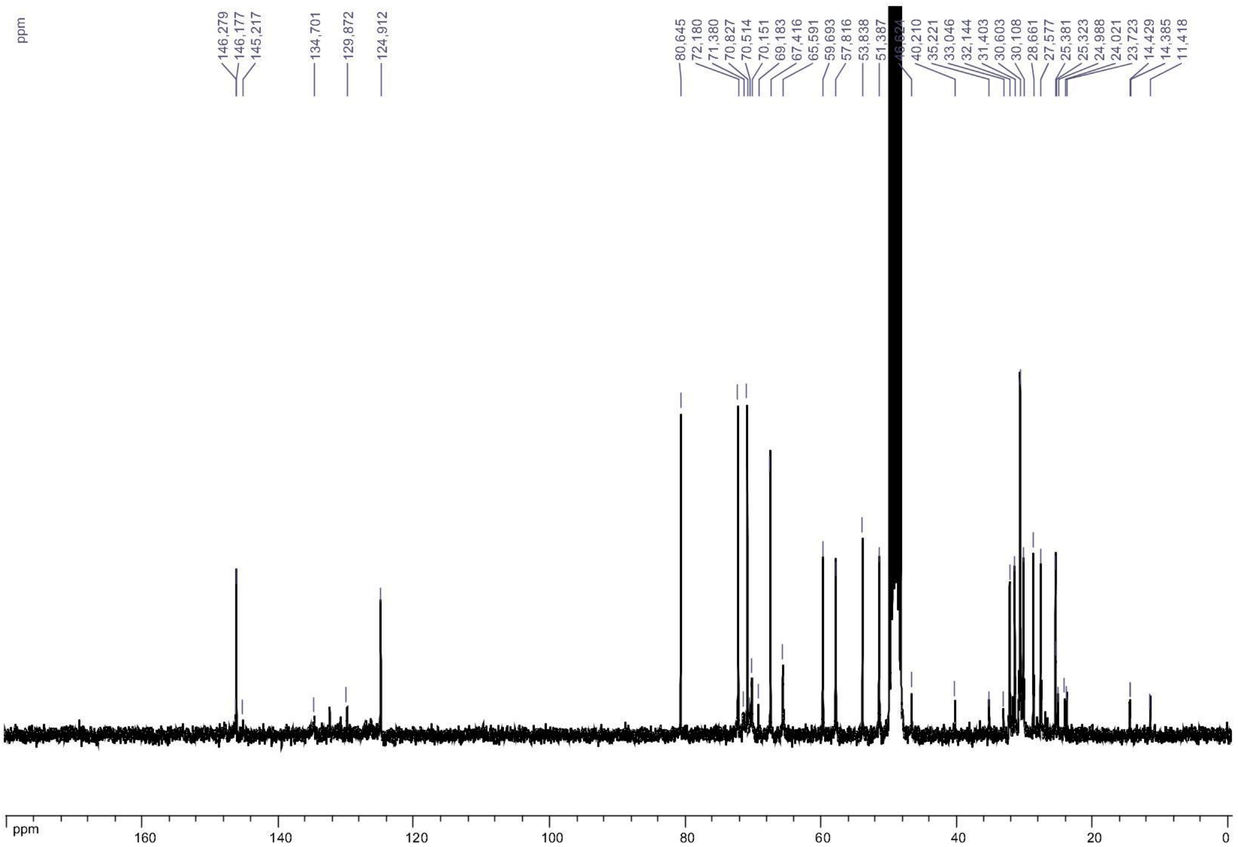

Figure S60. ^{13}C NMR spectrum of compound **6c** (CD_3OD, 75 MHz, 40000 scans).

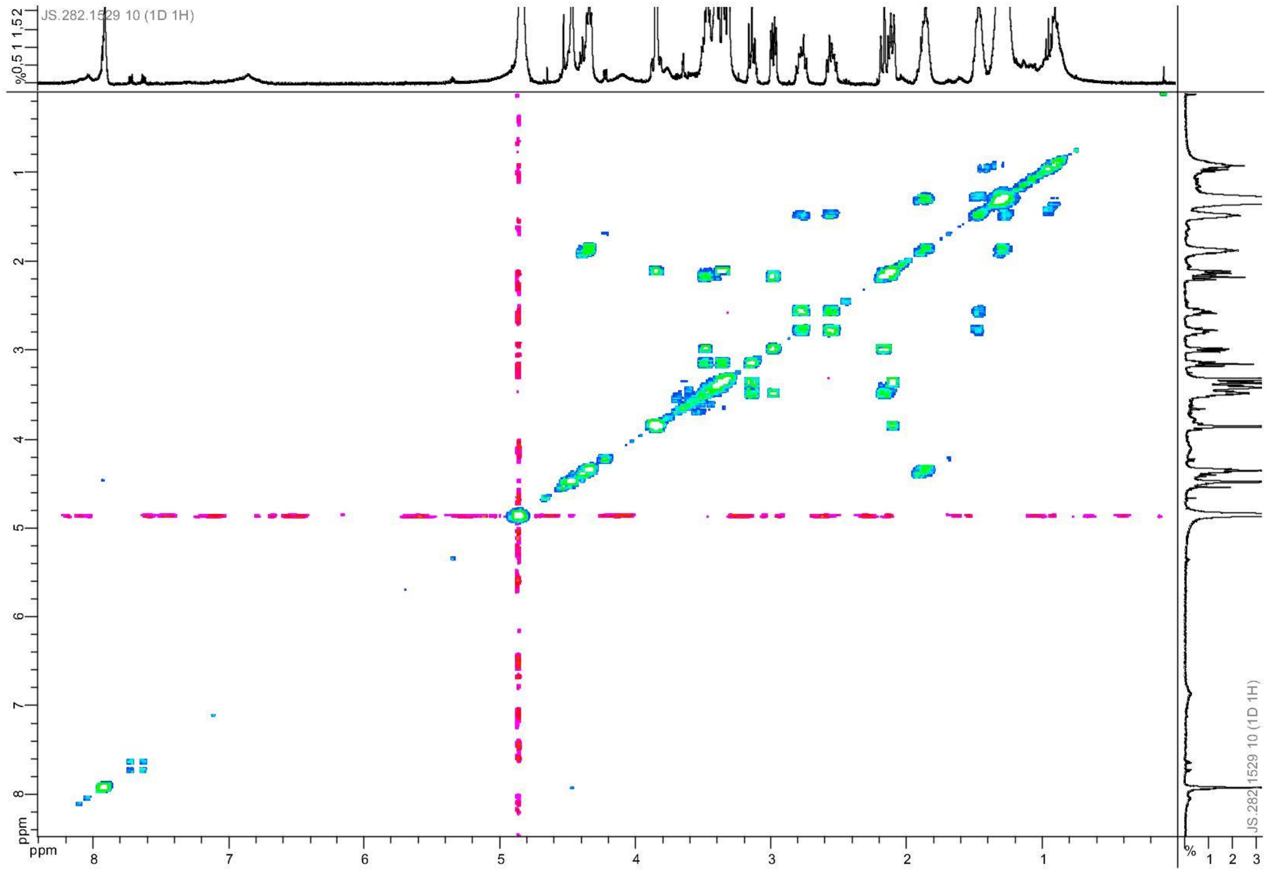

Figure S61. 2D-COSY spectrum of compound **6c** (CD_3OD, 400 MHz).

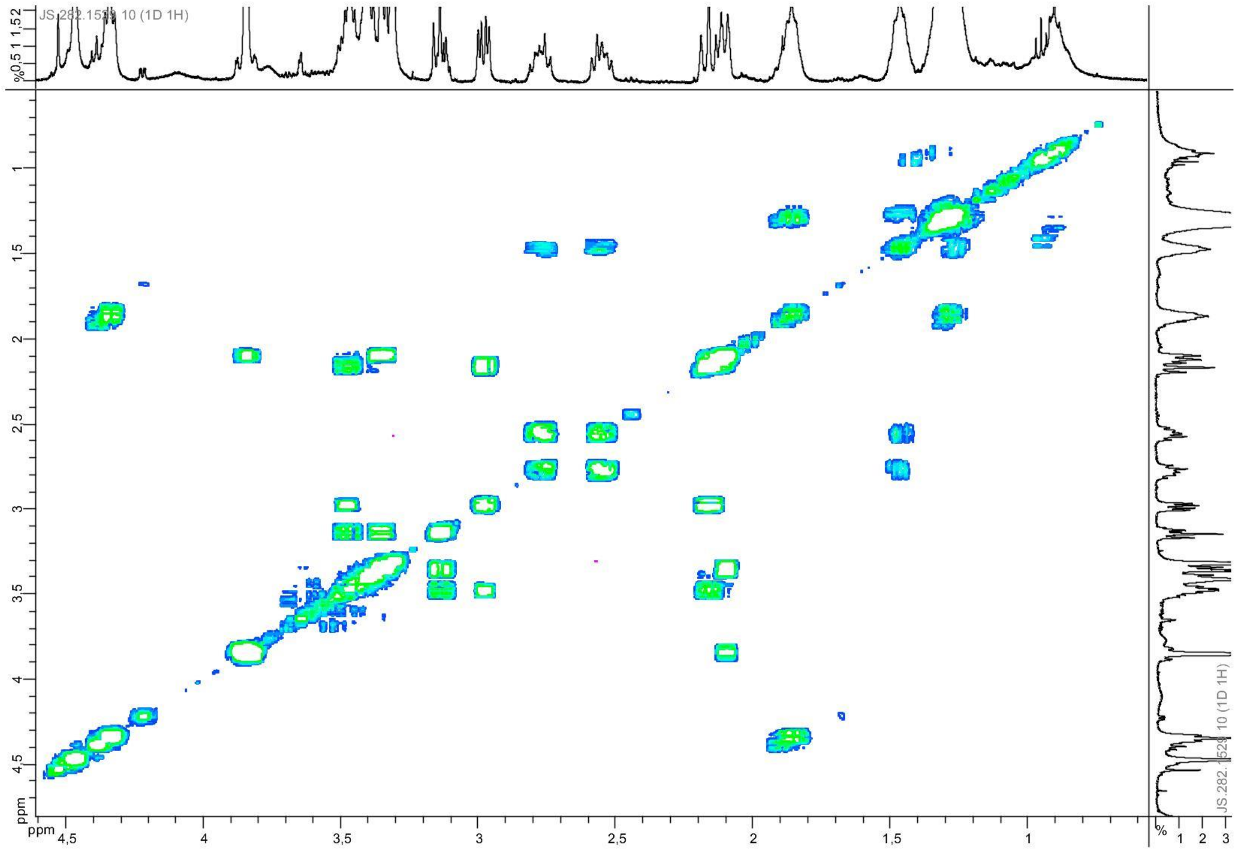

Figure S62. 2D-COSY NMR spectrum of compound **6c** -expansion (CD_3OD, 400 MHz).

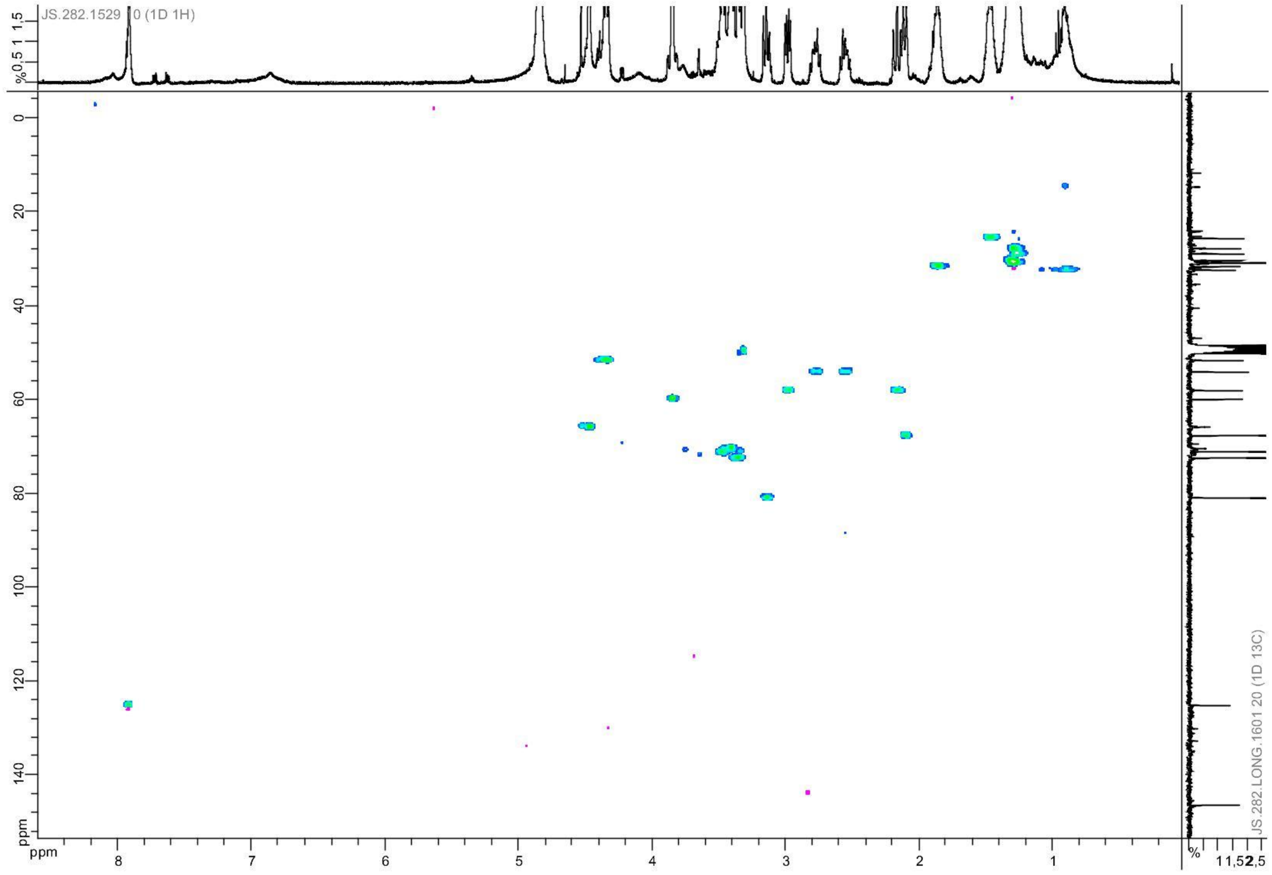

Figure S63. 2D-HSQC NMR spectrum of compound **6c** (CD_3OD, 400 MHz).

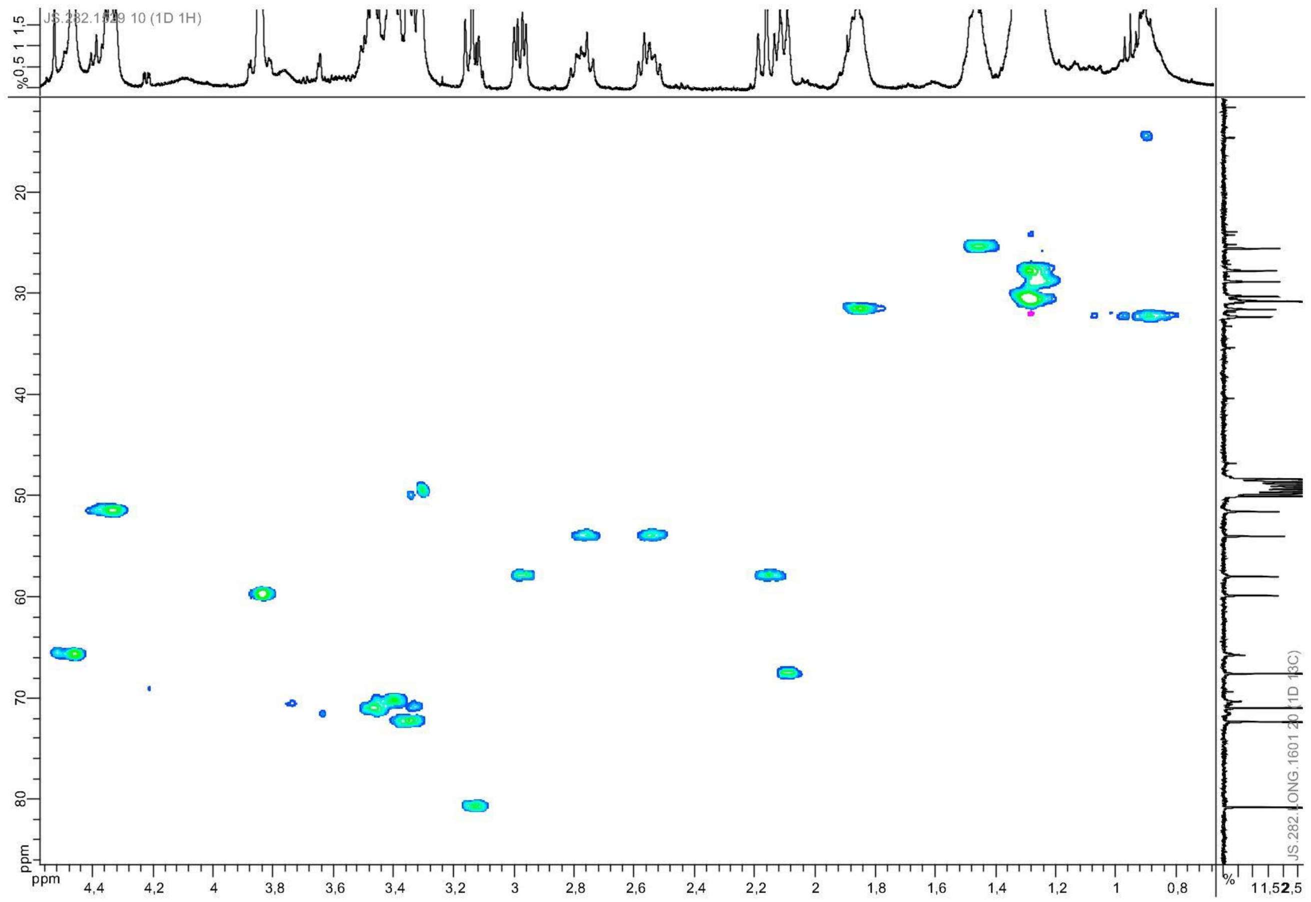

Figure S64. 2D-HSQC NMR spectrum of compound **6c** -expansion (CD_3OD, 400 MHz).

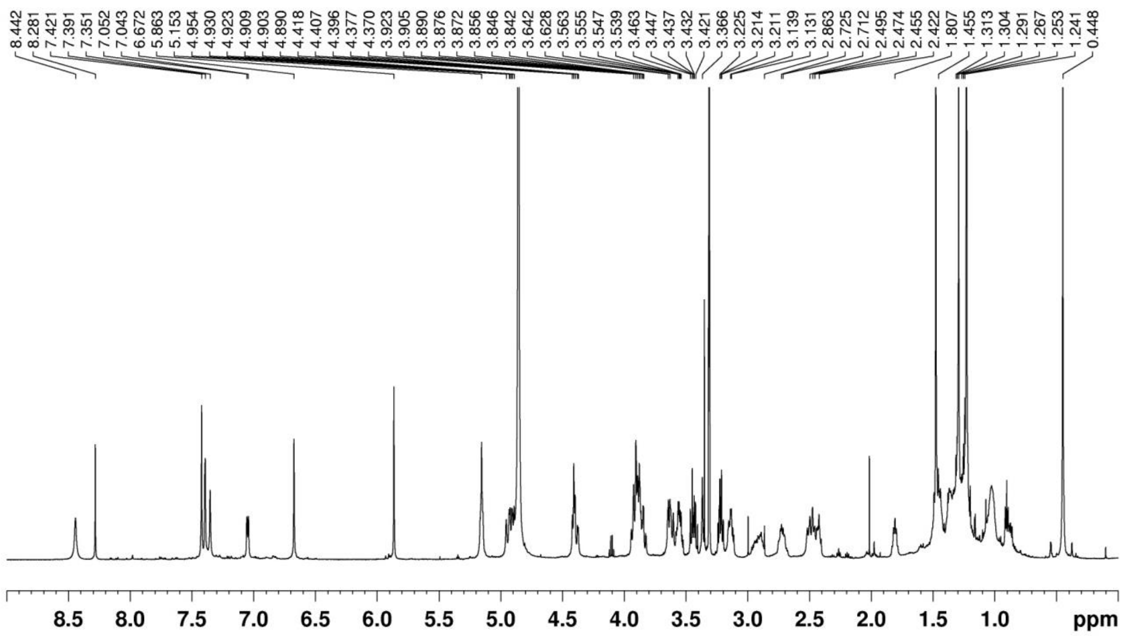

Figure S65. ^1H NMR spectrum of compound **7a** (CD_3OD, 600 MHz).

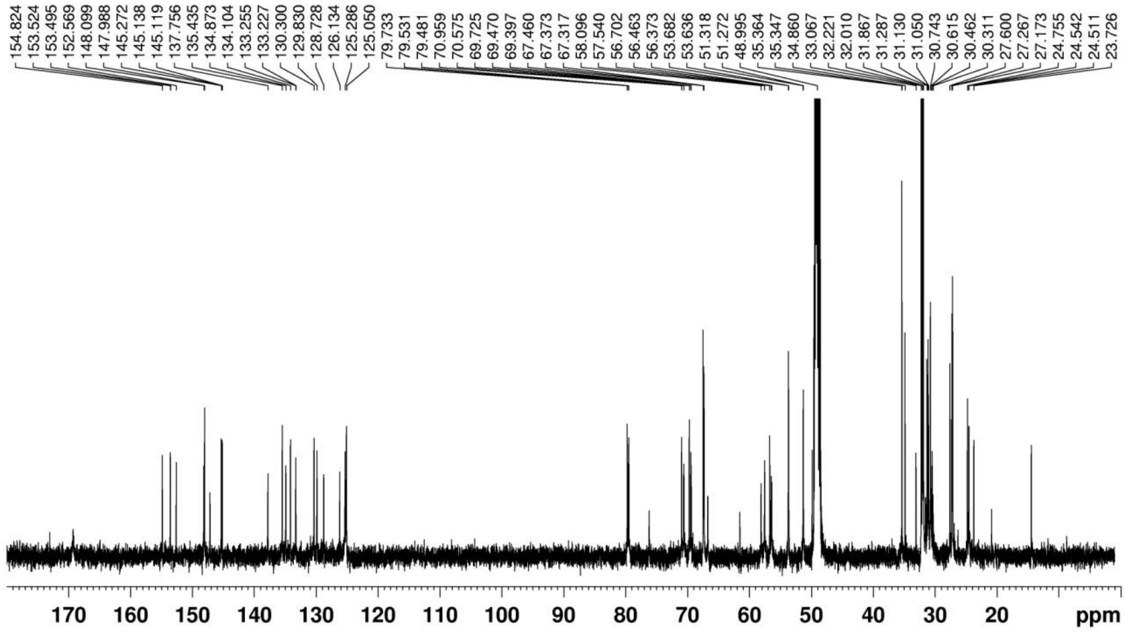

Figure S66. ^{13}C NMR spectrum of compound **7a** (CD_3OD, 150 MHz).

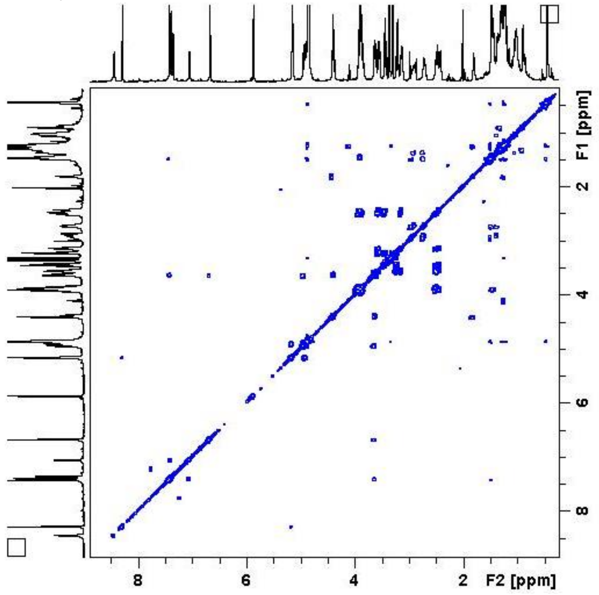

Figure S67. 2D-COSY spectrum of compound **7a** (CD_3OD, 600 MHz).

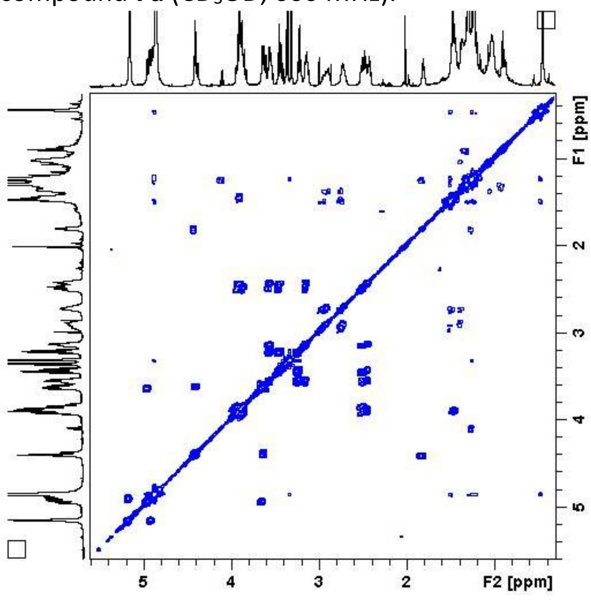

Figure S68. 2D-COSY NMR spectrum of compound **7a** -expansion (CD_3OD, 600 MHz).

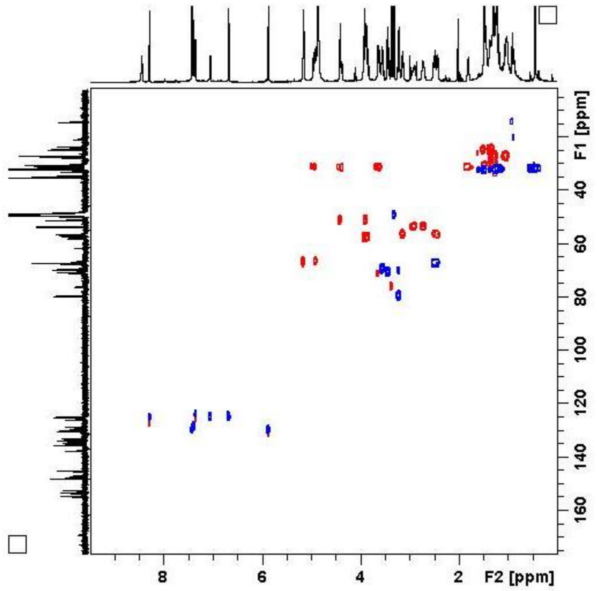

Figure S69. 2D-HSQC NMR spectrum of compound **7a** (CD_3OD, 600 MHz).

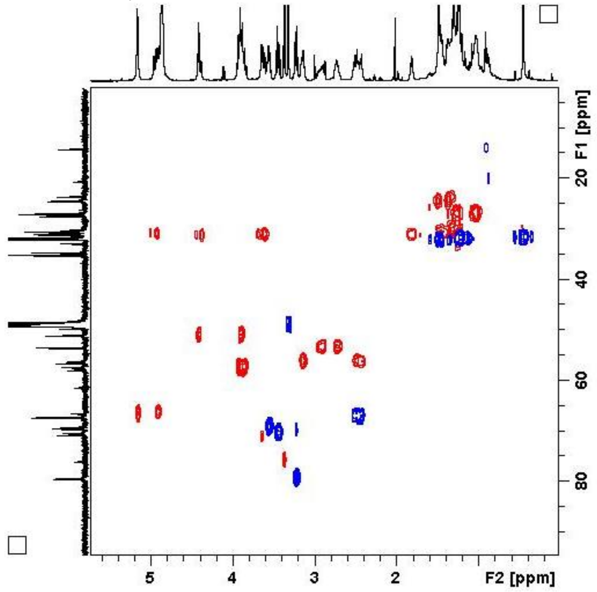

Figure S70. 2D-HSQC NMR spectrum of compound **7a** -expansion (CD_3OD, 600 MHz).

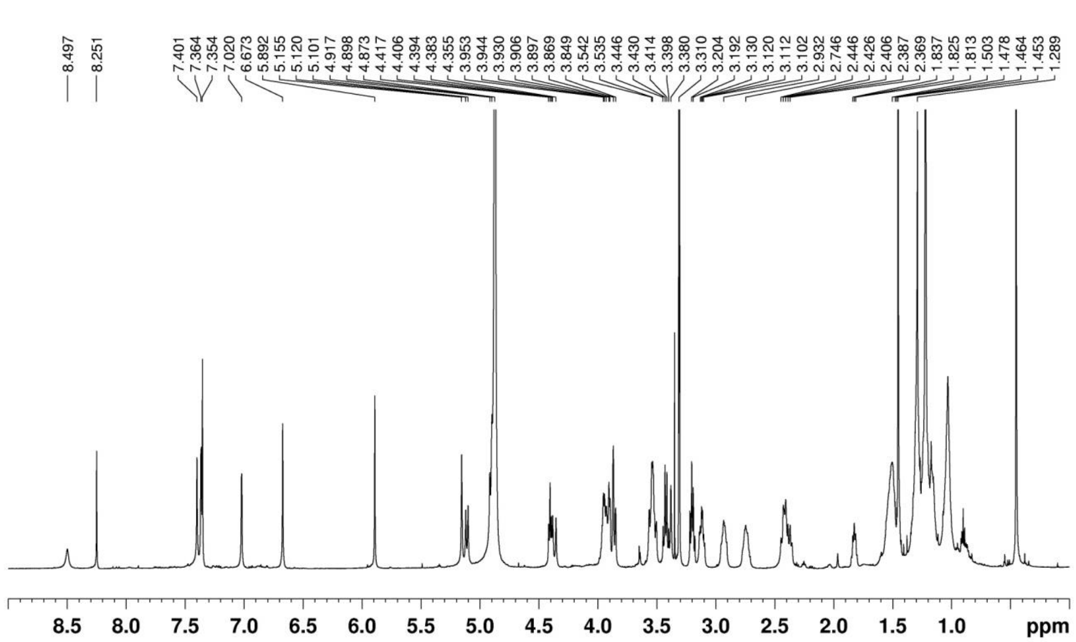

Figure S71. ^{1}H NMR spectrum of compound **7b** (CD_3OD, 600 MHz).

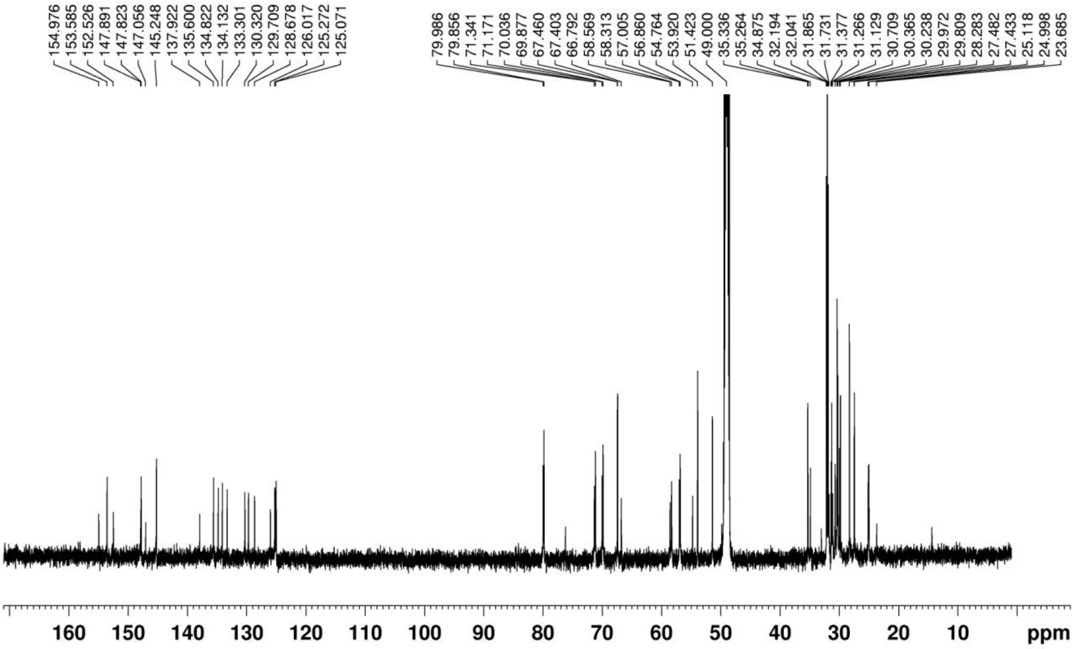

Figure S72. ^{13}C NMR spectrum of compound **7b** (CD_3OD, 150 MHz).

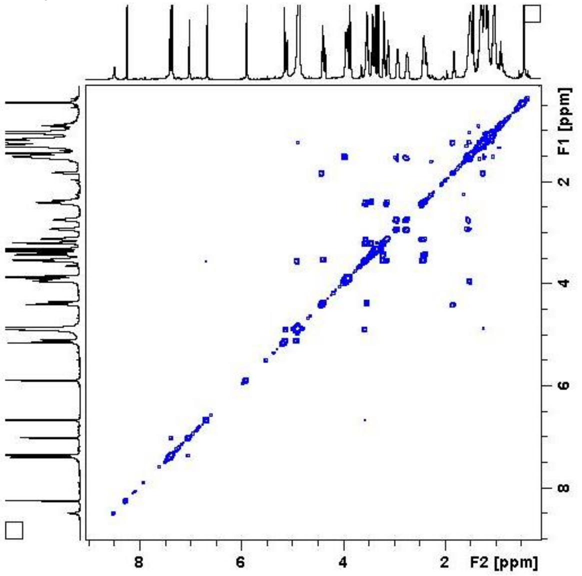

Figure S73. 2D-COSY spectrum of compound **7b** (CD_3OD, 600 MHz).

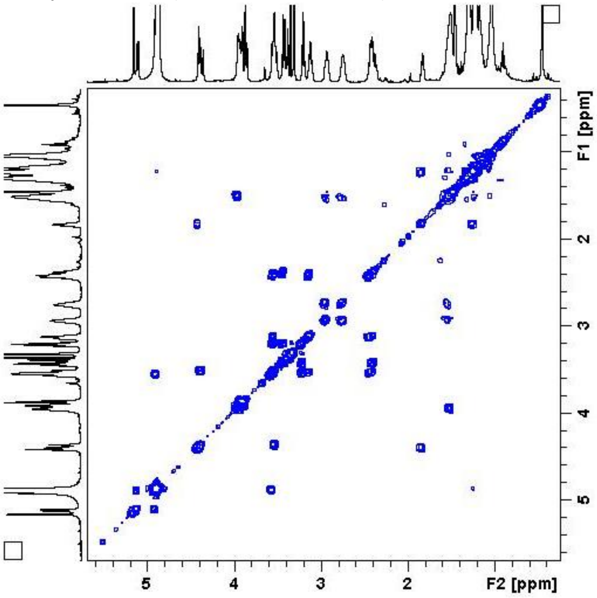

Figure S74. 2D-COSY NMR spectrum of compound **7b** -expansion (CD_3OD, 600 MHz).

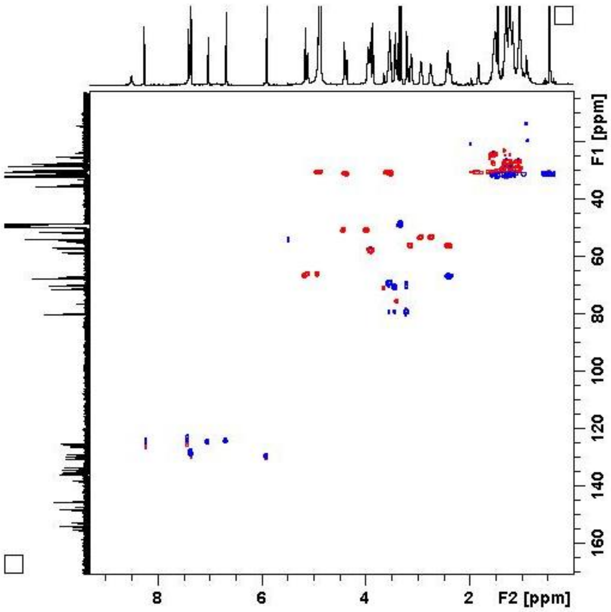

Figure S75. 2D-HSQC NMR spectrum of compound **7b** (CD_3OD, 600 MHz).

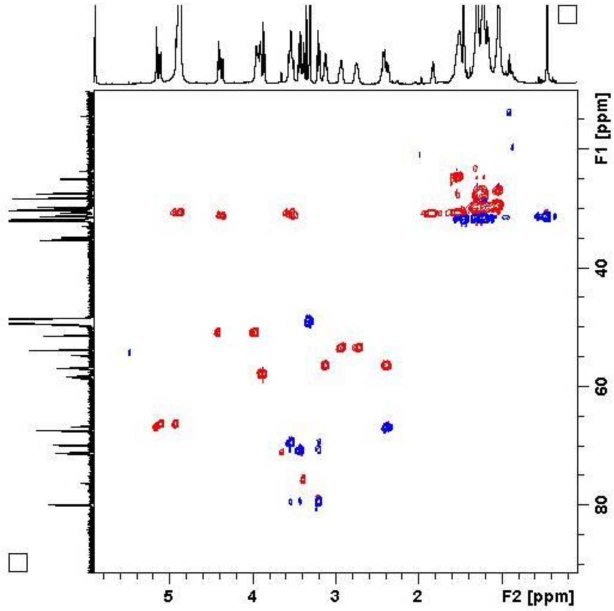

Figure S76. 2D-HSQC NMR spectrum of compound **7b** -expansion (CD_3OD, 600 MHz).

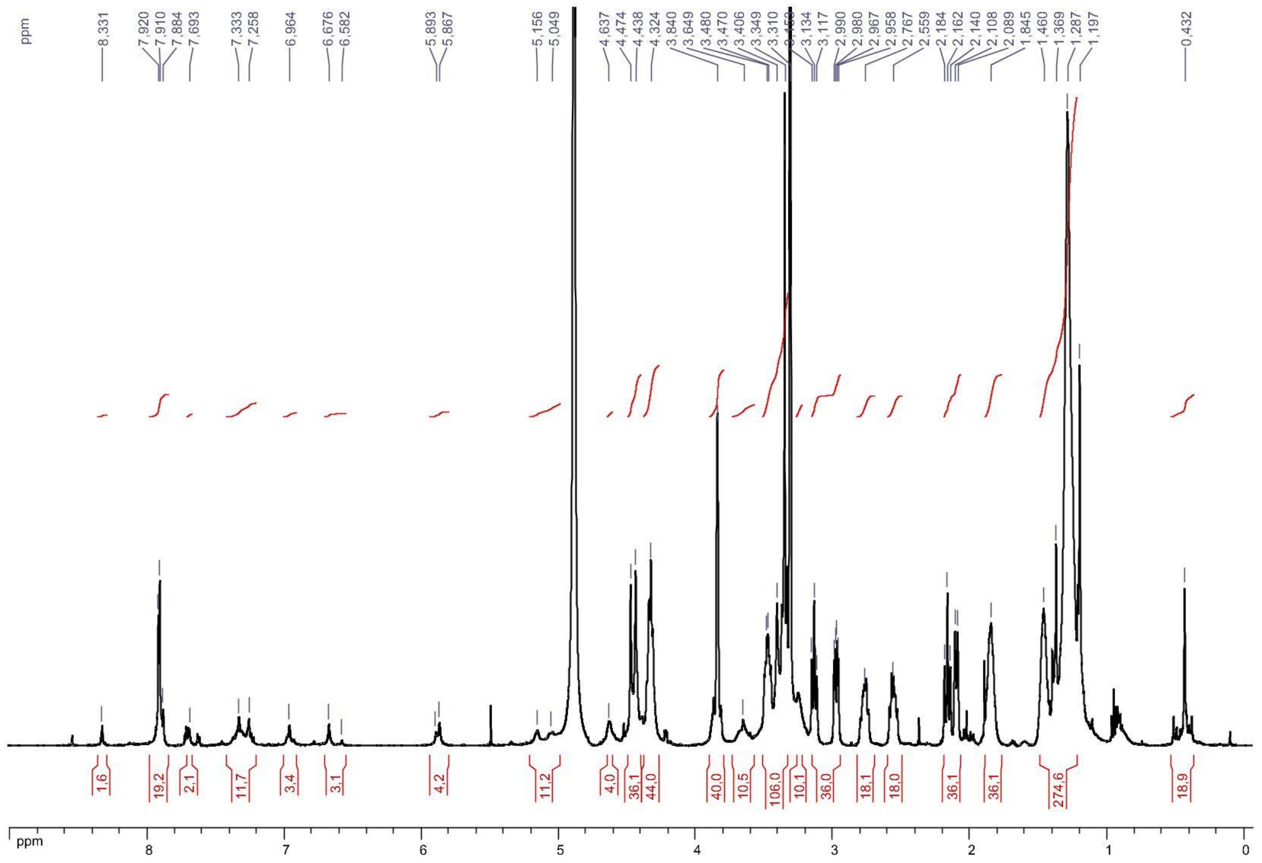

Figure S77. ^{1}H NMR spectrum of compound **7c** (CD_3OD, 400 MHz).

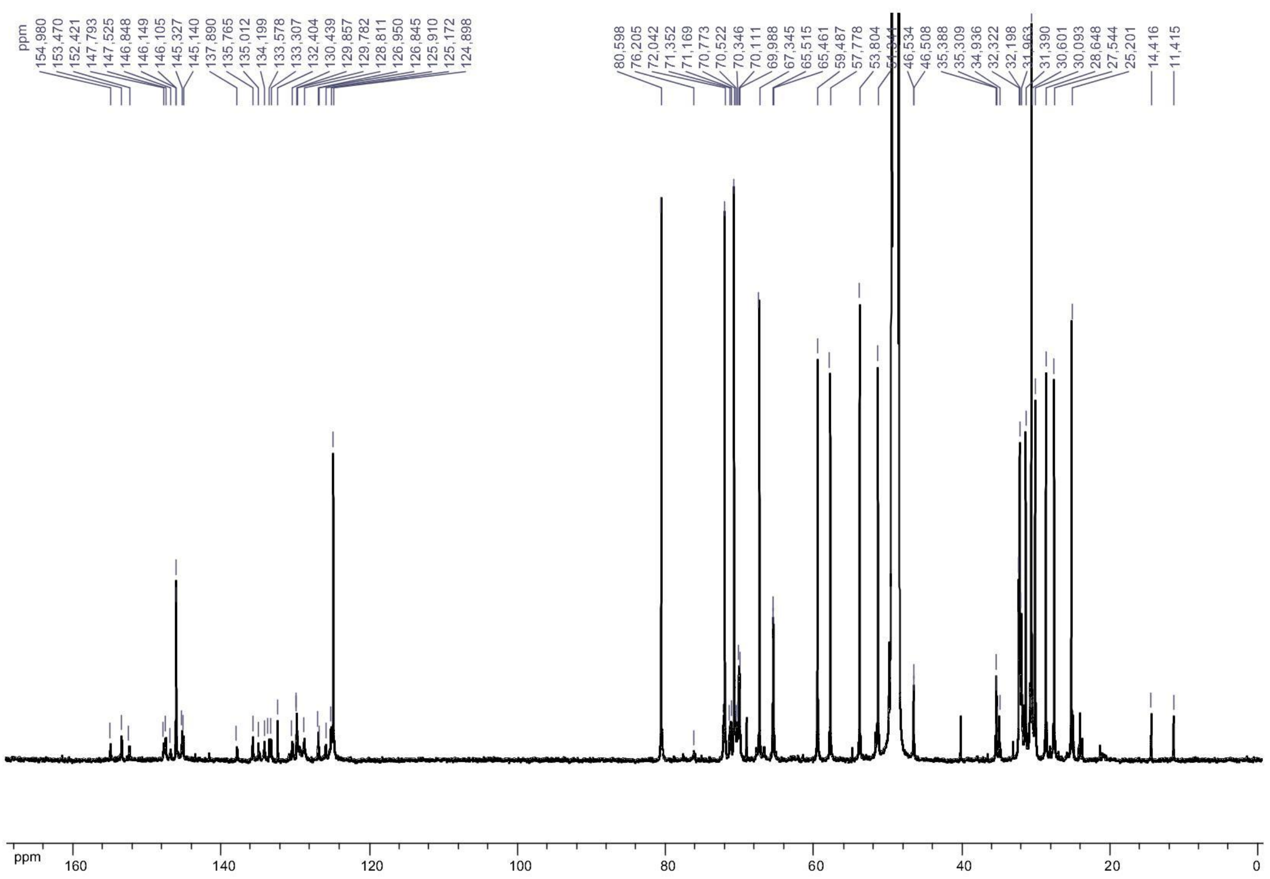

Figure S78. ^{13}C NMR spectrum of compound **7c** (CD_3OD, 125 MHz, 20480 scans).

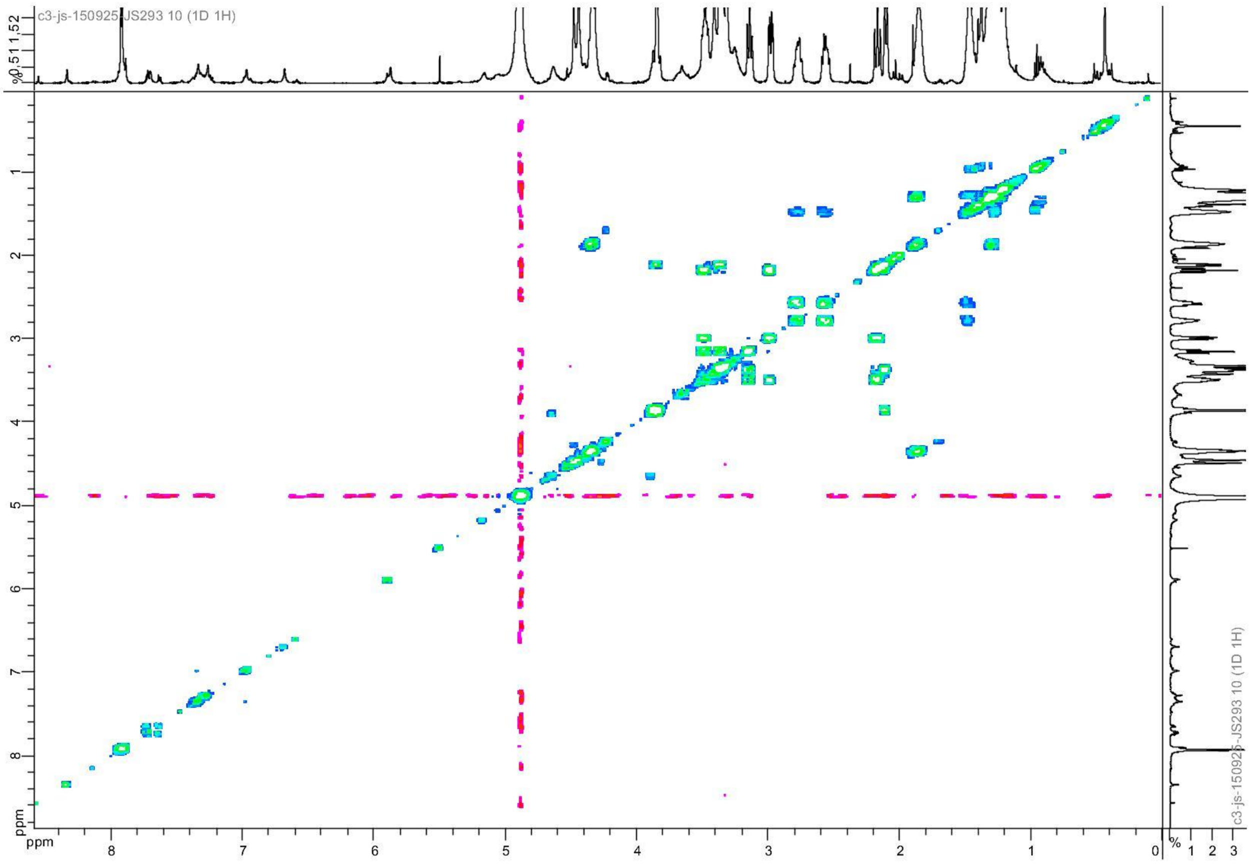

Figure S79. 2D-COSY spectrum of compound **7c** (CD_3OD, 400 MHz).

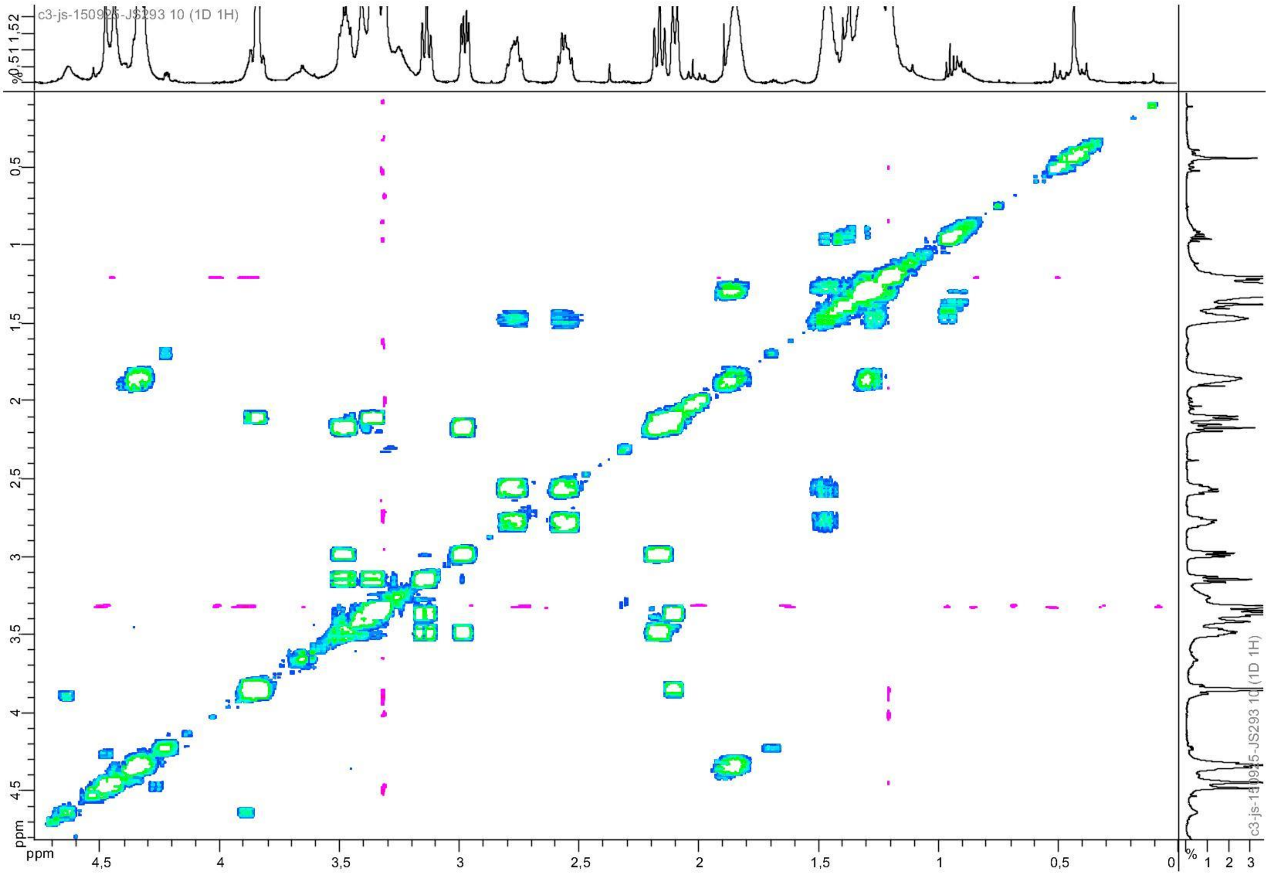

Figure S80. 2D-COSY NMR spectrum of compound **7c** -expansion (CD_3OD, 400 MHz).

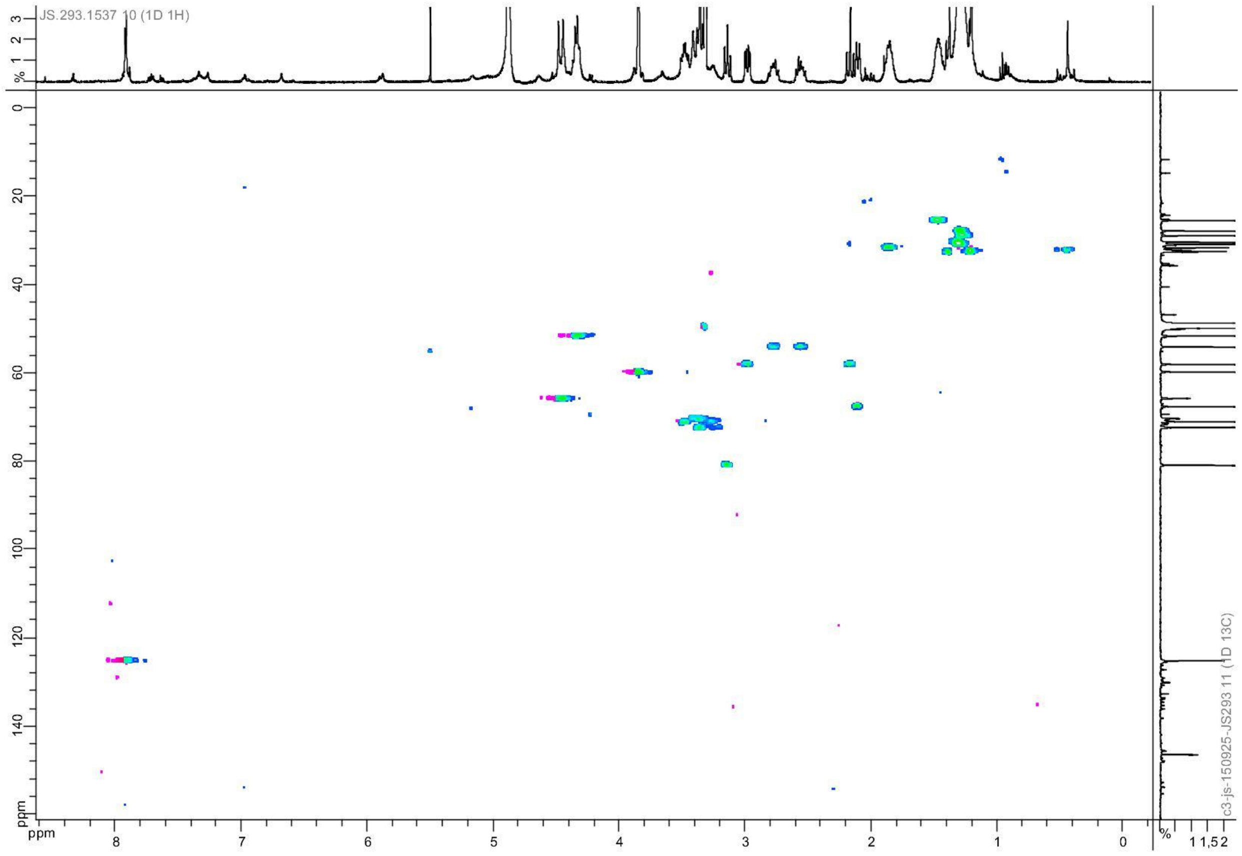

Figure S81. 2D-HSQC NMR spectrum of compound **7c** (CD_3OD, 400 MHz).

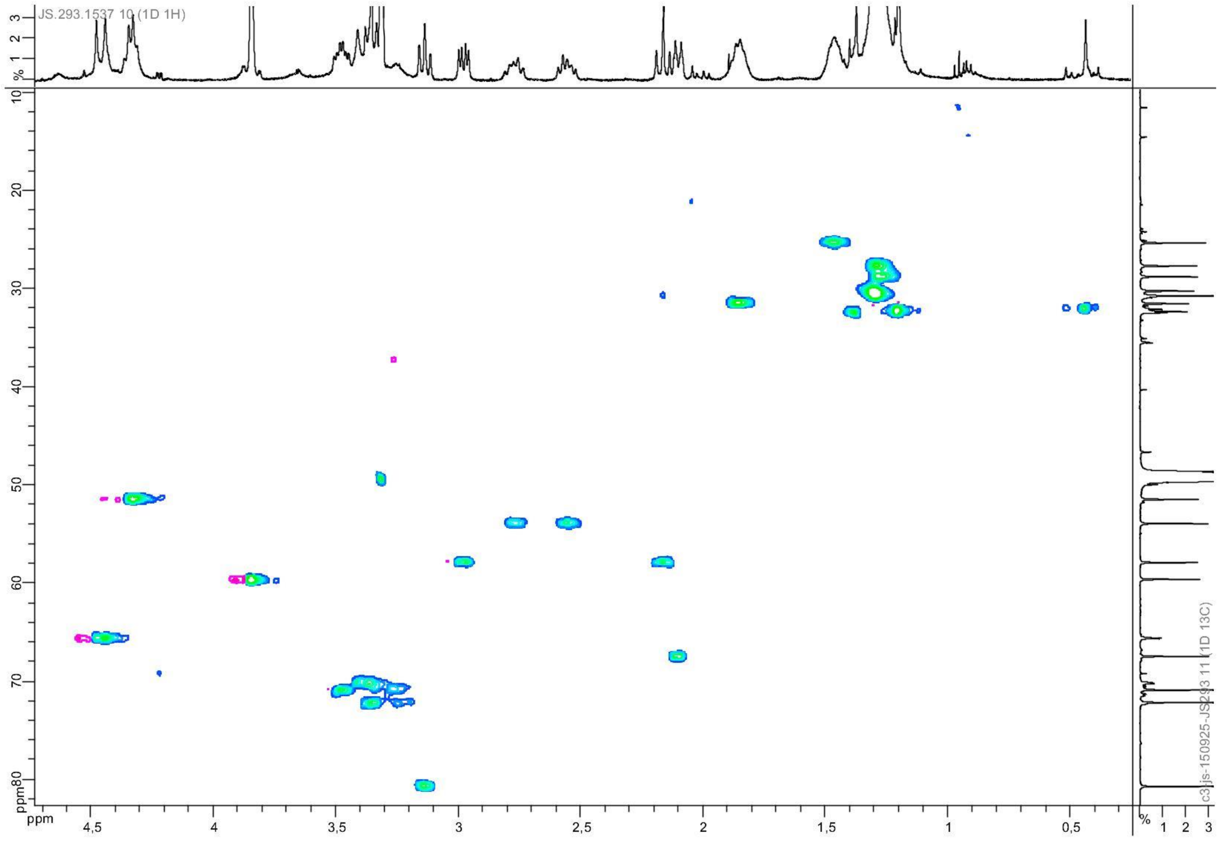

Figure S82. 2D-HSQC NMR spectrum of compound **7c** -expansion (CD_3OD, 400 MHz).

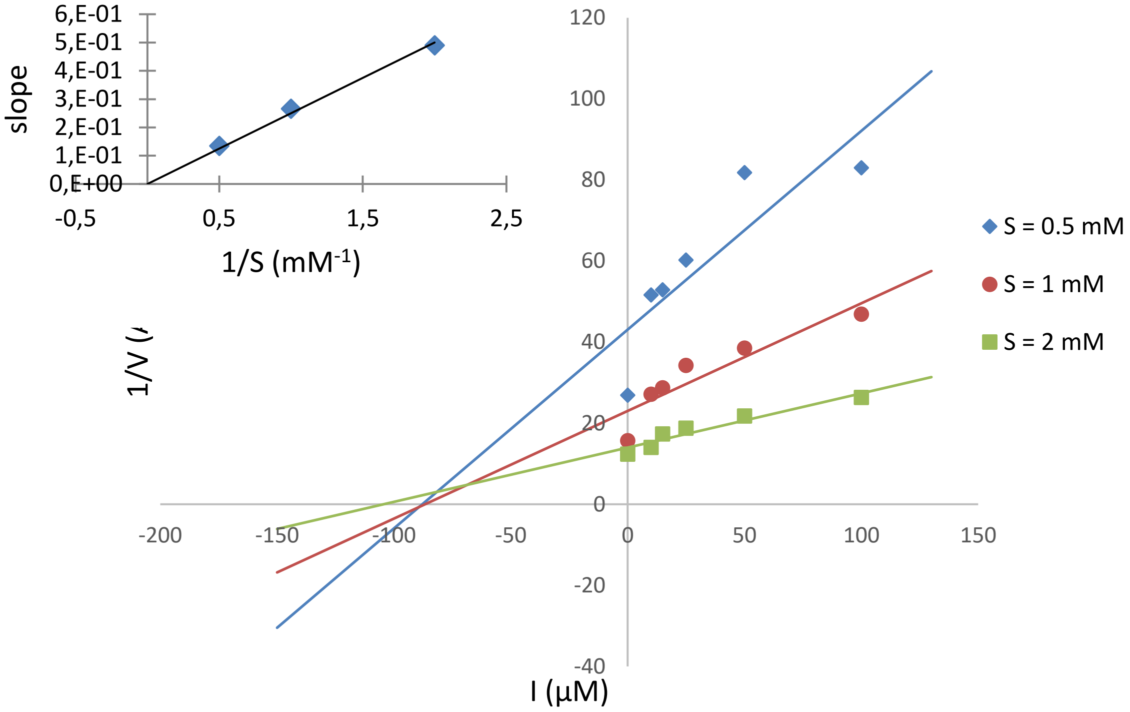

Fig. S83. Dixon plot for K_i determination of compound **6a** against JB α-man and replot of the slopes showing competitive mode. K_i = 80 ± 24 µM

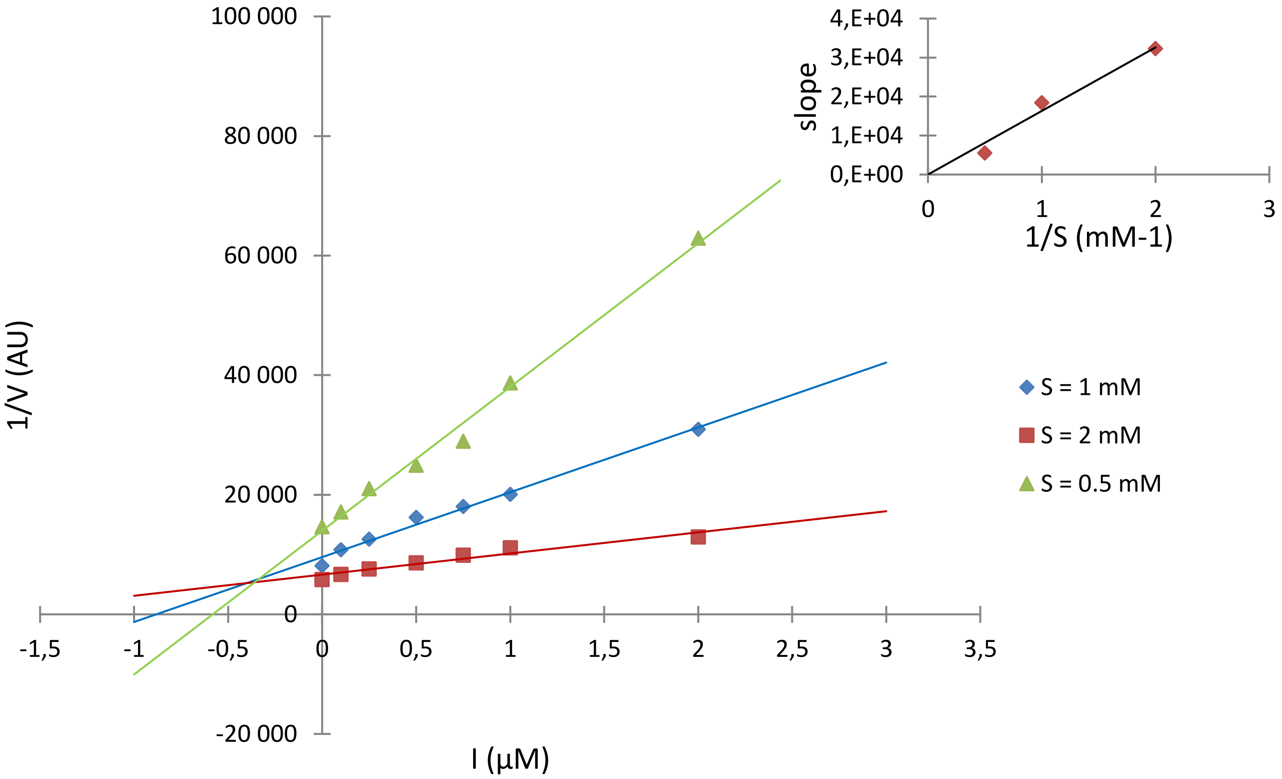

Fig. S84. Dixon plot for K_i determination of compound **6b** against JB α-man and replot of the slopes showing competitive mode. K_i = 0.32 ± 0.05 µM

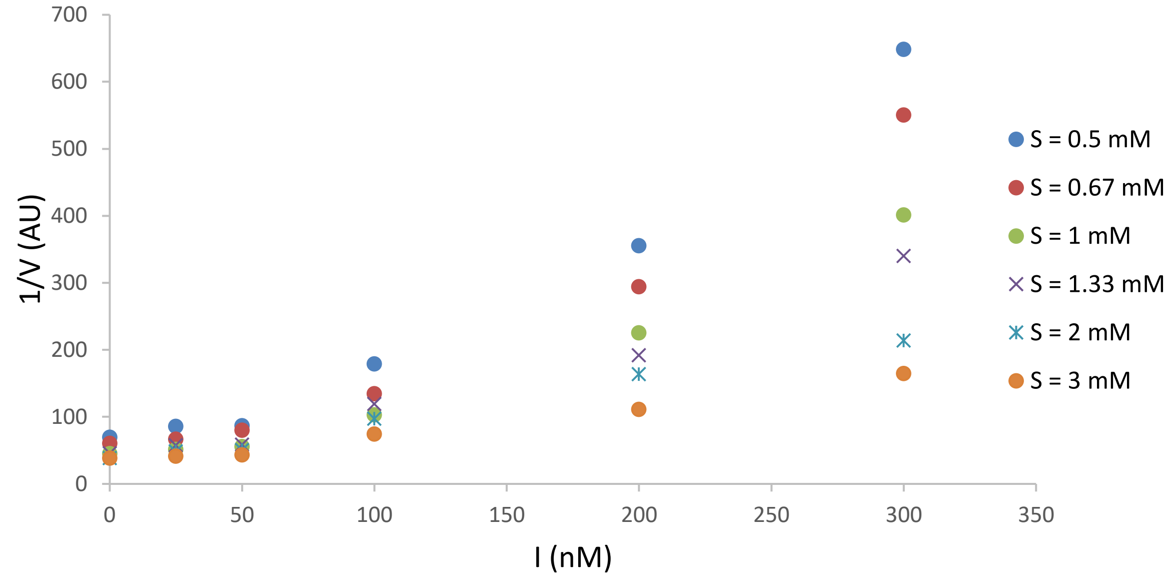

Fig. 85 : Dixon plot compound **6c** against JB α-man showing a strong non linearity.

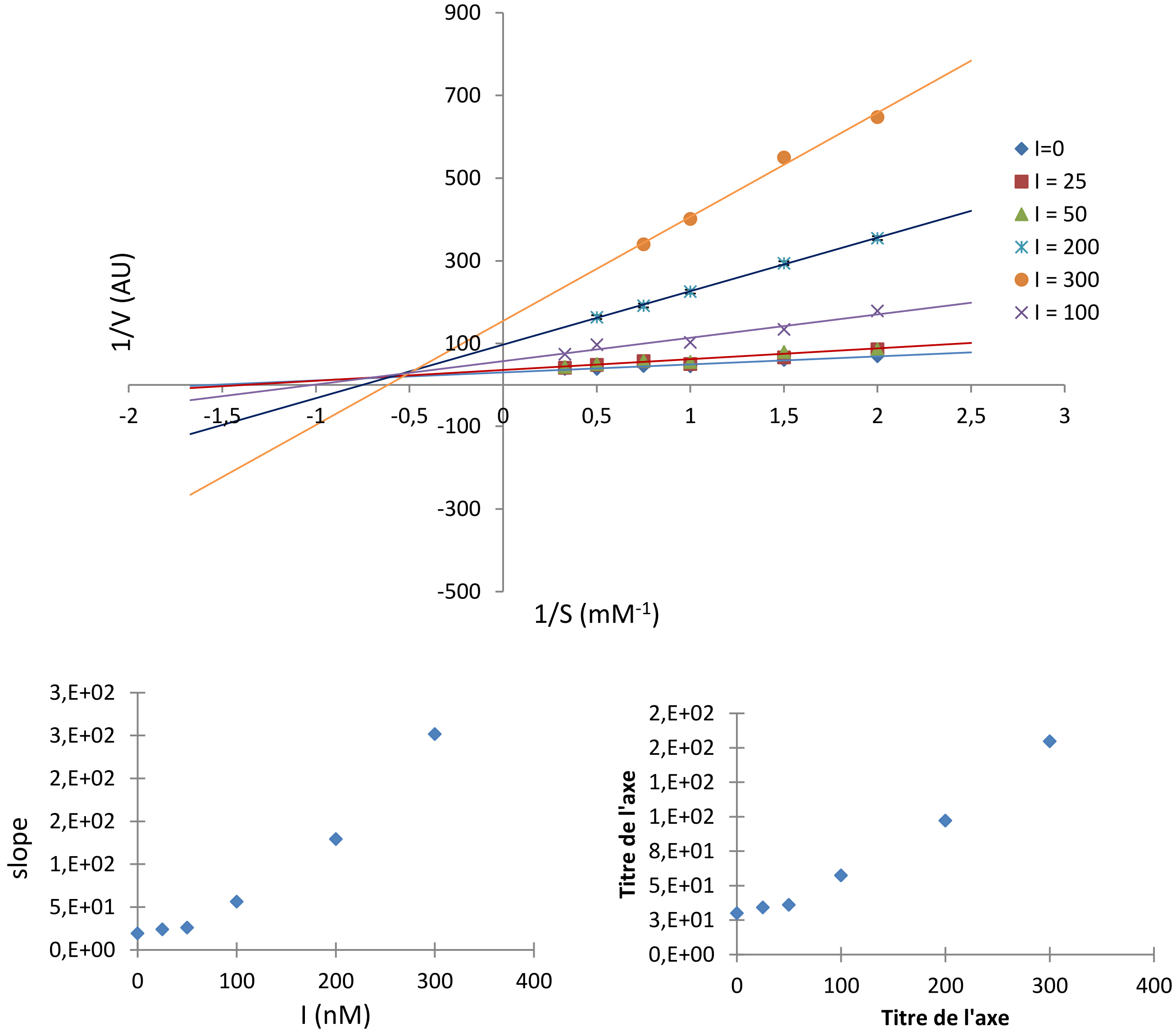

Fig. S86. Lineweaver-Burk plot of compound **6c** against JB α-man and the corresponding replots that are non linear.

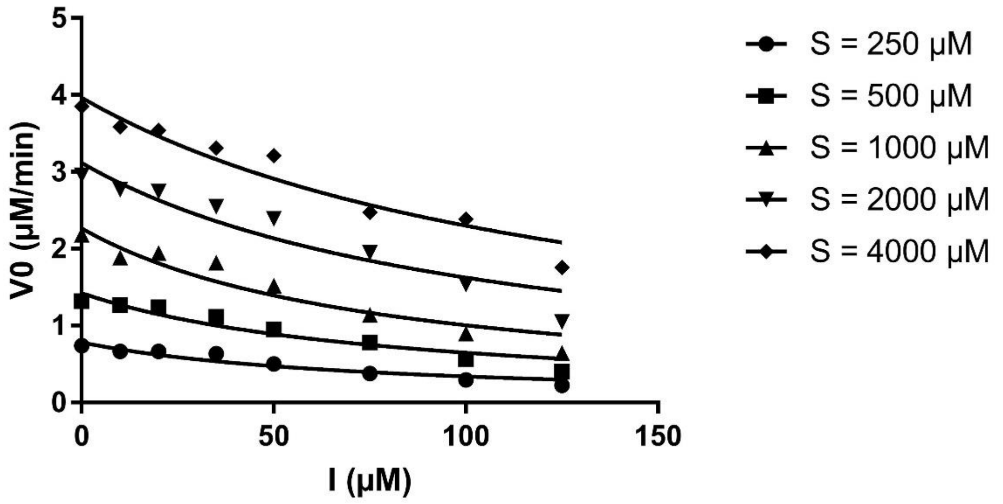

Fig. S87 : Plot of enzyme velocity as a function of inhibitor **6c** concentration. The solid curves drawn through the data points represent the best fit to the Morrison equation used to obtain K_i value (50 ± 12 nM) for the tight binding inhibitor **6c.**

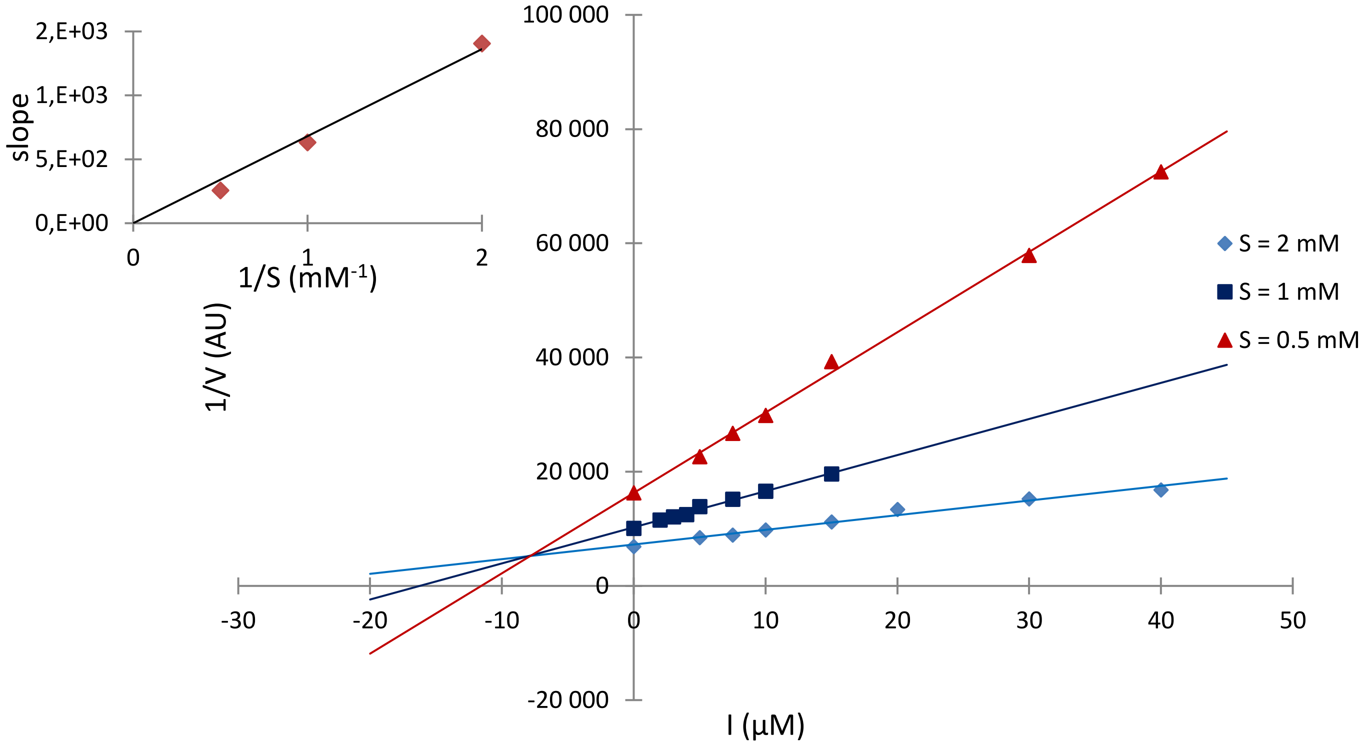

Fig. S88. Dixon plot for K_i determination of compound **7a** against JB α-man and replot of the slopes showing competitive mode. K_i = 7.7 ± 0.7 µM

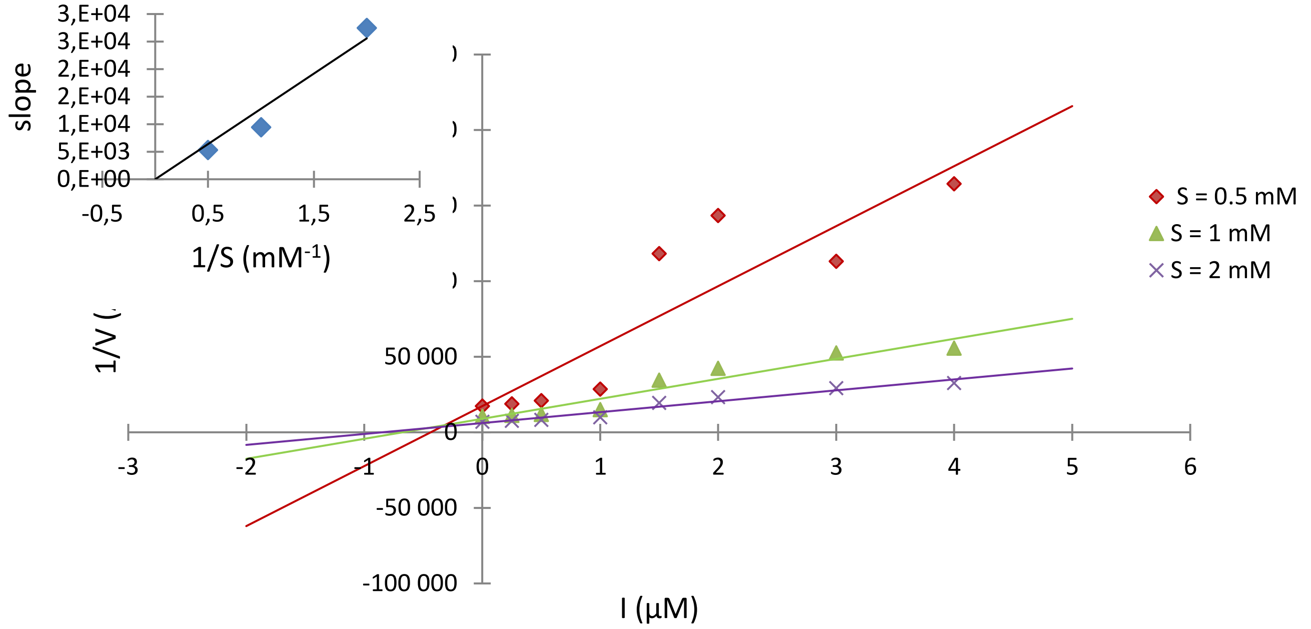

Fig.

Fig. S89. Dixon plot for K_i determination of compound **7b** against JB α-man and replot of the slopes showing competitive mode. K_i = 0.38 ± 0.01 µM

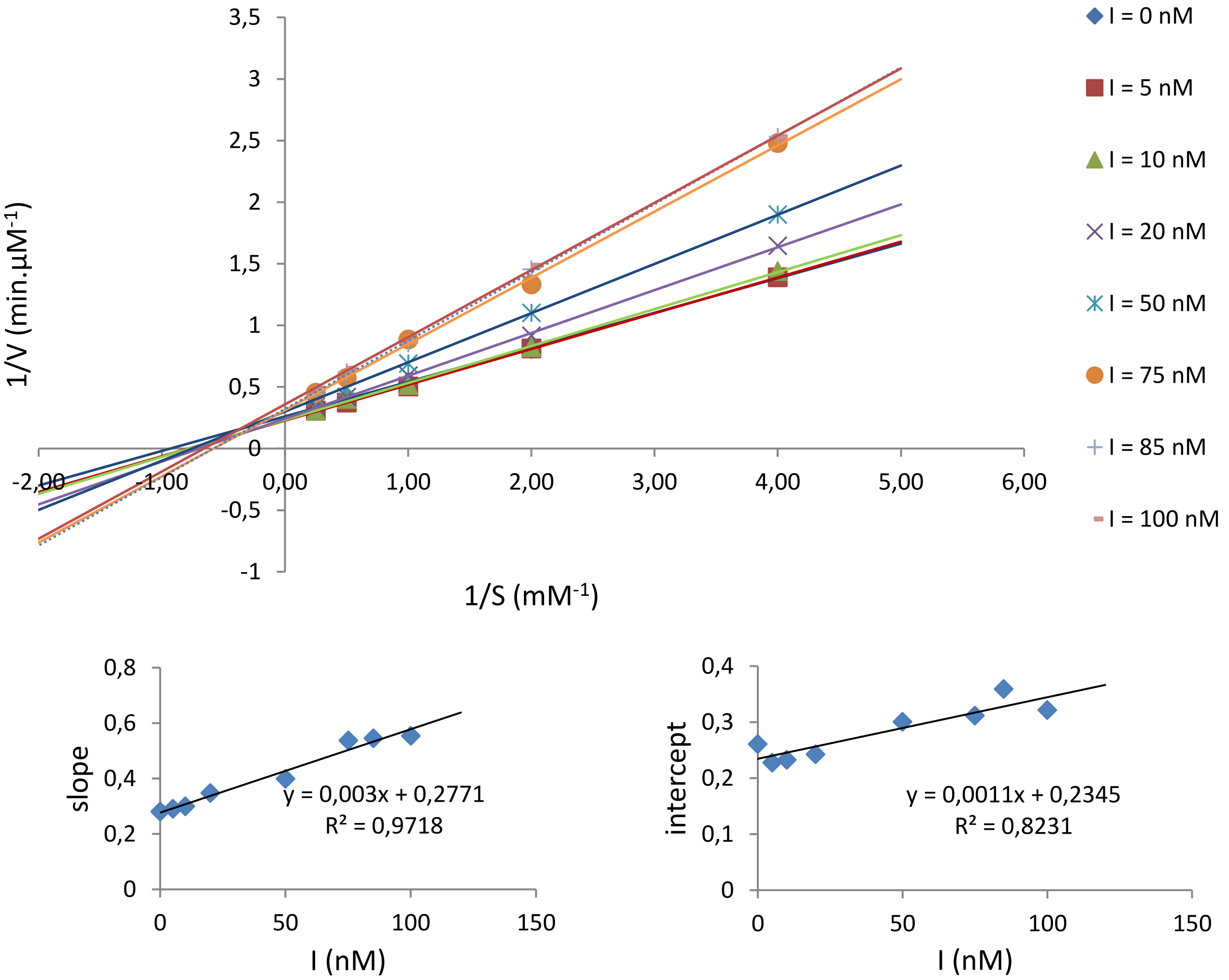

Fig. S90. Lineweaver-Burk plot for K_i determination of compound **7c** against JB α-man. K_i = 92 ± 8 nM, K_i' = 213 ± 42 nM.

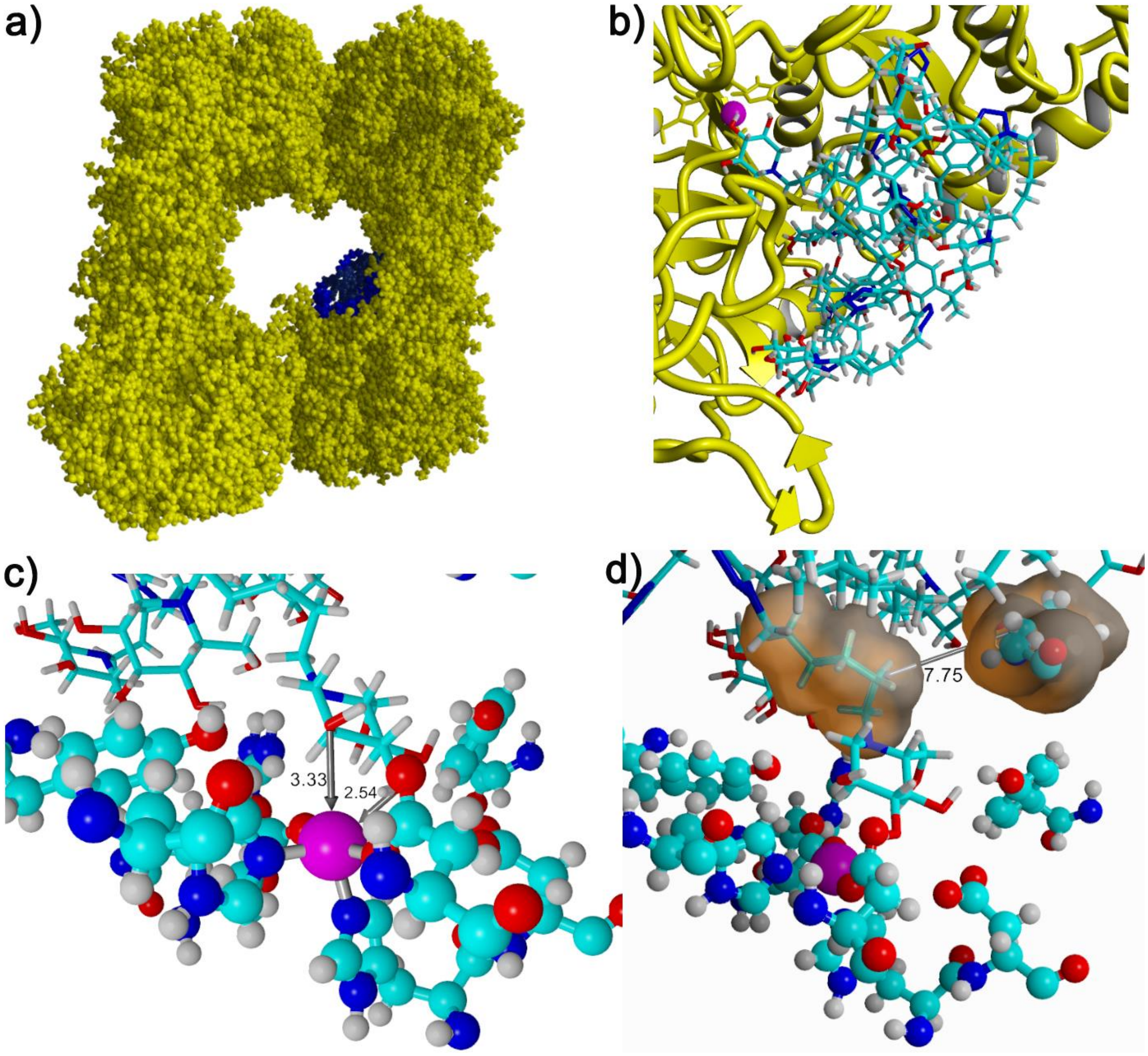

Fig. S91. Docking experiments. (a and b) 3D model of the binding mode of **6a** with JBα-man. In (a) the ligand **6a** is given in blue while the protein is colored in yellow. (b) Magnification of the interaction with ligand **6a.** (c) Detailed view of the octahedral coordination of Zn ion (in magenta) in enzyme pocket: the coordination of Zn ion with the oxygen atoms of the iminosugar is highlighted. (d) Detailed view on the search of hydrophobic interactions between the C6 chain of the Zn-coordinated DNJ of cluster **6a** with glycine residues. The only remaining interaction is the one with G790. Distance between G788 and the alkyl chain is pointed out to show that this interaction is not taking place here.

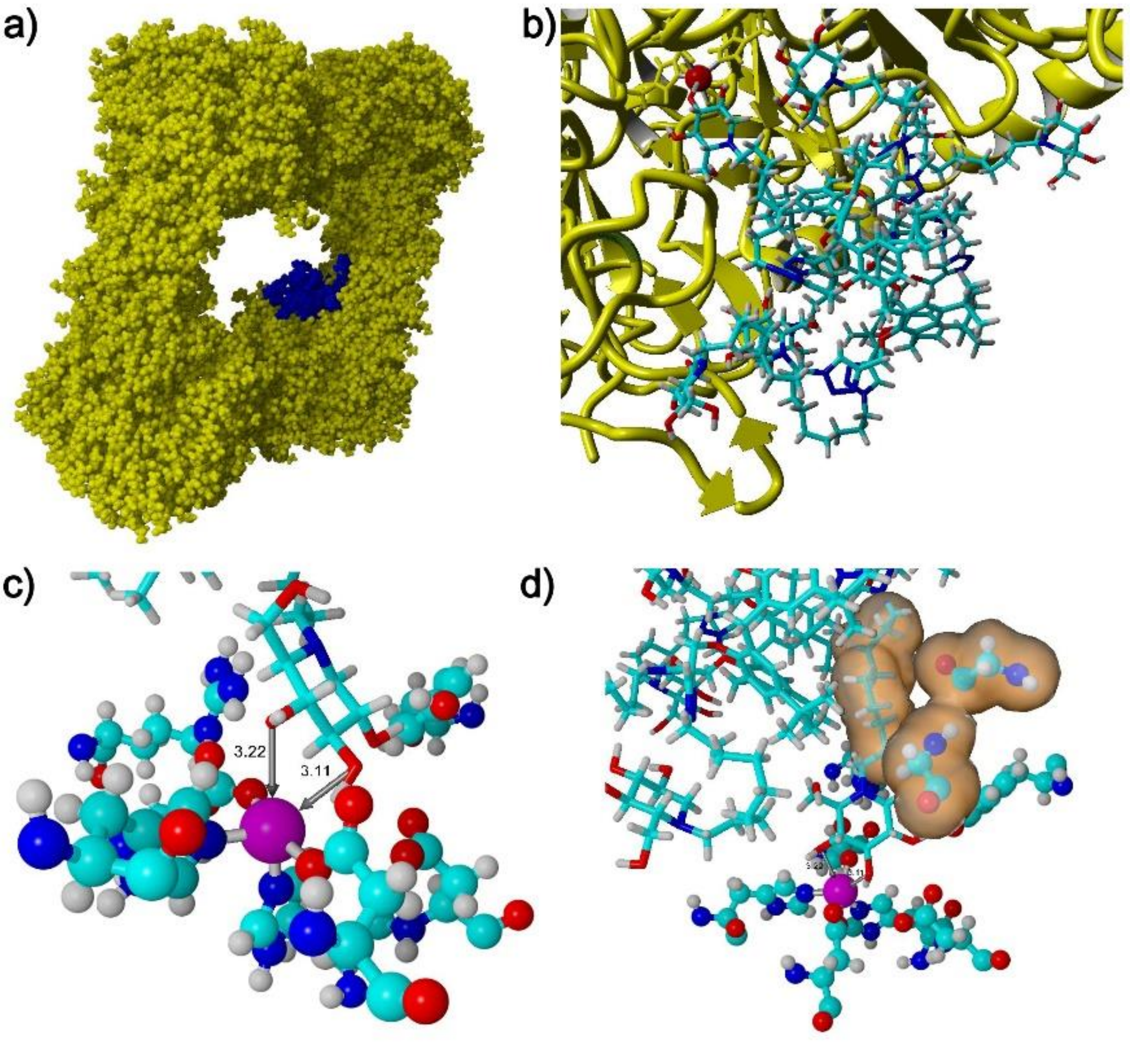

Fig. S92. Docking experiments. (a and b) 3D model of the binding mode of **6b** with JBα-man. In (a) the ligand **6b** is given in blue while the protein is colored in yellow. (b) Magnification of the interaction with ligand **6b.** (c) Detailed view of the octahedral coordination of Zn ion (in magenta) in enzyme pocket: the coordination of Zn ion with the oxygen atoms of the iminosugar is highlighted. (d) Detailed view of the van der Waals contacts between the hydrophobic chain of the Zn-coordinated DNJ of cluster **6b** with G788 and G790.
